# PRKCE non-coding variants influence on transcription as well as translation of its gene

**DOI:** 10.1080/15476286.2022.2139110

**Published:** 2022-10-26

**Authors:** Khushbukhat Khan, Sameen Zafar, Amna Hafeez, Yasmin Badshah, Kanza Shahid, Naeem Mahmood Ashraf, Maria Shabbir

**Affiliations:** a Department of Healthcare Biotechnology, Atta-ur-Rahman School of Applied Biosciences, National University of Sciences and Technology, Islamabad, Pakistan; b School of Biochemistry & Biotechnology, University of the Punjab, Lahore, Pakistan

**Keywords:** Protein kinase C epsilon, untranslated region variations, transcription factors, gene expression, Transcription

## Abstract

Untranslated regions of the gene play a crucial role in gene expression regulation at mRNA and protein levels. Mutations at UTRs impact expression by altering transcription factor binding, transcriptional/translational efficacy, miRNA-mediated gene regulation, mRNA secondary structure, ribosomal translocation, and stability. PKCε, a serine/threonine kinase, is aberrantly expressed in numerous diseases such as cardiovascular disorders, neurological disorders, and cancers; its probable cause is unknown. Therefore, in the current study, the influence of PRKCE 5’-and 3'UTR variants was explored for their potential impact on its transcription and translation through several bioinformatics approaches. UTR variants data was obtained through different databases and initially evaluated for their regulatory function. Variants with regulatory function were then studied for their effect on PRKCE binding with transcription factors (TF) and miRNAs, as well as their impact on mRNA secondary structure. Study outcomes indicated the regulatory function of 73 5'UTR and 17 3'UTR variants out of 376. 5'UTR variants introduced AP1 binding sites and promoted the PRKCE transcription. Four 3'UTR variants introduced a circular secondary structure, increasing PRKCE translational efficacy. A region in 5'UTR position 45,651,564 to 45,651,644 was found where variants readily influenced the miRNA-PRKCE mRNA binding. The study further highlighted a PKCε-regulated feedback loop mechanism that induces the activity of TFs, promoting its gene transcription. The study provides foundations for experimentation to understand these variants’ role in diseases. These variants can also serve as the genetic markers for different diseases’ diagnoses after validation at the cell and population levels.

## Introduction

Genetic variants are associated with developing several complex diseases, including cardiovascular diseases, metabolic disorders, and cancers. Various studies have indicated the impact of these variants on the gene expression and protein functioning that affects the cell’s molecular players’ activity and contributes to diseases. Characterization of a gene variant role helps determine the pathogenic variants as a prognostic or diagnostic marker for a particular disease[1]. In the past decade, the investigations for delineating disease-causing variants have coupled molecular biology experiments such as next-generation sequencing with bioinformatics approaches [2]. Such approaches have helped provide direction to research and save time, energy, and material and financial resources. Previously, pathogenic variants in CTLA4 and IL-4 genes were evaluated through this coupled approach [3].

PKCε, encoded by the PRKCE gene, is a member of the nPKC family that requires DAG/PE for its activation. Several studies have validated the association of PKCε with cardiac, metabolic, and neurological diseases and various cancers [4–14]. Genetic variants in PRKCA (rs9909004), PRKCQ (rs571715), PRKCI (rs546950 and rs4955720), PRKCG (rs3745406), PRKCD (rs2306574), PRKCH (rs2230500), PRKCE (rs940052) have shown an association with the progression of different types of cancers ^10−14^. Previously, the regulatory sequence variants of PRKCE have been studied and analyzed for their disease association. PRKCE variant rs4953299 is a part of the VEGF pathway and leads to tumour survival in colon cancer [15]. Another intronic variant of PRKCE rs940052 was also reported to be associated with radiation toxicity in lung cancer [16]. Nevertheless, the data regarding the involvement and association of PRKCE regulatory sequence variants with different diseases are very scarce and need further investigation.

Recently, extensive *In silico* analysis of non-synonymous variants in PRKCE has been performed, revealing the presence of non-synonymous polymorphic variants in different regions of this protein, resulting in altered proteins structure and functions along with altered structural dynamics, affecting its molecular interactions and mode of activation [17].

Various studies have validated that upregulated PKCε plays a vital role in cancer development and progression. However, no study has ever been conducted that explores the impact of PRKCE non-coding variants on the transcriptional dysregulation of this gene and contributes to its aberrant expression in different diseases. Hence, the current study aimed to elucidate the effect of non-coding variants, specifically UTR variants, on the transcription and translation of PKCε that may contribute to its dysregulated expression. The study further, aimed to predict the UTR variants’ influence on the secondary structure of PRKCE mRNA and its potential role in altering UTRs’ binding affinity with complementary miRNAs. A molecular pathway that impacted the activity of transcription factors essential for PRKCE transcription was also constructed. The outcomes of the present study are of significance in exploringPRKCE as a prognostic and therapeutic target for the diseases. This study provides foundations for further studies related to exploring PRKCE as a potential diagnostic marker for human diseases, especially cancers.

## Materials and methods

### Data retrieval and data sorting

Variant data of PRKCE gene belonging to all consequences was retrieved from ENSEMBL (that included SNP data from dbSNP database [18,19], COSMIC (Catalogue Of Somatic Mutations In Cancer) [20], EVS (NHLBI GO Exome Sequencing Project’s Exome variant Server [21], and genomeAD [22]. All the SNPs were mapped on genome assembly GRCh38/hg38, and information regarding PRKCE gene sequence, variant IDs, genomic coordinates, and allele alterations were obtained from these databases. Variant data consisted of two main consequences: coding region variants and non-coding region variants. As the study objective revolved around non-coding variants, coding region variants were ignored. Non-coding variants consisted of intronic variants, 5'UTR and 3’ UTR variants, and splice site variants. We sorted UTR variants from all four databases and scrutinized variant data for redundancy. Only variants having rsIDs were picked, and the rest were ignored. The data was accessed in March 2022.

### Regulatory function analysis

RegulomeDB [23] was used to identify variants with high regulatory potential. RegulomeDB classifies variants according to their regulatory potential as per experimental evidence from ENCODE and other databases and computational predictions based on mutual annotations into seven major categories, among which categories 1, 2, and 3 are subdivided as per their functional consequence. RegulomeDB also gives a regulome score to each variant investigated, where a score of 1 or closer to 1 indicates high confidence in the assigned rank to the variant. An explanation of RegulomeDB ranks is provided in Table 1.

**Table 1.** Description of different ranks of Regulomedb.

Ranks	Description
1a	eQTL + TF binding + matched TF motif + matched DNase Footprint + DNase peak
1b	eQTL + TF binding + any motif + DNase Footprint + DNase peak
1c	eQTL + TF binding + matched TF motif + DNase peak
1d	eQTL + TF binding + any motif + DNase peak
1e	eQTL + TF binding + matched TF motif
1 f	eQTL + TF binding/DNase peak
2a	TF binding + matched TF motif + matched DNase Footprint + DNase peak
2b	TF binding + any motif + DNase Footprint + DNase peak
2c	TF binding + matched TF motif + DNase peak
3a	TF binding + any motif + DNase peak
3b	TF binding + matched TF motif
4	TF binding + DNase peak
5	TF binding or DNase peak
6	Motif hit
7	Other

eQTL = expression quantitative trait loci, TF = transcription factor, DNase = deoxyribonuclease

### Allele frequency calculation and evolutionary conservation prediction

Allele and genotypic frequency of PRKCE 5’ and 3'UTR were also investigated through projects: gnomAD genomes v3.2.1 [24], NCBI ALFA [25], Trans-Omics for Precision Medicine (TOPMed) [26], and 1000 genome project phase 3 [27]. Minor allele frequency (MAF) was also calculated. That indicated the second most frequent allele. Similarly, the change tolerance of the variants was also predicted through CADD and GERP [28]. The higher the score of GERP, the more conserved the allele is.

### Transcription factor binding site analysis

Prediction of transcription factor binding sites was performed using Alibaba 2.0 software (www.gene-regulation.com). The tool employs information from the TRANSFAC database and is processed through EMBL. Moreover, for the identification of TFBS, the tool aligns the known binding sites with unknown binding sites through pair-wise alignment. It constructs a matrix to predict new binding sites due to variations or mutations. Input is given as FASTA format in the tool. The output consists of segment information that depicts potential binding sites, start and end sites of binding sites for TF, and information regarding TF-binding sites [29].

### Transcription factors co-regulation analysis

A database named TIGER [30–32] (Tissue-specific Gene Expression and Regulation), designed by Bioinformatics Lab at Wilmer Eye Institute of Johns Hopkins University, was accessed to predict the coordination of TFs with each other for PRKCE gene transcription. The database provided information regarding TFs’ co-regulation along with -LogP values. -LogP value depicted the distribution of TFs as well as co-TFs in various tissues. The database also determined the distribution of co-regulatory TFs in different tissues.

### Pathway construction

Transcription factors that were predicted to regulate gene expression of PRKCE were also analysed to determine their upstream molecules that induce their activity. Identification of upstream molecules and cellular cascades they participate in were identified through pathway mapping and gene annotations from the Kyoto Encyclopaedia of Genes and Genomes (KEGG) database [33] and Gene ontology (GO) database [34]. An illustration of the pathway was drawn using Inkscape drawing tool [35].

### RNA secondary structure prediction

The secondary structure of mRNA is vital in transcription and translation. Variants influence the mRNA secondary structure as well as overall positional entropy. To investigate the impact of 5’ and 3’ UTR variants influence on the PRKCE mRNA secondary structure, web server-based tools, RNAstructure [36,37] and RNA fold [38], were used. Using the dynamic programming algorithms, RNA fold predicts the minimum free energy (MFE) secondary structure of single mRNA sequences. It also calculates equilibrium base-pairing probabilities through John McCaskill’s partition function (PF) algorithm. The sequence of RNA or DNA (single-stranded) is fed as FASTA format in the software.

Similarly, RNAstructure employs separate analysis and prediction algorithms: pseudoknot prediction, finding structures with maximum expected accuracy, calculating a partition function, and predicting a minimum free energy (MFE) structure. The input of RNA/DNA sequence is in FASTA format, and output consists of probability-annotated secondary structures based on the lowest free energy and probability of correctness. Minimum free energy values of both tools were computed for their significance through a parametric t-test applied using GraphPad Prism 8 [39]. Structures with P-value less than 0.05 were chosen for further analysis.

### PRKCE mRNA-miRNA interaction analysis

To establish the effect of PKCε variants on microRNA interaction with mRNA, PKCε miRNA data were retrieved using miRGate [40,41], a database containing experimentally validated as well as computationally predicted miRNA–mRNA pairs [40]. SNP effect on mRNA-miRNA interaction was elucidated using RNAhybrid [42], which indicates the miRNA targets based on minimum free energy through determining the most favourable hybridization sites between two RNA sequences.

### PRKCE UTR variants disease association

The association of PRKCE variants is also predicted through the rSNP base 3 tool that employs the algorithms from ENCODE, miRbase, Lncipedia, Circnet, TargetScan, miRnada, HapMap, GWAS catalogue, and HGMD. The tool takes variant information from ENSEMBL and creates variant-specific annotations (http://rsnp3.psych.ac.cn/search.do). Developmental Genotype-Tissue Expression (dGTEx) Project was employed to assess the impact of PRKCE variants on its gene expression. The data used in the present study was obtained from the GTEx portal and accessed on October 1st, 2022 (accession number phs000424.vN.pN).

## Results

### PRKCE UTR variants count

**Figure 1.**
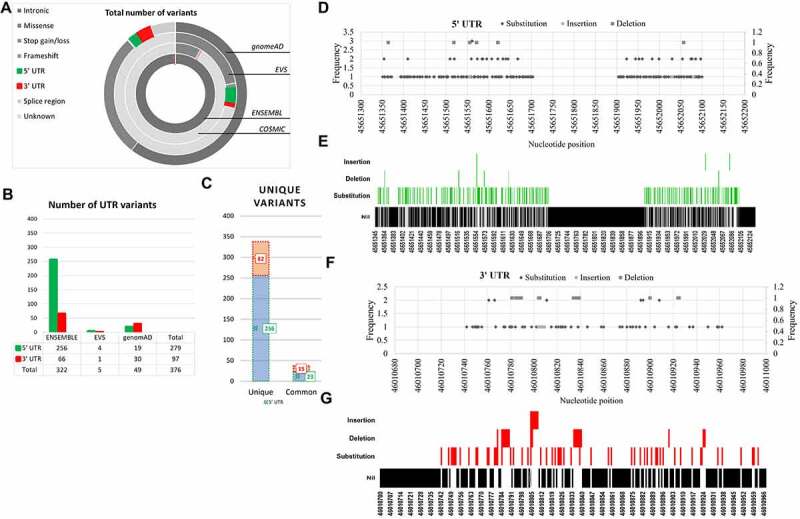
**PRKCE variants data retrieved from different databases (ENSEMBL, COSMIC, EVS, and genomeAD).** (A) Total variants obtained for PRKCE gene from all databases were 346, 853. Out of which 376 were UTR variants. b(B) Number of variants belonging to different consequences, retrieved from different databases. c(C) UTR (3’ and 5’) variant number obtained from different databases. Among these 256 and 82 unique 5’ and 3’ UTR variants were sorted, respectively. 5'UTRs are highlighted with green and 3’ UTRs are highlighted with red. d(D) 5'UTR variation frequency and (Ee) heatmap for mutational hotspot. (Ff) 3'UTR variation frequency and (G) heatmap for mutational hotspot.

PRKCE UTRs’ variant data was accessed through four databases (ENSEMBL, COSMIC, EVS, and GenomeAD). Out of 346,853 variants, UTRs’ variants were sorted and assessed for unique variants (Fig. 1a). The total number of variants retrieved for the PRKCE gene from each database was ENSEMBL 343047, COSMIC 2589, genomeAD 1093, and EVS 124. These variants belonged to two major consequences: coding region variants and non-coding region variants. UTR variants come under the umbrella of non-coding variants. Among the non-coding variants, the proportion of UTRs variant data was ENSEMBL 0.1%, EVS 4%, and genomeAD 4.4%, whereas the COSMIC database contained no information on PRKCE UTR variants (Fig. 1A and supplementary Table S1). A total of 376 variants for UTR were found, among which 256 5'UTR and 82 3'UTR variants were found to be unique (Fig. 1b,c).

### Mutational hotspots in UTR region

Relative abundance analysis of 256 5'UTR and 82 3'UTR variants of the PRKCE gene revealed that a great proportion of variants at both 5’ and 3'UTR were substitution variants (82.8% and 74.3%, respectively). Furthermore, among 791 residues at 5'UTR, variants were mapped on approximately 25.92% of residues, and among these residues, 94.6% are prone to substitution, 1.4% to insertion, and 3.4% to deletion. At 3'UTR, 30.4% of residues out of 268 were prone to mutation. Among the 30.4% residues, 68.29% went through substitution, 8.5% insertion, and 23.1% deletion. At PRKCE 5'UTR, most variants were concentrated at position 45,651,550 to 45,651,650, whereas position 46,010,779 to 46,010,820 at PRKCE 3'UTR was a hub for most variants. The analysis indicated mutational hotspots at 5'UTR and 3'UTR of the PRKCE gene and suggested that 3'UTR is more prone to variations compared to 5'UTR. Fig. 1d–g illustrates the graphical representation of the frequency of variations and mutational hotspots at PRKCE UTRs.

### PRKCE 5’ and 3’ UTRs’ variant regulatory function analysis

**Figure 2.**
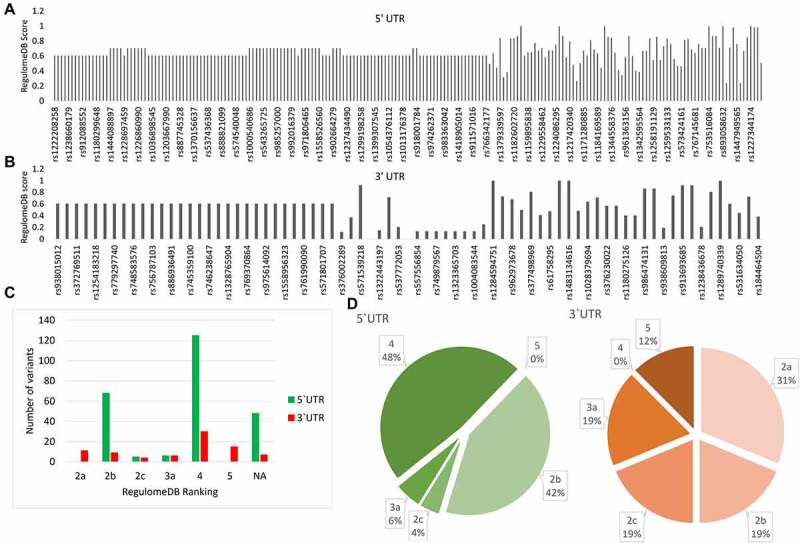
**RegulomeDB analysis of 5’ and 3’ UTR variants of PRKCE gene**. RegulomeDB probability score of 5 UTR variants (A) and 3'UTR variants (B) of PRKCE. Score near one or one indicates high probability of variant being regulatory variant. (C) Distribution of PRKCE UTR variants in six regulomeDB ranks (2a, 2b, 2c, 3a, 4, and 5). (D) Number of variants in different regulomeDB ranks having probability score more than 0.7.

PRKCE 5’ and 3’ UTR variants were investigated for their potential impact on the regulatory elements, including transcription factor binding sites (TFBSs), promoter region, and DNase hypersensitive region in the PRKCE gene. RegulomeDB distributed PRKCE UTR variants into six classes (2a, 2b, 2c, 3a, 4, and 5) and assigned a probability score to each variant where a score near to 1 depicted the high probability of the variant being a regulatory variant (Fig. 2a, 2b, & 2c; Supplementary table 2). RegulomeDB did not predict the rank of forty eight 5'UTR and seven 3'UTR variants. Based on the probability score, PRKCE UTR variants having scores more than 0.7 were selected for further analysis. A total of 73 5'UTR variants and 17 3'UTR variants had a score above 0.7. After filtration, 5'UTRs were mainly distributed in ranks 2b, 2c, 3a, and 4, while 3'UTR variants were ranked in 2a, 2b, 2c, 3a, and 5 regulomeDB ranks (Fig. 2d; supplementary Table S3).

The distribution of variants in ranks 2a, 2b, 2c, 3a, 4, and 5 suggests that variants influence the TFBS and Dnase I hypersensitivity site. Rank 2a and 2c also suggest that the variant lies in a region with matched transcription factor binding motif, whereas rank 3a depicts that variants affect the conserved DNA motif. Overall, regulomeDB analysis revealed that sorted 73 5'UTR and 17 3'UTR variants have a high probability of impacting transcription of PRKCE gene by modulating the interaction of trans-acting factors, specifically transcription factors with TFBSs on PRKCE 5’ and 3'UTR region.

### Allele frequency estimation and allele change tolerance analysis

RegulomeDB-sorted variants were also evaluated for the MAF analysis and change tolerance to gain insight into their evolutionary conservation status and potential pathogenicity. MAF values and genotypic frequencies indicated that altered alleles are less frequently present in the population than ancestral or wildtype alleles. However, 5'UTR variants: rs569884823 and rs543265725 ancestral alleles’ MAF score were <0.01 indicating their second most frequent allele status (Supplementary table 3). Moreover, the studied alleles have moderate evolutionary conservation scores indicating their pathogenicity.

### Impact of UTR variants on transcription factor binding sites of PRKCE

**Figure 3.**
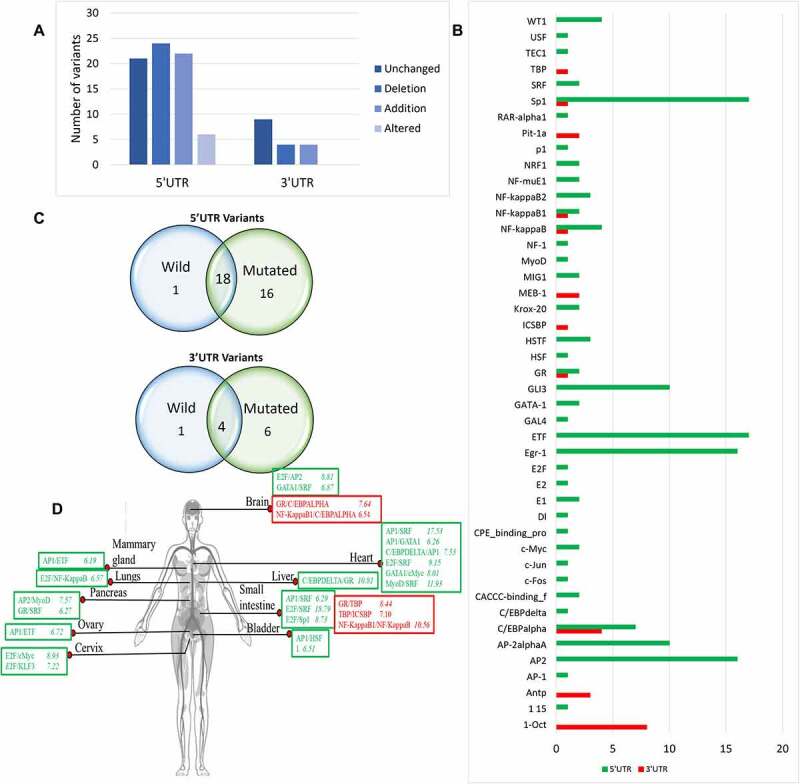
Impact of PRKCE UTR variations on the transcription binding sites of the gene. (A) Most of the PRKCE 5'UTR variants caused the deletion of the transcription factor binding sites and most 3'UTR variants did not have impact on transcription factor binding sites. (B) Graph representing the transcription factor binding sites introduced due to UTR variations. (C) Venn diagram representation of the transcription factor binding site specifically present in Wildtype and mutated sequences and common is both wildtype and mutated PRKCE gene sequence due to 5’ and 3’ UTR variation. (D) Co-regulation mechanism of transcription factors in different tissues in humans. Transcription factors in red box represent sites present at 5'UTR and in green box depicts 3'UTR sites. Number written next to transcription factor complex indicates -LogP value where higher value indicates significance.

Transcription factor binding sites (TFBS) are present on DNA that binds with specific transcription factors (TF). DNA sequence variations either enhance the TF’s ability to bind with TFBS or decrease the TF-TFBS interaction. UTR variants of PRKCE indicated to be present in TFBS were then further evaluated for their impact on TF binding with PRKCE UTRs. Analysis through the webserver-based tool, AliBaba2.0, predicted that out of 73, 22 5'UTR variants induced the TFBS insertion, 24 variants deleted the TFBS, and 4 variants replaced the TFBS, whereas 21 variants did not have any influence on the TFBS. Similarly, out of 17 3'UTR variants, only 4 variants altered and deleted the TFBS (Fig. 3a; Supplementary table 4a & 4b). The analysis further showed that these variants induced the introduction of 11 new TFBSs in 3'UTR and 43 in the 5'UTR region. Among these, the 1-Oct binding site was more frequently added due to the 3'UTR variants and AP2-, Egr-1-, and Sp1-binding sites were frequently introduced due to the 5'UTR variants (Fig. 3b).

Comparative analysis of TFBSs found in both wildtype and mutated PRKCE sequences indicated that one TFBS was unique to 5'UTR of wildtype gene and 16 were unique to mutated sequences of the PRKCE gene. Similarly, 6 TFBSs were unique to mutated 3'UTR sequences of PRKCE gene. (Fig. 3c). TFBSs TEC1-binding site and AntP-binding site were found explicitly in wild PRKCE 5'UTR and 3'UTR, respectively. TFBSs (GR, Sp1, NF-kappaB, NF-kappaB1, ICSBP, and TBP) were mainly introduced at 3'UTR due to variations, and sites AP-1, C/EBPdelta, KLFs, c-Fos, c-Jun, c-Mys, CPE binding protein, DI, E1, E2F, GAL4, GATA-1, GR, MIG1, MyoD, and P1 were solely present at the mutated PRKCE 5'UTR.

The coordinated interaction of several TFs modulates the expression of the genes [43]. In the present study, the combinatorial regulation of PRKCE gene expression is also predicted. TFBSs introduced due to UTR variants were targeted to get an insight behind the potential gene transcription machinery. The annotation from Tiger database indicated that the combination of TFs in regulating transcription varies in different tissues (Fig. 3d). For instance, TF AP1 interacts with ETF in mammary glands (-LogP 6.19) and ovaries (-LogP 6.72), whereas AP1 binds with GATA1 and HSF1 in heart (-LogP 6.26) and bladder (-LogP 6.51). The distribution of TF and co-TF varies from tissue to tissue that can be estimated through -LogP value. AP1 and SRF both are present in heart and small intestine; however, they are more readily present in heart (-LogP 15.53) than small intestine (-LogP 6.29). It is also observed that one TF binds with more than one co-TF to regulate the gene expression. In muscle, MyoD make a complex with NF-1, USF, and GATA-1 to co-ordinate gene expression. Similarly, E2F interacts with SRF (-LogP 18.79), sp1 (-LogP value 8.73), and Ap2 (-LogP value 6.98) in small intestine to control transcription of the gene having E2F binding site (Fig. 3d; Supplementary table 4c). Co-occurrence of these TFBSs in PRKCE UTRs due to genetic variation enhances their probability of coordination for the combinatorial regulation of PRKCE gene expression.

### Potential effect of UTR variants on the transcription of PRKCE gene

In PRKCE UTRs, more than 54 TF binding sites are present among which 22 TFBSs (5'UTR 16 and 3’ UTR 6) are specifically introduced due to the genetic variation in the PRKCE untranslated region. KEGG pathway analysis indicated that among 16 TFs whose TFBSs were detected in mutated 5'UTR of PRKCE gene, three TFs including CACCC binding factors such as KLF3, MyoD and FoxP1 act as transcription repressor, six (AP-1, cFOS, cMyc, cJun, E2F, and CREB1) are transcription activators, and KEGG did not give result of the rest of the seven TFs. Similarly, five TFs (Sp1, NF-KappaB1, NF-KappaB, ICSBP, and TBP) whose TFBSs were found in mutated 3'UTR were transcription activators (Table 2).

**Table 2.** Transcription factor binding sites (TFBS) along with the respective transcription factors and the TFBS role in transcription and pathway involve in transcription factor regulation.

Transcription factor	Transcriptional Role	Pathway	Comment	KEGG ID
**5'UTR mutated**				
AP-1	Activator[44]	Oestrogen signalling pathway	Pro-cancerous	map05224
		MAPK signalling		map05418
		IL17 signalling pathway		map04657
CACCC binding factor/KLF3	Repressor[45]	Transcriptional misregulation	Tumour suppressor	map05202
c-Fos	Activator[46].	MAPK signalling	Pro-cancerous	map04010
		Prolactin signalling pathway		map04917
c-Jun	Activator[47]	FasL pathway	Pro-apoptotic	map04210
c-Myc	Activator[48]	MAPK signalling	Pro-cancerous	map05200
CPE_binding_pro/**CREB1**	Activator[49]	Canonical wnt pathway	Pro-cancerous	map05225
		c-GMP-PKG pathway		map04022
E2F	Activator[50]	Cell cycle	Tumour suppressor	map05200
		Cell senescence		map04218
MyoD	Repressor[51]	Myogenesis	Tumour suppressor	map05017
P1/foxp1	Repressor[52]	Oestrogen signalling	Tumour suppressive	map05206
**3'UTR**				
Sp1	Activator[53]	Choline metabolism	Pro-cancerous	map05231
NF-kappaB1/p50	Activator[54]	NFKB signalling	Pro survival	map04064
NF-kappaB	Activator[55]	IL-17 signalling pathway	Pro-inflammatory	map04657
		TLR2 signalling	Pro-inflammatory	map05321
ICSBP/irf8	Activator[56]	PagP signalling	PRO-INFLAMMATORY	map05133
TBP	Activator[57]	Gene expression	Pro-cancerous	map05203

**Figure 4.**
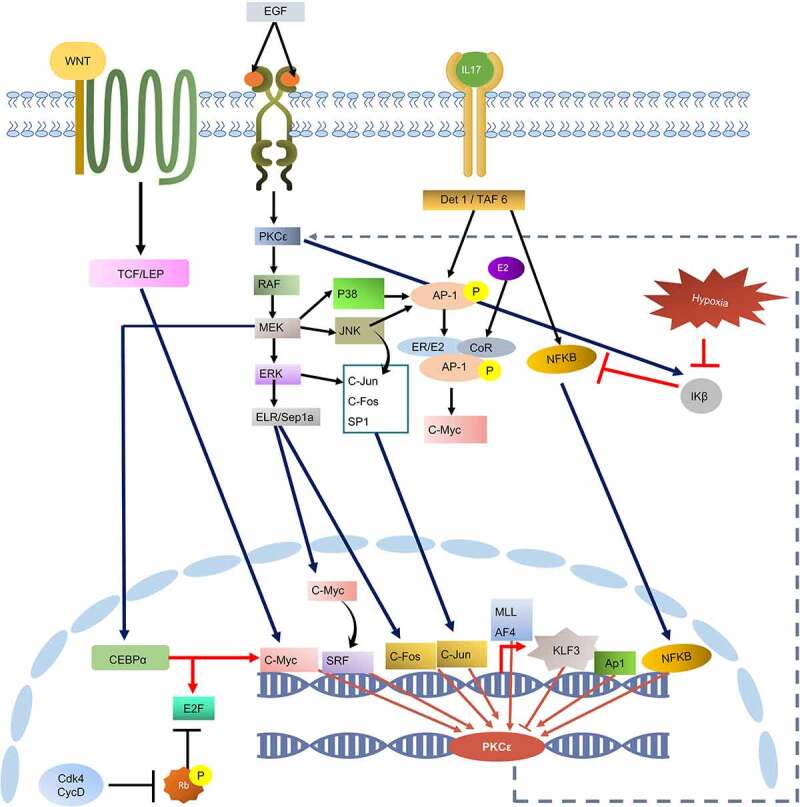
KEGG constructed pathway for the regulation of PKCε gene transcription. Cell signalling cascades such as WNT pathway, IL pathway, and EGFR/RAS/RAF pathway contributes to the activation of transcription factors that may bind with the transcription factor binding sites on PRKCE UTRs and regulate its transcription. PKCε also phosphorylates and activates RAF, through which PKCε may drive a positive feedback loop.

KEGG pathway analysis further revealed the upstream signalling cascades that modulates the activation of the transcription factors. Analysis also indicated the activity of these TFs in cancer-associated signalling pathways, particularly MAPK(ERK) signalling, hormone receptor pathways, NF-KB signalling, and IL-17 signalling (Fig. 4). MAPK signalling plays prominent role in the activation of most of these TFs such as AP-1, c-Myc, cJun, cFos, CEBPalpha, and E2F. NF- κβ is activated through IL-17 signalling. IL-17 signalling also activates AP-1. Transcription factor cMyc can be directly activated through ERK pathway, IL pathway and canonical WNT pathway. Furthermore, Transcription factor AP-1 also activates cMyc by making complex with ER/E2 heterodimer. These TFs can then activate the expression of PKCε gene by binding with the TFBS at its UTRs and may pose co-regulatory influence by interacting with other TFs. Additionally, evidence indicates that PKCε regulates the activation of MAPK signalling and NF-κβ signalling. These pathways then activate TFs that may induce the expression of PKCε. Hence, PKCε UTR variation might contribute to the establishment of a positive feedback mechanism in different diseases particularly cancer.

### PRKCE 5’ and 3’ UTR variants effect on PRKCE mRNA secondary structure

**Figure 5.**
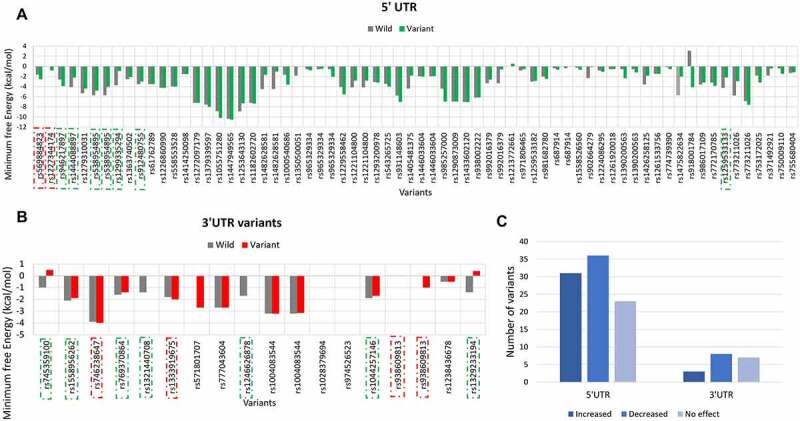
PRKCE UTR variants influence on the mRNA secondary structure. Minimum free energy (MFE) scores for both wildtype sequence and variant sequence for (A) 5'UTR variation and (B) 3'UTR variations are plotted. Lower the MFE score, higher the structure stability. (C) Number of PRKCE UTR variants affecting the mRNA stability. Major number of 5'UTR as well as 3'UTR variants decreased the mRNA structural stability. Variants that increased the mRNA stability are depicted with dotted-red square, whereas variants that decreased stability are highlighted with dotted-green square.

Secondary structure of mRNA plays essential role in pre-mRNA processing and translation. Therefore, the impact of UTR variants on the secondary structure of PRKCE mRNA was also investigated (Fig. 5a & 5b; Supplementary table 5). Based on the minimum free energy value, RNAstructure fold value, thermodynamics ensemble free energy value and ensemble frequency, it was predicted that 32 5'UTR variants increased the stability, 26 decreased, and 23 have no effect on the structure of PRKCE mRNA. Similarly, only 3 3'UTR variants increased the stability of PRKCE mRNA, 8 decreased the stability, and 7 had no effect (Fig. 5c).

**Figure 6.**
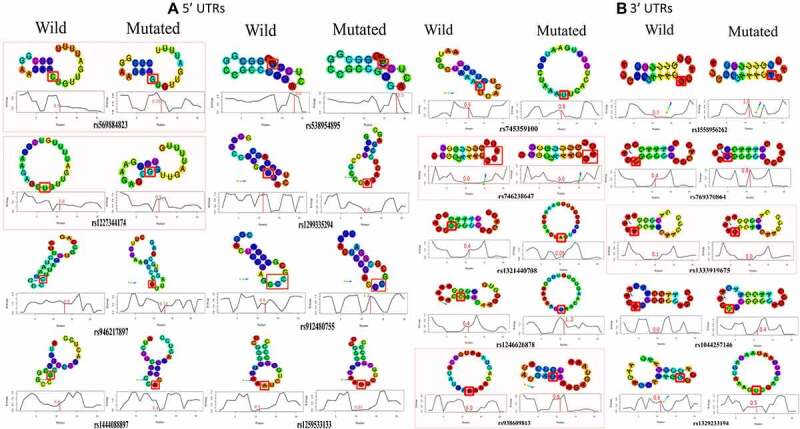
PRKCE mRNA secondary structure prediction and positional entropy alteration due to (A) 5'UTR variations and (B) 3'UTR variation. Positional entropy is depicted in different colours where red depicts lowest entropy and blue represents highest entropy (Red<Orange<Yellow<parrot green<green<cyan<blue). Variants that increased the mRNA stability are depicted with dotted-red square. Further, the variant residue is also highlighted with red box, and the entropy values are also mentioned with red colour.

The energy scores obtained for each variant were further analysed for their significance. Out of 73 5'UTR variants, only 8 variants significantly altered the PRKCE mRNA structure. Among these, two variants rs569884823 and rs1227344174 increased the mRNA structural stability, whereas six variants rs946217897, rs1444088897, rs538954895, rs1299335294, rs912480755, and rs1259533133 decreased mRNA stability. Similarly, 10 3'UTR variants out of 17 significantly modified the structural stability of the PRKCE mRNA. Variants rs746238647, rs1333919675, and rs938609813 increased the stability, while variants rs745359100, rs1558956262, rs769370864, rs1321440708, rs1246626878, rs1044257146, and rs1329233194 decreased the stability in comparison to wildtype sequence (Table 3). Further, the structural stability can be further assessed in terms of positional entropy. PRKCE variants that increased the stability of the structure had decreased positional entropy, whereas variants that reduced the structural stability had increased positional entropy (Fig. 6). It is also observed that 5'UTR variant rs1227344174 secondary structure altered to stem loop due to mutation. In comparison to 5'UTR variants, 3'UTR variant more readily caused the removal of stem loop at 3'UTR of PRKCE mRNA. For instance, 3'UTR variants: rs745359100, rs9386089813, rs1329233194, rs1044257146, rs1321440708, and rs1246626878 (Fig. 6). Variant rs918001784 also enhanced the structural stability of the PRKCE mRNA. However, the variant did not have significant impact on the mRNA secondary structure.

**Table 3.** List of PRKCE 5’ and 3’ UTR variants impacting the structural stability of PRKCE mRNA.

5'UTR						3'UTR					
Variant ID	Status	Minimum free energy (Kcal/mol)	RNAstructure Fold	Stability	Significance	Variant ID	Status	Minimum free energy (Kcal/mol)	RNAstructure Fold	Stability	Significance
rs569884823	W	−1.6	−1.5	I	0.001	rs745359100	W	−1	−1.1	D	0.04
	M	−2.5	−2.5				M	0	1		
rs1227344174	W	0	0.1	I	0.001	rs1558956262	W	−2.1	−2.2	D	0.01
	M	−0.8	−0.8				M	−1.9	−1.9		
rs946217897	W	−2.6	−2.5	D	0.0006	rs746238647	W	−3.9	−3.8	I	0.04
	M	−3.9	−3.9				M	−4	−4.0		
rs1444088897	W	−3.7	−4.5	D	0.02	rs769370864	W	−1.6	−1.7	D	0.01
	M	−2.2	−2.2				M	−1.4	−1.4		
rs538954895	W	−5.2	−6.2	D	0.04	rs1321440708	W	−1.4	−1.5	D	0.0005
	M	−3.8	−4.3				M	0	0		
rs1299335294	W	−3.7	−3.6	D	0.0001	rs1333919675	W	−1.8	−1.9	I	0.04
	M	−0.9	−0.9				M	−2	−2.0		
rs912480755	W	−3.5	−3.4	D	0.006	rs1246626878	W	−1.7	−1.6	D	0.0004
	M	−3	−3				M	0	0		
rs1259533133	W	−4.6	−6.9	D	0.04	rs1044257146	W	−1.9	−1.8	D	0.04
	M	−2.4	−1.9				M	−1.7	−1.7		
						rs938609813	W	0	0.1	I	0.001
							M	−1	−1.0		
						rs1329233194	W	−1.4	−1.4	D	0.02
							M	0	0.8		

Abbreviations: W Wild, M Mutated, I Increased, and D Decreased. Significance or P-value is computed through t-test and p-value below 0.05 is taken as significant.

### Influence of PRKCE 5’ and 3’ UTR variants on PRKCE mRNA-miRNA interaction

**Figure 7.**
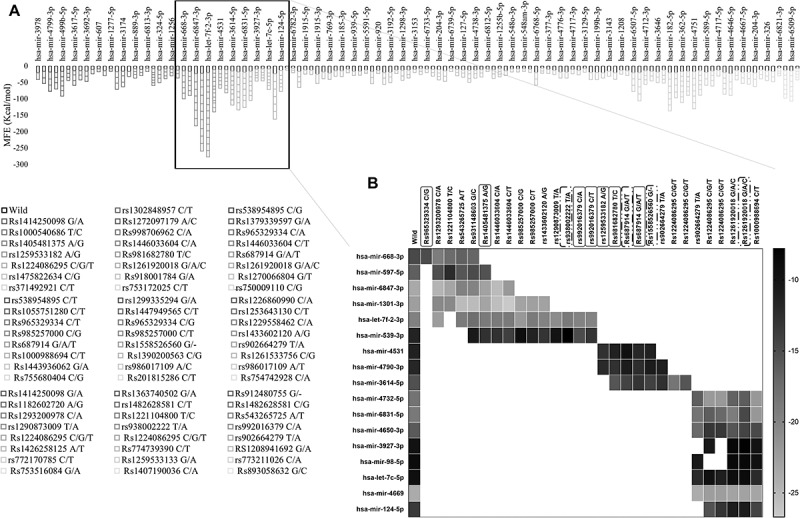
PRKCE gene interaction with miRNAs modulated by its 5'UTR variations. (A) Minimum free energies (MFE) for each PRKCE variation and miRNA interaction were plotted at the y-axis. Around 30 variants were identified that were clustered in miRNA binding sites and greatly affected the miRNA-mRNA interaction. Black outlined boxes indicate wildtype PRKCE-miRNA MFE, whereas different shades of grey represent variant PRKCE. (B) Heatmap for the sorted PRKCE variants vs miRNAs. Darker or near black colour depict less binding affinity while lighter or near white colour boxes represent high binding affinity. Variants that decreased the PRKCE and miRNA interaction are highlighted with grey box, whereas variants that enhanced their interaction are depicted within grey dotted box.

Predicted list of miRNAs that may bind with the 3’ and 5’ UTRs of PRKCE gene was retrieved from miRWalk database. A total of 1109 miRNAs were obtained that may bind with the PRKCE UTRs. Among these, 160 miRNAs have agreement score more than 1 and were selected for further analysis. Binding sites of all these miRNAs were mapped on the 5'UTR of PRKCE gene and 77 5'UTR variants lies in the miRNA binding site on PRKCE gene (Supplementary table 6). Based on minimum free energy value, stable interaction between variant PRKCE mRNA and miRNA in comparison to wild was assessed. Out of 160, 40 miRNAs interaction with PRKCE was not influenced by 5'UTR variants. However, it was observed that miRNAs that bind to the PRKCE 5'UTR at position 45,651,564 to 45,651,644 were more readily influenced by the PRKCE UTR variants. Out of 77, 29 variants lie in this region that either enhance or decrease the binding affinity of 17 miRNAs with PRKCE (Fig. 7a). Enhanced binding affinity of miRNA with PRKCE indicates the expression down-regulation, whereas reduced binding affinity indicates the up-regulation of PRKCE. All the variants (rs1221104800 T/C, rs1293200978 C/G, rs543265725 A/T, rs931148603 G/C, and rs965329334 C/G) in miR-668-3p binding site decreased the binding affinity, whereas the variants (rs931148603 G/C, rs1405481375 A/G, rs1293200978 C/G, and rs1221104800 T/C) in miR-597-5p binding site increased the interaction between miRNA and PRKCE mRNA in comparison to wildtype. In rest of miRNAs’ binding sites, certain mutations promoted affinity while others decreased affinity (Fig. 7b, Table 4). Furthermore, few variants played part solely in decreasing the interaction between miRNAs (whose binding site these variants occurred) and PRKCE mRNA. For instance, rs981682780 T/C, rs1259533182 A/G, Rs965329334 C/G, Rs687914 G/T, Rs1405481375 A/T, rs992016379 C/A, and rs992016379 C/T. Similarly, variants Rs981682780 T/C, rs938002222 T/A, Rs687914 G/A, Rs1558526560 G/-, and Rs1261920018 G/C (Table 4) increased the miRNA and mRNA interaction, that might lead to the down-regulation of PRKCE expression. These variants are also highlighted in Fig. 7b.

**Table 4.** PRKCE 5'UTR variation modulating the 5'UTR interaction with regulatory microRNAs.

	miRNA interaction			miRNA interaction	
Variation	Decreased	Increased	Variation	Decreased	Increased
rs1224086295 C > G	hsa-mir-3614-5p	hsa-mir-6831-5p	rs1224086295 C > T	hsa-mir-3614-5p	hsa-mir-6831-5p
	hsa-mir-4732-5p	hsa-mir-4650-3p		hsa-mir-4732-5p	hsa-mir-4650-3p
	hsa-mir-3927-3p	hsa-mir-98-5p		hsa-let-7c-5p	hsa-mir-98-5p
	hsa-let-7c-5p	hsa-mir-4669			hsa-mir-4669
	hsa-mir-124-5p				hsa-mir-124-5p
rs687914 G > A		hsa-mir-4790-3p	rs687914 G > T	hsa-mir-4790-3p	hsa-mir-3614-5p
		hsa-mir-4531		hsa-mir-4531	
rs981682780 T/C	hsa-mir-4790-3p		rs1259533182 A/G	hsa-mir-4531	
	hsa-mir-3614-5p			hsa-mir-4790-3p	
rs1000988694 C/T	hsa-mir-4732-5p	hsa-mir-4650-3p	rs1261920018 G/C		hsa-mir-4732-5p
	hsa-mir-6831-5p	hsa-mir-4669			hsa-mir-6831-5p
	hsa-mir-3927-3p				hsa-mir-4650-3p
	hsa-mir-98-5p				hsa-mir-3927-3p
	hsa-let-7c-5p				hsa-let-7c-5p
	hsa-mir-124-5p				hsa-mir-4669
rs1290873009 T/A	hsa-let-7 f-2-3p	hsa-mir-539-3p			hsa-mir-124-5p
rs1221104800 T/C	hsa-mir-668-3p	hsa-mir-597-5p	rs1293200978 C/G	hsa-mir-668-3p	hsa-mir-597-5p
		hsa-mir-6847-3p		hsa-mir-6847-3p	hsa-mir-1301-3p
		hsa-mir-1301-3p		hsa-let-7 f-2-3p	
rs1261920018 G/A	hsa-mir-6831-5p	hsa-mir-4732-5p	rs1405481375 A/G	hsa-mir-1301-3p	hsa-mir-597-5p
		hsa-mir-4650-3p			hsa-mir-6847-3p
		hsa-mir-3927-3p			hsa-let-7 f-2-3p
		hsa-let-7c-5p	rs1405481375 A/T	hsa-mir-539-3p	
		hsa-mir-4669	rs1433602120 A/G	hsa-mir-1301-3p	hsa-let-7 f-2-3p
		hsa-mir-124-5p		hsa-mir-539-3p	
rs1446033604 C/A	hsa-mir-6847-3p	hsa-let-7 f-2-3p	rs1558526560 G/-		hsa-mir-4531
	hsa-mir-1301-3p				hsa-mir-4790-3p
	hsa-mir-539-3p				hsa-mir-3614-5p
rs1446033604 C/T	hsa-mir-1301-3p	hsa-mir-6847-3p	rs981682780 T/C		hsa-mir-4531
	hsa-mir-539-3p	hsa-let-7 f-2-3p			
rs543265725 A/T	hsa-mir-668-3p	hsa-mir-6847-3p	rs938002222 T/A		hsa-let-7 f-2-3p
	hsa-mir-1301-3p	hsa-let-7 f-2-3p			hsa-mir-539-3p
		hsa-mir-597-5p	rs965329334 C/G	hsa-mir-668-3p	
rs902664279 T/A	hsa-mir-4650-3p	hsa-mir-4790-3p	rs992016379 C/A	hsa-let-7 f-2-3p	
	hsa-mir-98-5p	hsa-mir-3614-5p		hsa-mir-539-3p	
	hsa-let-7c-5p	hsa-mir-4732-5p	rs992016379 C/T	hsa-let-7 f-2-3p	
	hsa-mir-4669	hsa-mir-6831-5p		hsa-mir-539-3p	
rs931148603 G/C	hsa-mir-668-3p	hsa-mir-597-5p	rs985257000 C/T	hsa-mir-1301-3p	hsa-let-7 f-2-3p
	hsa-mir-1301-3p	hsa-mir-6847-3p			hsa-mir-539-3p
		hsa-let-7 f-2-3p	rs985257000 C/G	hsa-mir-1301-3p	hsa-mir-539-3p
		hsa-mir-539-3p		hsa-let-7 f-2-3p	

### Disease association of PRKCE UTR variants

**Figure 8.**
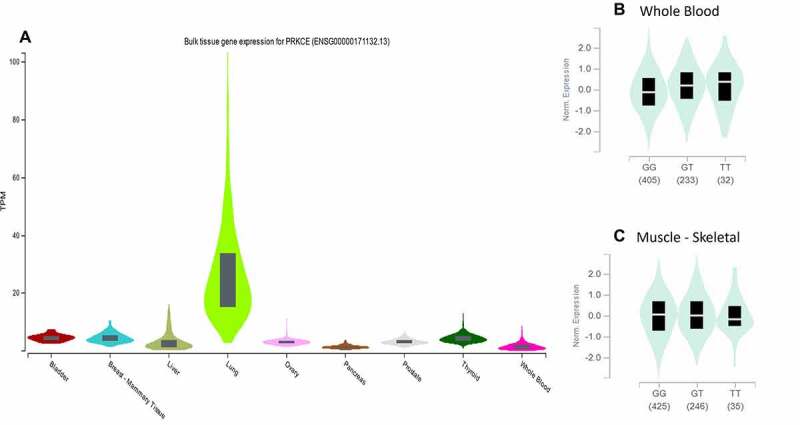
PRKCE gene expression in different tissues. (A) Expression of PRKCE gene is highest in the lungs. (B) Variation rs687014 affects the expression in whole blood ad skeletal muscles.

PRKCE 73 5'UTR and 16 3'UTR variants were further investigated for their association with diseases. Web-based tool ‘rSNP base3.1’ was applied that indicated that out of 73 only one variant rs687914 has association with disease specifically diastolic blood pressure. The further proof of variant rs687914 association with the disease was also found in GWAS catalogue (PMID 28135244). The variant’s influence on the gene expression is depicted in whole blood and skeletal muscles through eQTL analysis with significance of p > 0.00001 and p = 0.00002, respectively. PRKCE differential expression in different tissues and impact of variant rs687914 on PRKCE expression in blood and muscles is shown in Fig. 8.

## Discussion

Evidence has indicated the contribution of non-coding variants in the manifestation of several disorders [58–60]. Deep sequencing approach indicated that non-coding variant disrupts the regulatory motifs at the genes’ transcription sites, causing their aberrant expression [61]. Non-coding variants in the untranslated regions (UTRs) of a gene halts gene expression either at transcription level or at translation level. These variants modify important motifs in the region leading to an altered binding of regulatory proteins to the gene elements [62]. Further, these variants either pre-dispose an individual to a disease or reduces the disease risk [3,63]. Therefore, it is important to understand the disease mechanism at genetic level as well as protein level. Protein Kinase C epsilon (PKCε) is a novel member of PKC family proteins and has been known to play significant role in numerous human diseases [64,65]. Its aberrant expression is particularly reported in cancers such as gall bladder cancer, prostate cancer, brain tumours, and lung cancer [66–70]. Recently, the role of non-synonymous variants in affecting the function and structure of PKCε protein was evaluated through more than thirty bioinformatics tools [17].

The study determined eleven pathogenic variants that effected the activity of PKCε’s kinase or regulatory domains, based on the variant location. As PKCε (gene symbol: PRKCE) expression dysregulation is commonly observed feature in numerous diseases [64,71], the delineation of genetic mechanism behind it will further our knowledge on the mechanism of action of this gene. Therefore, in present study, variants in the UTRs of PRKCE gene were explored to determine their regulatory influence on its expression. The impact of UTR variants on transcription factor binding sites (TFBS) and co-regulatory function of transcription factors (TFs) was explored. Further, UTR variants’ impact on mRNA secondary structure and miRNA binding was also studied. Present research also investigated the potential cellular pathway that participates in the regulation of TFs that are essential for PRKCE gene expression.

UTRs specifically 5'UTR plays significant role on gene transcription. It is located at the 5’ end of protein coding genes and is transcribed into mRNA, but not translated into the protein [72,73]. 5'UTR has role in transcription initiation as it possesses several TFBSs, that assist in the assembly of transcription machinery [72,74]. Similarly, 3'UTR also plays role in the gene expression by allowing binding of TFs, inducing chromatin remodelling, and deciding the fate of the gene [75]. 3'UTR also plays role in transcription termination and newly synthesized mRNA stability [76]. Hence, genetic variability in these regions moulds the TF binding affinity to its binding site on a target gene and leads to aberrant gene expression. In present study, it was necessary to evaluate whether understudied variants have regulatory role or not. Therefore, the PRKCE UTRs’ variant data (Total 376 UTR variants) obtained from SNP databases such as ENSEMBL, COSMIC, EVS, and gnomeAD was first investigated for their regulatory role through web-based server tool, RegulomeDB. The analysis facilitated in identifying a total of 73 5'UTRs and 17 3'UTRs variants that tend to occur at important TF motif site, DNaseI site, or conserved DNA motif site.

Investigation of TFs whose binding affinity was affected by the sorted UTR variants led to the assortment of TFBSs: AP-1, C/EBPdelta, KLF3, c-Fos, c-Jun, c-Mys, CPE binding protein, DI, E1, E2F, GAL4, GATA-1, GR, MIG1, MyoD, and P1 at 5'UTR and TFBSs GR, Ap1, NF-KappaB1, NF-KappaB, ICSBP, and TBP at 3'UTR due to PRKCE UTR variant. Among these, KLF3, MyoD, and FoxP1 act as transcription repressors and rest are transcription activators. AP1 is a family of transcription factors that is subdivided into Fos and Jun subfamilies [77]. PRKCE UTR variants specifically introduce cFos and cJun binding sites at 5'UTR. Further, the activity of TFs in regulating the gene expression is coordinated by more than one TFs. The co-regulatory function of TFs varies from tissue to tissue [75]. In present study, it was found that AP1 interacts with GATA1 and HSF1 in heart as well as bladder and binds with ETF in breast and ovaries. Similarly, E2F binds with AP2, SRF, and E2F in small intestine to induce gene expression. Novel PKC, PKC delta (nPKCε family protein), role in activating cJun and Sp1 in brain is reported. The coordinated activity of cJun and Sp1 protects the brain from inflammation induced injury [78]. It is further found in present study that a positive feedback loop may exist in a cell that allows constitutive expression of PKCε due to UTR variants. The introduction of new TFBSs or multiplication of existing TFBSs such as 1-Oct or Sp1 binding sites may enhance the transcription rate of PRKCE gene [79]. Further, the activity of cJun, cFos, Sp1, cMyc, and NF-κβ is modulated by PKCε [80]; these TFs then go in nucleus and induce the transcription of PRKCE gene. Hence, mutated PKCε gene regulated its own transcription. Studies also indicated that Ap1 regulates the expression of PKC iota in cardiac hypertrophy [81]. This might be the principal mechanism behind the PKCε pathogenicity in different diseases specifically cancers, where its over expression is frequently reported. Presented study provided a significant insight behind the mechanism of pathogenicity of PKCε derived by UTR variants and coordinated by TFs co-activity. In vitro or in vivo experimentations focused in finding the PKCε transcription rate modulation due to UTR or promoter region variant and role of TFs, specifically AP1 and Sp1, in cancers or other metabolic and cardiac disorders might help further our disease knowledge and assist designing therapeutics that are more specific and effective for the disease.

UTRs are not translated into the protein as amino acid sequence; however, they play chief role in mRNA stability, localization, and translation. 5'UTR plays role in ribosome recruitment and translation initiation. The secondary structure of 5'UTR are important in mediating mRNA binding with ribosomes. Complex G-quadruplexes may serve as steric hindrances between mRNA unwinding and translational initiation factors’ ability to scan mRNA [82,83]. 3'UTR, on the other hand, contributes to mRNA stability, localization, and protein translation rate determination. AU-rich 3'UTRs, based on their binding with cis-acting factors or trans-acting factors, contributes to either mRNA decay or fast translation, respectively [84]. Formation of stem loop structures in 5'UTR and 3'UTR also has role in gene expression regulation. Stem loop structures at 5'UTR sterically blocks the access of 43S-preinitiation complex to mRNA and halts translation [82]. In some cases, this phenomenon is utilized by cell to maintain cellular homoeostasis by promoting gene expression at pre-translational level. One of the best studied examples is Iron Response Elements (IRE). Iron regulatory protein 1/2 (IRP1/2) that binds with the IREs of iron transporters when iron is in access and hinders these genes’ translation [85,86]. In present study, PRKCE 5'UTR variant rs569884823 introduced stem loop that may hinder PRKCE mRNA translation. 3'UTR secondary structures also contributes to translational efficacy. Study indicated that circularization of 3'UTR promotes the efficiency of translation. Current study identified four 3'UTR variants: rs745359100, rs746238647, rs1321440708, and rs1044257146, that leads to the formation of circular secondary structure of mRNA. It can be presumed that these 3'UTR variant promotes PRKCE gene translation.

Variants at UTRs also impact the binding affinity of miRNAs to mRNA of the target genes. TargetScan database delineated more than 1000 miRNAs that could potentially bind to PRKCE mRNA. Based on confidence score, 160 miRNAs were sorted and studied for the change in their binding affinity due to UTR variants. The binding sites for these 160 miRNAs were mapped on 5'UTR of PRKCE gene. Most miRNAs bind to the 3'UTR of target mRNA and leads to its translation inhibition or deterioration [87]. Contrarily, miRNAs binding 5'UTR of PRKCE gene may also enhance translational efficiency of the gene [88]. Evidence also indicated that miRNAs can also bind with the secondary structures such as stem loops at 5'UTR and hinder gene transcription [89]. Based on the MFE values, 17 miRNAs were delineated in current study whose binding with PRKCE was influences by 5'UTR variants. Role of these 17 miRNAs in regulating gene expression of PKCε must be explored further through molecular biology experimentation. Further, determining of their role as expression promoter or suppressor for PKCε will be a huge step in designing treatment strategies that will more specifically target PKCε.

In present study, bioinformatics approach was applied to delineate the impact of UTR variants in regulating PKCε gene expression at transcription and translation level. The study outcomes facilitated understanding the potential pathogenic contribution of UTR variants and unravelled the potential molecular mechanism. These outcomes, however, should be validated through in vitro and in vivo experimentation. Further, the disease association of these variants at population level through different genotyping approaches involving high throughput sequencing technology should be done. Current study also determined the role of TFs and their co-regulatory influence in regulating PRKCE gene expression. Co-expression of PRKCE gene with these transcription factors specifically AP1 family TFs should be explored; and through in vitro mutagenesis analysis, impact of UTR variants in promoting or reducing TFs interaction of PRKCE promoter region should be evaluated. Last, UTR variants must be explored in relation with coding region variants, so dual impact of both variant consequences in PKCε function can be evaluated. Insight gained through present study provides a foundation for further experimentations to evaluate the functional consequences of PRKCE UTR variants. These variants could facilitate in determining pre-diagnosis or prognostic genetic marker after further validation and could also facilitate in designing treatment involving PKCε over expression inhibition through miRNAs.

## Supplementary Material

Supplemental Material

## Data Availability

The variant data was retrieved from SNP repositories and can be accessed through (ENSEMBL ensembl.org/; genomeAD gnomad.broadinstitute.org; EVS evs.gs.washington.edu; and COSMIC cancer.sanger.ac.uk). Furthermore, all data supporting the study is available as supplementary files.
