# Supplementary material for: PRKCE non-coding variants influence on transcription as well as translation of its gene
Source: RNA Biol. 2022 Oct 26;19(1):1115–29. doi: 10.1080/15476286.2022.2139110 (PMC9621080; doi:10.1080/15476286.2022.2139110)
Supplement: Supplemental Material [file KRNB_A_2139110_SM6803.zip › ST2.pdf]

Table 2a: List of PRKCE 5'UTRs along with the RegulomeDB score and Rank

Variant ID	Chr: bp	Alleles	RegulomeDB	
			Rank	Score
rs953142436	2:46000904	G/A/C	3a	0.66784
rs953142436	2:46000904	G/A/C	//	//
rs1574178138	2:46000910	G/C	NA	
rs569884823	2:46000911	C/G	3a	0.8507
rs1227344174	2:46000912	T/G	3a	1
rs1222208258	2:46000924	A/-	4	0.60906
rs1287327704	2:46000929	G/C	4	0.60906
rs1354941759	2:46000935	C/T	4	0.60906
rs1288755171	2:46000936	G/A	4	0.60906
rs1238660179	2:46000941	G/A	4	0.60906
rs1335719484	2:46000947	T/C	4	0.60906
rs1302013310	2:46000951	A/G	4	0.60906
rs987625348	2:46000953	G/A	4	0.60906
rs912088552	2:46000957	T/C	4	0.60906
rs1198801585	2:46000959	C/T	4	0.60906
rs1388608323	2:46000966	C/A	4	0.60906
rs1259253443	2:46000967	T/A/G	4	0.60906
rs1259253443	2:46000967	T/A/G	//	//
rs1302848957	2:46000976	C/T	3a	0.98262
rs946217897	2:46000977	C/G/T	3a	0.98262
rs946217897	2:46000977	C/G/T	//	//
rs1180299648	2:46000995	A/G	4	0.60906
rs940538311	2:46001000	C/T	4	0.60906
rs1451472245	2:46001001	A/G	4	0.60906
rs1363166409	2:46001002	G/A	4	0.60906
rs1444088897	2:45651351	G/A	4	0.70497
rs1279310031	2:45651352	C/T	4	0.70497
rs538954895	2:45651354	C/G/T	4	0.70497
rs538954895	2:45651354	C/G/T	//	//
rs1299335294	2:45651355	G/A	4	0.70497
rs1572856920	2:45651356	G/T	NA	NA
rs1228697459	2:45651358	C/T	4	0.60906
rs1363740502	2:45651362	G/A	4	0.70497
rs912480755	2:45651364	G/-	4	0.70497
rs61762789	2:45651364	G/A	4	0.70497
rs1226860990	2:45651365	C/A	4	0.70497
rs558553528	2:45651369	C/G	4	0.70497
rs1414250098	2:45651372	G/A	4	0.70497
rs1297404353	2:45651393	T/C	4	0.60906
rs1572856960	2:45651394	G/T	NA	NA
rs1036898545	2:45651396	C/A	4	0.60906
rs919336515	2:45651401	C/G	4	0.60906
rs929397937	2:45651403	C/G	4	0.60906
rs1558526310	2:45651407	C/T	4	0.60906

rs1203667990	2:45651408	G/T		4	0.60906
rs1184836192	2:45651409	C/T		4	0.60906
rs1049077537	2:45651410	C/A/T		4	0.60906
rs1049077537	2:45651410	C/A/T	//	//	
rs1209401734	2:45651412	G/C		4	0.60906
rs887745328	2:45651413	C/T		4	0.60906
rs113923860	2:45651417	G/A	2b		0.49614
rs1276515174	2:45651423	G/A	2b		0.63769
rs148164101	2:45651426	C/T		4	0.60906
rs1341045869	2:45651432	C/T	2c		0.238
rs1272097179	2:45651437	A/C	2c		0.715
rs1229534513	2:45651439	G/A	2b		0.445
rs1379339597	2:45651444	G/A	2b		0.84
rs1055751280	2:45651452	C/T	2c		0.98
rs1447949565	2:45651455	C/T	2c		0.76
rs1353913478	2:45651461	G/A	2c		0.238
rs1310267095	2:45651471	T/C		4	0.60906
rs776148119	2:45651472	G/C		4	0.60906
rs1370156637	2:45651483	A/C		4	0.60906
rs896768686	2:45651486	T/C		4	0.60906
rs1407140099	2:45651489	G/A		4	0.60906
rs1414640473	2:45651490	C/T		4	0.60906
rs537436368	2:45651494	G/T		4	0.60906
rs1558526386	2:45651500	G/T	2b		0.31692
rs1203735091	2:45651501	A/G	2b		0.38632
rs1253643130	2:45651503	C/T	2b		0.8368
rs1182602720	2:45651504	A/G	2b		0.8368
rs1482628581	2:45651509	C/G/T	2b		0.86817
rs1482628581	2:45651509	C/G/T	//	//	
rs61762790	2:45651514	G/A		4	0.60906
rs1321725839	2:45651517	A/C		4	0.60906
rs1288565577	2:45651518	T/-		4	0.60906
rs888821099	2:45651519	G/A/C		4	0.60906
rs888821099	2:45651519	G/A/C	//	//	
rs1333311600	2:45651521	A/T		4	0.60906
rs1008778423	2:45651523	C/T		4	0.60906
rs1018883365	2:45651528	C/G/T		4	0.60906
rs1018883365	2:45651528	C/G/T	//	//	
rs574540048	2:45651530	G/C		4	0.60906
rs1187931975	2:45651535	A/G		4	0.60906
rs1422033733	2:45651537	T/C		4	0.60906
rs1558526423	2:45651541	C/T		4	0.60906
rs1000540686	2:45651547	T/C		4	0.70497
rs1350500051	2:45651549	C/G		4	0.70497
rs998706962	2:45651553	C/A	2b		1
rs953800382	2:45651555-45651561	CCCCCCC/CCCCCC/CCCCCCCC	//	//	
rs953800382	2:45651555-45651561	CCCCCCC/CCCCCC/CCCCCCCC	2b		0.604

rs1572857178	2:45651557	C/A	NA	NA	
rs1159895838	2:45651558	C/T	2b		0.64862
rs1032869877	2:45651559	C/A/T	2b		0.64862
rs1032869877	2:45651559	C/A/T	//	//	
rs965329334	2:45651560	C/A/G/T	2b		0.77392
rs965329334	2:45651560	C/A/G/T	//	//	
rs965329334	2:45651560	C/A/G/T	//	//	
rs972544438	2:45651561	C/A/G/T	2b		0.67017
rs972544438	2:45651561	C/A/G/T	//	//	
rs972544438	2:45651561	C/A/G/T	//	//	
rs1229558462	2:45651565	C/A	2b		0.70883
rs1316628781	2:45651566	C/A	2b		0.58489
rs918452630	2:45651568	C/A	2b		0.6749
rs1457185129	2:45651571-45651572	GG/G	2b		0.6749
rs1221104800	2:45651573	T/A/C		4	0.70497
rs1221104800	2:45651573	T/A/C	//	//	
rs1293200978	2:45651574	C/G		4	0.70497
rs543265725	2:45651575	A/T		4	0.70497
rs931148603	2:45651576	G/C		4	0.70497
rs1405481375	2:45651581	A/G		4	0.70497
rs1446033604	2:45651584	C/A/T		4	0.70497
rs1446033604	2:45651584	C/A/T	//	//	
rs985257000	2:45651586	C/G/T		4	0.70497
rs985257000	2:45651586	C/G/T	//	//	
rs1290873009	2:45651587	T/A		4	0.70497
rs1433602120	2:45651589	A/G		4	0.70497
rs938002222	2:45651591	T/A		4	0.70497
rs992016379	2:45651595	C/A/T		4	0.70497
rs992016379	2:45651595	C/A/T	//	//	
rs1465811840	2:45651596	G/T		4	0.70497
rs915909888	2:45651607	G/A/T		4	0.60906
rs915909888	2:45651607	G/A/T	//	//	
rs1213772661	2:45651608	G/C		4	0.70497
rs971806465	2:45651609	G/A		4	0.70497
rs1259533182	2:45651615	A/G		4	0.70497
rs981682780	2:45651618	T/C		4	0.70497
rs687914	2:45651621	G/A/T		4	0.70497
rs687914	2:45651621	G/A/T	//	//	
rs1558526560	2:45651621	G/-		4	0.70497
rs1558526579	2:45651622	C/T		4	0.60906
rs1558526584	2:45651625	C/G		4	0.60906
rs1302882757	2:45651626	C/T		4	0.60906
rs902664279	2:45651630	T/A		4	0.70497
rs1224086295	2:45651633	C/G/T	2b		0.75124
rs1224086295	2:45651633	C/G/T	//	//	
rs1261920018	2:45651640	G/A/C	2b		1
rs1261920018	2:45651640	G/A/C	//	//	

rs1000988694	2:45651642	C/T	2b		0.86817
rs61762791	2:45651644	C/A	2b		0.58489
rs1217420340	2:45651645	G/A/C	2b		0.79558
rs1217420340	2:45651645	G/A/C	//	//	
rs1242340436	2:45651650	A/C	2b		0.48203
rs1485377731	2:45651653	G/C	2b		0.2664
rs1193370050	2:45651655	C/A	2b		0.50489
rs1171280885	2:45651658	A/C	2b		0.67419
rs1572857394	2:45651659	T/C	NA	NA	
rs1450974059	2:45651665	T/C	2b		0.60823
rs1390200563	2:45651668	C/G/T		4	0.70497
rs1390200563	2:45651668	C/G/T	//	//	
rs1572857415	2:45651671	A/G	NA	NA	
rs1572857417	2:45651673	T/G	NA	NA	
rs1426258125	2:45651678	A/T		4	0.70497
rs1572857429	2:45651679	T/A	NA	NA	
rs1261533756	2:45651682	C/G	2b		0.81166
rs1260255256	2:45651685	G/T	2b		0.68851
rs1572857439	2:45651688	A/G	NA	NA	
rs1184169589	2:45651691	A/G	2b		0.63605
rs1483745630	2:45651693	G/A	2b		0.44059
rs774739390	2:45651697	C/T	2b		1
rs1208941692	2:45651698	G/A	2b		0.85691
rs1572857461	2:45651700	A/G	NA	NA	
rs1344558376	2:45651703	G/A	2b		0.84289
rs369165780	2:45651904	G/A		4	0.60906
rs1237434490	2:45651907	C/T		4	0.60906
rs532206195	2:45651909	C/T		4	0.60906
rs185312969	2:45651911	G/A		4	0.60906
rs952646099	2:45651912	C/T		4	0.60906
rs1299198258	2:45651913	G/A		4	0.60906
rs1223675490	2:45651916	G/A		4	0.60906
rs144145681	2:45651923	A/G/T		4	0.60906
rs144145681	2:45651923	A/G/T	//	//	
rs1450358402	2:45651924	T/C		4	0.60906
rs1399307545	2:45651926	C/T		4	0.60906
rs1207275949	2:45651931	G/T		4	0.60906
rs958797977	2:45651934	G/A		4	0.60906
rs998767594	2:45651936	G/C		4	0.60906
rs1054376112	2:45651940	C/T		4	0.60906
rs537845343	2:45651941	G/T		4	0.60906
rs1558526980	2:45651944	C/A/G		4	0.60906
rs1558526980	2:45651944	C/A/G	//	//	
rs892933923	2:45651947	G/A		4	0.60906
rs1013176378	2:45651950	A/G		4	0.60906
rs554579350	2:45651952	G/A/T		4	0.60906
rs554579350	2:45651952	G/A/T	//	//	

rs1023026345	2:45651955	G/A		4	0.60906
rs1475822634	2:45651956	C/G		4	0.70497
rs918001784	2:45651960	G/A/T		4	0.70497
rs918001784	2:45651960	G/A/T	//	//	
rs1443866009	2:45651963	A/T		4	0.60906
rs11889760	2:45651967	G/A		4	0.60906
rs1002940624	2:45651970	G/A	2b		0.64343
rs1345261565	2:45651976	A/T	2b		0.41199
rs79452669	2:45651980	T/A	2b		0.34664
rs961363156	2:45651983	G/A/C	2b		0.58489
rs961363156	2:45651983	G/A/C	//	//	
rs1270066804	2:45651986	G/T	2b		0.86817
rs1572857946	2:45651987	T/G	NA	NA	
rs553881156	2:45651991	G/C	2b		0.59816
rs1429241818	2:45651992	C/T	2b		0.40652
rs1342595564	2:45651994	A/C	2b		0.38632
rs1388350686	2:45651998	G/A	2b		0.66759
rs1414596511	2:45651999	G/A/T	2b		0.66759
rs1414596511	2:45651999	G/A/T	//	//	
rs1572857967	2:45652000	G/A	NA	NA	
rs1170782953	2:45652003	C/G		4	0.60906
rs974262371	2:45652011	C/A		4	0.60906
rs1458564640	2:45652013	C/T		4	0.60906
rs1329471664	2:45652016	T/A		4	0.60906
rs1027663004	2:45652021	C/T		4	0.60906
rs983363042	2:45652022	G/C		4	0.60906
rs954214615	2:45652028	C/T		4	0.60906
rs924195512	2:45652030	G/A/C		4	0.60906
rs924195512	2:45652030	G/A/C		4	0.60906
rs1418905014	2:45652030-45652032	GGG/GGGG		4	0.60906
rs1443936062	2:45652035	G/A	2b		0.83617
rs1572858030	2:45652040	T/G	NA	NA	
rs1258191129	2:45652044	T/A	2b		0.54543
rs986017109	2:45652046	A/C	2b		0.85691
rs1186915888	2:45652047	T/A	2b		0.54543
rs772170785	2:45652050	C/T	2b		0.73193
rs1259533133	2:45652052	G/A	2b		0.82852
rs773211026	2:45652054	C/A/T	2b		0.7614
rs773211026	2:45652054	C/A/T	//	//	
rs1205442794	2:45652057	C/T	2b		0.56046
rs1167247806	2:45652057	C/-	2b		0.46658
rs573424161	2:45652058	G/C	2b		0.46658
rs1558527129	2:45652064	G/A		4	0.60906
rs372046784	2:45652067	G/A		4	0.60906
rs771203665	2:45652071	T/G		4	0.60906
rs911571016	2:45652073	A/C		4	0.60906
rs12615066	2:45652074	C/A/T	//	//	

rs12615066	2:45652074	C/A/T		4	0.60906
rs1359042538	2:45652076	C/T		4	0.60906
rs200186038	2:45652077	C/G/T		4	0.60906
rs200186038	2:45652077	C/G/T	//	//	
rs766342177	2:45652080-45652084	CCCCC/CCCCCC		4	0.60906
rs753172025	2:45652082	C/T	2b		0.8153
rs371492921	2:45652083	C/T	2b		0.8288
rs934320928	2:45652084	C/G	2b		0.69075
rs767145681	2:45652085	A/C/G	2b		0.64249
rs767145681	2:45652085	A/C/G	//	//	
rs750009110	2:45652088	C/G	2b		0.76029
rs755680404	2:45652090	C/G	2b		0.70883
rs201815286	2:45652091	C/T	2b		0.70883
rs753516084	2:45652092	G/A	2b		1
rs1407190036	2:45652093	C/A	2b		0.86817
rs754742928	2:45652094	C/A	2b		0.86817
rs900931286	2:45652095	C/T	2b		0.70883
rs893058632	2:45652097	G/A/C	2b		1
rs893058632	2:45652097	G/A/C	//	//	
rs1485871449	2:45652100	C/T	3a		0.50689

Table 2b: List of PRKCE 3'UTRs along with the RegulomeDB score and Rank

Variant ID	Chr: bp	Alleles	Rank	RegulomeDB Score	
rs938015012	2:46010742	C/A		**4**	0.60906
rs1055560049	2:46010747	C/T		4	0.60906
rs372769511	2:46010749	G/A		4	0.60906
rs1475690510	2:46010750	C/A		4	0.60906
rs1254183218	2:46010751	C/T		4	0.60906
rs755354116	2:46010752	A/T		4	0.60906
rs779297740	2:46010755	G/A		4	0.60906
rs748583576	2:46010761	C/A/G		4	0.60906
rs748583576	2:46010761	C/A/G	//	//	
rs756787103	2:46010766	C/G/T		4	0.60906
rs756787103	2:46010766	C/G/T	//	//	
rs1407342066	2:46010767	C/T		4	0.60906
rs886936491	2:46010774	C/G		4	0.60906
rs1381946616	2:46010775	T/C		4	0.60906
rs745359100	2:46010779	C/T	2c		0.74633
rs1558956262	2:46010780	T/A	2c		0.92
rs746238647	2:46010781	C/-	2c		0.92
rs746238647		c.*43T>A	//	//	
rs1328765904	2:46010781	C/T	2c		0.21
rs772386702	2:46010784-46010789	TGTTTG/TG	2a		0.25174
rs769370864	2:46010784	T/G	2a		1
rs1321440708	2:46010785	G/A	2a		0.72927
rs975614092	2:46010790	C/G	2a		0.68331
rs1328180994	2:46010792	C/T	2a		0.50017
rs1558956323	2:46010798	A/T	2b		0.64083
rs1333919675	2:46010802	A/G	2b		0.71276
rs761990090		c.*3G>A		5	0.12326
rs772155036		c.*6C>G		5	0.3715
rs571801707		c.*11C>T		5	0.9223
rs761011720		c.*13G>T		5	0
rs376002289		c.*17G>C		5	0.15146
rs777043604		c.*19C>G		5	0.71614
rs571539218		c.*25C>T		5	0.20707
rs765743344		c.*26G>A		5	0
rs1322443197		c.*35A>G		5	0.13454
rs940477851		c.*42G>A		5	0.13454
rs537772053		c.*44G>T		5	0.13454
rs1314269254		c.*46A>G		5	0.13454
rs557556854		c.*48A>G		5	0.13454
rs767003027		c.*51A>G		5	0.13454
rs749879567		c.*52G>C		5	0.13454
rs1028962764	2:46010804-46010809	CAACAA/CAA	2b		0.57087
rs1323365703	2:46010804-46010805	CA/-	2b		0.57087
rs1246626878	2:46010804	C/T	3a		0.80781

rs1004083544	2:46010811	T/C/G	3a		0.99633
rs1004083544	2:46010811	T/C/G	//	//	
rs1284594751	2:46010815	G/A		4	0.60906
rs377361438	2:46010819	G/A		4	0.60906
rs962973678	2:46010821	C/G		4	0.60906
rs751131166	2:46010825	G/A		4	0.60906
rs377498969	2:46010832	T/C		4	0.60906
rs1276115928	2:46010834-46010839	CTAACT/CT		4	0.60906
rs61758295	2:46010838	C/T		4	0.60906
rs756914020	2:46010846	C/T		4	0.60906
rs1483134616	2:46010858	C/A	2b		0.40652
rs908576118	2:46010860	C/T	2b		0.40652
rs1028379694	2:46010874	T/C	2b		0.8662
rs974526523	2:46010876	T/C	2b		0.8662
rs376230022	2:46010880	C/T	2b		0.194
rs948532411	2:46010883	G/A	3a		0.60177
rs1180275126	2:46010885	C/G	3a		0.44955
rs1044257146	2:46010888	A/G	3a		0.72733
rs986474131	2:46010891	A/T	3a		0.38328
rs938609813	2:46010892	A/G/T	2a		0.80833
rs938609813	2:46010892	A/G/T	//	//	
rs913693685	2:46010894	A/G/T	2a		0.41225
rs913693685	2:46010894	A/G/T	//	//	
rs1244029391	2:46010896	G/A	2a		0.47578
rs1238436678	2:46010900-46010902	AAA/AA	2a		1
rs1329233194	2:46010904	G/T	2a		1
rs1289740339	2:46010908	C/G/T	2a		0.48344
rs1289740339	2:46010908	C/G/T	//	//	
rs531634050	2:46010916	T/C		4	0.60906
rs548459552	2:46010922	A/G		4	0.60906
rs184464504	2:46010923	A/T		4	0.60906
rs34665948	2: between 46010924 & 46	-/A		4	0.60906
rs1392099843	2:46010934	G/C		4	0.60906
rs1388838826	2:46010937	A/T		4	0.60906
rs1389085829	2:46010950	G/A		4	0.60906
rs527440597	2:46010958	C/T		4	0.60906
rs1293423618	2:46010959	A/G		4	0.60906
rs547615242	2:46010962	T/G		4	0.60906
