# Supplementary material for: PRKCE non-coding variants influence on transcription as well as translation of its gene
Source: RNA Biol. 2022 Oct 26;19(1):1115–29. doi: 10.1080/15476286.2022.2139110 (PMC9621080; doi:10.1080/15476286.2022.2139110)
Supplement: Supplemental Material [file KRNB_A_2139110_SM6803.zip › ST4.pdf]

Table 4a: Impact of PRKCE 5'UTR variants on the transcription factor binding sites

Variant ID	Allele type	Chr: bp	Alleles	TF-BS	Class	Trancription factor	Start	Stop
rs569884823	Wild	2:46000911	C	3	1.1.3.0	C/EBPalpha	9	18
					2.1.1.1	GR	18	27
					3.6.1.0	TEC1	42	51
	Mutated		G	3	1.1.3.0	C/EBPalpha	9	18
					2.1.1.1	GR	18	27
					3.6.1.0	TEC1	42	51
rs1227344174	Wild	2:46000912	T	3	1.1.3.0	C/EBPalpha	9	18
					2.1.1.1	GR	18	27
					3.6.1.0	TEC1	42	51
	Mutated		G	3	1.1.3.0	C/EBPalpha	10	19
					2.1.1.1	GR	18	27
					3.6.1.0	TEC1	42	51
rs1302848957	WILD	2:46000976	C	7	3.1.2.2	Oct-01	27	36
					2.1.1.4	ER	44	53
					1.1.1.1	c-Jun	49	58
					1.1.1.6	CRE-BP1	49	58
					2.1.1.1	GR	49	58
					1.1.2.0	CREB	50	59
					2.3.3.0	CPE_binding_pro	52	61
	Mutated		T	7	3.1.2.2	Oct-01	27	36
					2.1.1.4	ER	44	53
					1.1.1.1	c-Jun	49	58
					1.1.1.6	CRE-BP1	49	58
					2.1.1.1	GR	49	58
					1.1.2.0	CREB	50	59
					2.3.3.0	CPE_binding_pro	52	61
rs946217897	Wild	2:46000977	C	7	3.1.2.2	Oct-01	27	36
					2.1.1.4	ER	44	53
					1.1.1.1	c-Jun	49	58
					1.1.1.6	CRE-BP1	49	58
					2.1.1.1	GR	49	58
					1.1.2.0	CREB	50	59
					2.3.3.0	CPE_binding_pro	52	61
	Mutated		G	7	3.1.2.2	Oct-01	27	36
					2.1.1.4	ER	44	53
					1.1.1.1	c-Jun	49	58
					1.1.1.6	CRE-BP1	49	58
					2.1.1.1	GR	49	58
					1.1.2.0	CREB	50	59
					2.3.3.0	CPE_binding_pro	52	61
	Mutated		T	7	3.1.2.2	Oct-01	27	36
					2.1.1.4	ER	44	53
					1.1.1.1	c-Jun	49	58
					1.1.1.6	CRE-BP1	49	58
					2.1.1.1	GR	49	58
					1.1.2.0	CREB	50	59

					2.3.3.0	CPE_binding_pro	52	61
					2.3.1.0	Sp1	15	26
					2.3.2.3	WT1	16	25
	Wild		G	5	1.6.1.0	AP-2	41	50
					2.3.1.0	Sp1	44	53
					2.3.1.0	Sp1	50	59
rs1444088897		2:45651351			2.3.1.0	Sp1	15	26
					2.3.2.3	WT1	16	25
	Mutated		A	5	1.6.1.0	AP-2	41	50
					2.3.1.0	Sp1	44	53
					2.3.1.0	Sp1	50	59
					2.3.1.0	Sp1	15	26
					2.3.2.3	WT1	16	25
	Wild	2:45651352	C	5	1.6.1.0	AP-2	41	50
					2.3.1.0	Sp1	44	53
					2.3.1.0	Sp1	50	59
rs1279310031					2.3.1.0	Sp1	15	24
					2.3.2.3	WT1	16	25
	Mutated	2:45651352	T	5	1.6.1.0	AP-2	41	50
					2.3.1.0	Sp1	44	53
					2.3.1.0	Sp1	50	59
					2.3.1.0	Sp1	15	26
					2.3.2.3	WT1	16	25
	Wild		C	5	1.6.1.0	AP-2	41	50
					2.3.1.0	Sp1	44	53
					2.3.1.0	Sp1	50	59
					2.3.1.0	Sp1	15	26
					2.3.2.3	WT1	16	25
rs538954895	Mutated	2:45651354	G	5	1.6.1.0	AP-2	41	50
					2.3.1.0	Sp1	44	53
					2.3.1.0	Sp1	50	59
					2.3.1.0	Sp1	8	17
					2.3.1.0	Sp1	15	26
	Mutated		T	6	2.3.2.3	WT1	16	25
					1.6.1.0	AP-2	41	50
					2.3.1.0	Sp1	44	53
					2.3.1.0	Sp1	50	59
					2.3.1.0	Sp1	15	26
					2.3.2.3	WT1	16	25
	Wild		G	5	1.6.1.0	AP-2	41	50
					2.3.1.0	Sp1	44	53
					2.3.1.0	Sp1	50	59
rs1299335294		2:45651355			2.3.1.0	Sp1	15	26
					2.3.2.3	WT1	16	25
	Mutated		A	5	1.6.1.0	AP-2	41	50
					2.3.1.0	Sp1	44	53
					2.3.1.0	Sp1	50	59
					2.3.1.0	Sp1	15	26
					2.3.2.3	WT1	16	25

	Wild		G	5	1.6.1.0	AP-2	41	50
rs1363740502		2:45651362			2.3.1.0	Sp1	44	53
					2.3.1.0	Sp1	50	59
					1.6.1.0	AP-2	41	50
	Mutated		A	3	2.3.1.0	Sp1	44	53
					2.3.1.0	Sp1	50	59
					2.3.1.0	Sp1	15	26
					2.3.2.3	WT1	16	25
	Wild		G	5	1.6.1.0	AP-2	41	50
					2.3.1.0	Sp1	44	53
rs912480755		2:45651364			2.3.1.0	Sp1	50	59
					2.3.1.0	Sp1	16	25
	Mutated		--	4	1.6.1.0	AP-2	40	49
					2.3.1.0	Sp1	43	52
					2.3.1.0	Sp1	49	58
					2.3.1.0	Sp1	15	26
					2.3.2.3	WT1	16	25
	Wild		G	5	1.6.1.0	AP-2	41	50
rs61762789		2:45651364			2.3.1.0	Sp1	44	53
					2.3.1.0	Sp1	50	59
					1.6.1.0	AP-2	41	50
	Mutated		A	3	2.3.1.0	Sp1	44	53
					2.3.1.0	Sp1	50	59
					2.3.1.0	Sp1	15	26
					2.3.2.3	WT1	16	25
	Wild		C	5	1.6.1.0	AP-2	41	50
					2.3.1.0	Sp1	44	53
rs1226860990		2:45651365			2.3.1.0	Sp1	50	59
					2.3.1.0	Sp1	15	24
	Mutated		A	4	1.6.1.0	AP-2	41	50
					2.3.1.0	Sp1	44	53
					2.3.1.0	Sp1	50	59
					2.3.1.0	Sp1	15	26
					2.3.2.3	WT1	16	25
	Wild		C	5	1.6.1.0	AP-2	41	50
					2.3.1.0	Sp1	44	53
rs558553528		2:45651369			2.3.1.0	Sp1	50	59
					2.3.1.0	Sp1	17	31
	Mutated		G	4	1.6.1.0	AP-2	41	50
					2.3.1.0	Sp1	44	53
					2.3.1.0	Sp1	50	59
					2.3.1.0	Sp1	15	26
					2.3.2.3	WT1	16	25
	Wild		G	5	1.6.1.0	AP-2	41	50
					2.3.1.0	Sp1	44	53
					2.3.1.0	Sp1	50	59
rs1414250098		2:45651372			2.3.1.0	Sp1	15	26
					2.3.2.3	WT1	16	25
					9.9.29	AP-1	25	34
	Mutated		A	6				

	Mutated		A	[illegible]	1.6.1.0	AP-2	41	50
					2.3.1.0	Sp1	44	53
					2.3.1.0	Sp1	50	59
					2.3.1.0	Sp1	5	14
					9.9.637	NRF-1	8	17
					9.9.1197	NRF-1	8	17
					2.3.1.0	Sp1	12	21
					2.3.1.0	Sp1	23	33
	Wild		A	11	2.3.1.0	Sp1	30	42
					2.3.2.1	Krox-20	33	42
					2.3.1.0	Sp1	36	49
					2.3.1.0	Sp1	42	51
					2.3.1.0	Sp1	48	57
rs1272097179		2:45651437			9.9.539	NF-1	48	57
					2.3.1.0	Sp1	5	14
					9.9.637	NRF-1	8	17
					9.9.1197	NRF-1	8	17
					2.3.1.0	Sp1	12	21
					2.3.1.0	Sp1	23	33
	Mutated		C	11	2.3.1.0	Sp1	30	43
					2.3.2.1	Krox-20	34	43
					2.3.1.0	Sp1	36	48
					2.3.1.0	Sp1	42	52
					2.3.1.0	Sp1	48	57
					9.9.539	NF-1	48	57
					2.3.1.0	Sp1	5	14
					9.9.637	NRF-1	8	17
					9.9.1197	NRF-1	8	17
					2.3.1.0	Sp1	12	21
					2.3.1.0	Sp1	23	33
	Wild		G	11	2.3.1.0	Sp1	30	42
					2.3.2.1	Krox-20	33	42
					2.3.1.0	Sp1	36	49
					2.3.1.0	Sp1	42	51
rs1379339597		2:45651444			2.3.1.0	Sp1	48	57
					9.9.539	NF-1	48	57
					2.3.1.0	Sp1	5	14
					9.9.637	NRF-1	8	17
					9.9.1197	NRF-1	8	17
					2.3.1.0	Sp1	12	21
	Mutated		A	9	2.3.1.0	Sp1	27	36
					2.3.1.0	Sp1	34	48
					2.3.1.0	Sp1	42	52
					2.3.1.0	Sp1	48	57
					9.9.539	NF-1	48	57
					2.3.1.0	Sp1	5	14
					9.9.637	NRF-1	8	17
					9.9.1197	NRF-1	8	17
					2.3.1.0	Sp1	12	21

					2.3.1.0	Sp1	23	33
	Wild		C	11	2.3.1.0	Sp1	30	42
					2.3.2.1	Krox-20	33	42
					2.3.1.0	Sp1	36	49
					2.3.1.0	Sp1	42	51
					2.3.1.0	Sp1	48	57
rs1055751280		2:45651452			9.9.539	NF-1	48	57
					2.3.1.0	Sp1	5	14
					9.9.637	NRF-1	8	17
					9.9.1197	NRF-1	8	17
					2.3.1.0	Sp1	12	21
	Mutated		T	10	2.3.1.0	Sp1	23	33
					2.3.1.0	Sp1	30	43
					2.3.2.1	Krox-20	33	42
					2.3.1.0	Sp1	41	50
					2.3.1.0	Sp1	48	57
					9.9.539	NF-1	48	57
					2.3.1.0	Sp1	5	14
					9.9.637	NRF-1	8	17
					9.9.1197	NRF-1	8	17
					2.3.1.0	Sp1	12	21
					2.3.1.0	Sp1	23	33
	WILD		C	11	2.3.1.0	Sp1	30	42
					2.3.2.1	Krox-20	33	42
					2.3.1.0	Sp1	36	49
					2.3.1.0	Sp1	42	51
					2.3.1.0	Sp1	48	57
rs1447949565		2:45651455			9.9.539	NF-1	48	57
					2.3.1.0	Sp1	5	14
					9.9.637	NRF-1	8	17
					9.9.1197	NRF-1	8	17
					2.3.1.0	Sp1	12	21
	Mutated		T	11	2.3.1.0	Sp1	23	33
					2.3.1.0	Sp1	30	42
					2.3.2.1	Krox-20	33	42
					2.3.1.0	Sp1	36	48
					9.9.537	NF-1	50	59
					9.9.539	NF-1	50	59
					1.1.3.0	C/EBPalpha	8	17
					2.3.1.0	Sp1	21	30
	WILD			6	9.9.539	NF-1	21	30
					1.1.3.0	C/EBPalpha	36	45
					2.3.1.0	Sp1	36	45
rs1253643130		2:45651503			9.9.590	NF-kappaB	38	47
					1.1.3.0	C/EBPalpha	8	17
					2.3.1.0	Sp1	21	30
			C/T	6	9.9.539	NF-1	21	30
					1.1.3.0	C/EBPalpha	36	45
					2.3.1.0	Sp1	36	45

	WILD		A	6	4.1.1.0	NF-kappaB2_(p49	38	47
					1.1.3.0	C/EBPalpha	8	17
					2.3.1.0	Sp1	21	30
					9.9.539	NF-1	21	30
					1.1.3.0	C/EBPalpha	36	45
					2.3.1.0	Sp1	36	45
rs1182602720		2:45651504			9.9.590	NF-kappaB	38	47
	Mutated		G	4	1.1.3.0	C/EBPalpha	8	17
					2.3.1.0	Sp1	21	30
					9.9.539	NF-1	21	30
					2.4.1.0	GAL4	31	40
	WILD		C	6	1.1.3.0	C/EBPalpha	8	17
					2.3.1.0	Sp1	21	30
					9.9.539	NF-1	21	30
					1.1.3.0	C/EBPalpha	36	45
					2.3.1.0	Sp1	36	45
					9.9.590	NF-kappaB	38	47
rs1482628581		2:45651509	G	4	1.1.3.0	C/EBPalpha	8	17
					2.3.1.0	Sp1	21	30
					9.9.539	NF-1	21	30
					1.1.3.0	C/EBPalpha	36	45
			T	4	1.1.3.0	C/EBPalpha	8	17
					2.3.1.0	Sp1	21	30
					9.9.539	NF-1	21	30
					1.1.3.0	C/EBPdelta	36	45
	WILD		T	8	2.3.1.0	Sp1	15	24
					2.3.1.0	Sp1	30	44
					2.3.2.1	Egr-1	32	41
					2.3.2.3	GLI3	32	41
					9.9.270	ETF	32	41
					1.6.1.0	AP-2alphaA	36	45
					2.3.1.0	Sp1	36	45
					2.3.1.0	Sp1	49	59
rs1000540686		2:45651547						
	Mutated		C	8	2.3.1.0	Sp1	19	28
					2.3.1.0	Sp1	25	39
					2.3.2.1	Egr-1	32	41
					2.3.2.3	GLI3	32	41
					9.9.270	ETF	32	41
					2.3.1.0	Sp1	32	46
					1.6.1.0	AP-2alphaA	36	45
					2.3.1.0	Sp1	49	59
	WILD		C	8	2.3.1.0	Sp1	15	24
					2.3.1.0	Sp1	30	44
					2.3.2.1	Egr-1	32	41
					2.3.2.3	GLI3	32	41
					9.9.270	ETF	32	41
					1.6.1.0	AP-2alphaA	36	45
					2.3.1.0	Sp1	36	45
rs1350500051		2:45651549			2.3.1.0	Sp1	49	59

					2.3.1.0	Sp1	30	44
					2.3.2.1	Egr-1	32	41
					2.3.2.3	GLI3	32	41
	Mutated		G	7	9.9.270	ETF	32	41
					1.6.1.0	AP-2alphaA	36	45
					2.3.1.0	Sp1	36	45
					2.3.1.0	Sp1	49	59
					2.3.1.0	Sp1	15	24
					2.3.1.0	Sp1	30	44
					2.3.2.1	Egr-1	32	41
	WILD		C	8	2.3.2.3	GLI3	32	41
					9.9.270	ETF	32	41
					1.6.1.0	AP-2alphaA	36	45
					2.3.1.0	Sp1	36	45
					2.3.1.0	Sp1	49	59
rs998706962		2:45651553			2.3.1.0	Sp1	15	24
					2.1.2.1	RAR-alpha1	23	32
					2.3.1.0	Sp1	30	44
					2.3.2.1	Egr-1	32	41
	Mutated		A	9	2.3.2.3	GLI3	32	41
					9.9.270	ETF	32	41
					1.6.1.0	AP-2alphaA	36	45
					2.3.1.0	Sp1	36	45
					2.3.1.0	Sp1	49	59
					2.3.1.0	Sp1	15	24
					2.3.1.0	Sp1	30	44
					2.3.2.1	Egr-1	32	41
	WILD		C	8	2.3.2.3	GLI3	32	41
					9.9.270	ETF	32	41
					1.6.1.0	AP-2alphaA	36	45
					2.3.1.0	Sp1	36	45
					2.3.1.0	Sp1	49	59
					2.3.1.0	Sp1	15	24
					2.3.1.0	Sp1	27	41
					9.9.270	ETF	31	40
	Mutated		A	7	2.3.2.1	Egr-1	32	41
					2.3.1.0	Sp1	33	47
					1.6.1.0	AP-2alphaA	36	45
					2.3.1.0	Sp1	49	59
rs965329334		2:45651560			.3.1.0 S	p1 1	5	24
					2.3.1.0	Sp1	27	41
					2.3.2.1	Egr-1	30	39
	Mutated		T	8	2.3.2.3	WT1	32	41
					2.3.1.0	Sp1	33	46
					1.6.1.0	AP-2alphaA	36	45
					9.9.270	ETF	36	45
					2.3.1.0	Sp1	49	59
					.3.1.0 S	p1 1	5	24
					2.3.1.0	Sp1	27	41

	Mutated		G	8	2.3.2.1	Krox-20	30	39
					2.3.2.3	WT1	30	39
					9.9.270	ETF	30	39
					2.3.1.0	Sp1	34	47
					1.6.1.0	AP-2	36	45
					2.3.1.0	Sp1	49	59
rs1229558462	WILD	2:45651565	C	8	2.3.1.0	Sp1	15	24
					2.3.1.0	Sp1	30	44
					2.3.2.1	Egr-1	32	41
					2.3.2.3	GLI3	32	41
					9.9.270	ETF	32	41
					1.6.1.0	AP-2alphaA	36	45
					2.3.1.0	Sp1	36	45
					2.3.1.0	Sp1	49	59
	Mutated		A	7	2.3.1.0	Sp1	15	24
					2.3.2.1	Egr-1	30	39
					9.9.270	ETF	30	39
					2.3.1.0	Sp1	30	41
					2.3.2.3	WT1	32	41
					1.3.2.1	c-Myc	34	43
					2.3.1.0	Sp1	49	59
rs1221104800	WILD	2:45651573	T	8	2.3.1.0	Sp1	15	24
					2.3.1.0	Sp1	30	44
					2.3.2.1	Egr-1	32	41
					2.3.2.3	GLI3	32	41
					9.9.270	ETF	32	41
					1.6.1.0	AP-2alphaA	36	45
					2.3.1.0	Sp1	36	45
					2.3.1.0	Sp1	49	59
	Mutated		A	8	2.3.1.0	Sp1	15	24
					2.3.1.0	Sp1	30	44
					2.3.2.1	Egr-1	32	41
					2.3.2.3	GLI3	32	41
					9.9.270	ETF	32	41
					1.6.1.0	AP-2alphaA	36	45
					2.3.1.0	Sp1	36	45
					2.3.1.0	Sp1	49	59
	Mutated		C	9	2.3.1.0	Sp1	15	24
					2.3.1.0	Sp1	30	43
					2.3.2.1	Egr-1	32	41
					2.3.2.3	GLI3	32	41
					9.9.270	ETF	32	41
					1.6.1.0	AP-2alphaA	36	45
					2.3.1.0	Sp1	37	51
					9.9.539	NF-1	44	53
					2.3.1.0	Sp1	50	59
					2.3.1.0	Sp1	15	24
					2.3.1.0	Sp1	30	44
					2.3.2.1	Egr-1	32	41

	WILD		C	8	2.3.2.3	GLI3	32	41
					9.9.270	ETF	32	41
					1.6.1.0	AP-2alphaA	36	45
					2.3.1.0	Sp1	36	45
rs1293200978		2:45651574			2.3.1.0	Sp1	49	59
					2.3.1.0	Sp1	15	24
					2.3.1.0	Sp1	30	44
					2.3.2.1	Egr-1	32	41
	Mutated		G	7	2.3.2.3	GLI3	32	41
					9.9.270	ETF	32	41
					2.3.1.0	Sp1	37	46
					2.3.1.0	Sp1	49	58
					2.3.1.0	Sp1	15	24
					2.3.1.0	Sp1	30	44
					2.3.2.1	Egr-1	32	41
	WILD		A	8	2.3.2.3	GLI3	32	41
					9.9.270	ETF	32	41
					1.6.1.0	AP-2alphaA	36	45
					2.3.1.0	Sp1	36	45
rs543265725		2:45651575			2.3.1.0	Sp1	49	59
					2.3.1.0	Sp1	15	24
					2.3.1.0	Sp1	30	44
					2.3.2.1	Egr-1	32	41
	Mutated		T	8	2.3.2.3	GLI3	32	41
					9.9.270	ETF	32	41
					2.3.1.0	Sp1	37	46
					1.1.1.1	c-Jun	48	57
					1.1.1.2	c-Fos	49	58
					2.3.1.0	Sp1	15	24
					2.3.1.0	Sp1	30	44
					2.3.2.1	Egr-1	32	41
	WILD		G	8	2.3.2.3	GLI3	32	41
					9.9.270	ETF	32	41
					1.6.1.0	AP-2alphaA	36	45
rs931148603		2:45651576			2.3.1.0	Sp1	36	45
					2.3.1.0	Sp1	49	59
					2.3.1.0	Sp1	15	24
					2.3.2.1	Egr-1	32	41
	Mutated		C	5	2.3.2.3	GLI3	32	41
					9.9.270	ETF	32	41
					2.3.1.0	Sp1	32	46
					2.3.1.0	Sp1	15	24
					2.3.1.0	Sp1	30	44
					2.3.2.1	Egr-1	32	41
	WILD		A	8	2.3.2.3	GLI3	32	41
					9.9.270	ETF	32	41
					1.6.1.0	AP-2alphaA	36	45
					2.3.1.0	Sp1	36	45
rs1405481375		2:45651581			2.3.1.0	Sp1	49	59

					3.1.0 Sp	1	15	24
					2.3.2.1	Egr-1	32	41
					2.3.2.3	GLI3	32	41
	Mutated		G	7	9.9.270	ETF	32	41
					2.3.1.0	Sp1	32	43
					1.6.1.0	AP-2alphaA	36	45
					2.3.1.0	Sp1	50	62
					2.3.1.0	Sp1	15	24
					2.3.1.0	Sp1	30	44
					2.3.2.1	Egr-1	32	41
	WILD		C	8	2.3.2.3	GLI3	32	41
					9.9.270	ETF	32	41
					1.6.1.0	AP-2alphaA	36	45
					2.3.1.0	Sp1	36	45
					2.3.1.0	Sp1	49	59
					2.3.1.0	Sp1	15	24
					2.3.1.0	Sp1	30	44
					2.3.2.1	Egr-1	32	41
					2.3.2.3	GLI3	32	41
	Mutated		A	9	9.9.270	ETF	32	41
rs1446033604		2:45651584			1.6.1.0	AP-2alphaA	36	45
					2.3.1.0	Sp1	37	46
					1.2.1.0	E1	52	61
					1.2.2.0	MyoD	52	61
					2.3.1.0	Sp1	15	24
					2.3.1.0	Sp1	30	44
					2.3.2.1	Egr-1	32	41
					2.3.2.3	GLI3	32	41
	Mutated		T	10	9.9.270	ETF	32	41
					1.6.1.0	AP-2alphaA	36	45
					2.3.1.0	Sp1	37	46
					2.3.1.0	Sp1	51	60
					1.2.1.0	E1	52	61
					1.2.2.0	MyoD	52	61
					2.3.1.0	Sp1	31	42
	WILD		C	4	2.3.1.0	Sp1	50	62
					2.3.2.1	Krox-20	51	60
					1.6.1.0	AP-2	54	63
					2.3.1.0	Sp1	31	42
rs985257000	Mutated	2:45651586	G	4	2.3.1.0	Sp1	50	62
					2.3.2.1	Krox-20	51	60
					1.6.1.0	AP-2	54	63
					2.3.1.0	Sp1	31	42
	Mutated		T	4	2.3.1.0	Sp1	50	62
					2.3.2.1	Krox-20	51	60
					1.6.1.0	AP-2	54	63
					2.3.1.0	Sp1	31	42
	WILD		T	4	2.3.1.0	Sp1	50	62
					2.3.2.1	Krox-20	51	60

					1.6.1.0	AP-2	54	63
rs1290873009		2:45651587			2.3.1.0	Sp1	31	42
					2.3.1.0	Sp1	50	62
	Mutated		A	4	2.3.2.1	Krox-20	51	60
					1.6.1.0	AP-2	54	63
					2.3.1.0	Sp1	31	42
	WILD		A	4	2.3.1.0	Sp1	50	62
					2.3.2.1	Krox-20	51	60
					1.6.1.0	AP-2	54	63
rs1433602120		2:45651589			2.3.1.0	Sp1	31	42
	Mutated		G	4	2.3.1.0	Sp1	50	62
					2.3.2.1	Krox-20	51	60
					1.6.1.0	AP-2	54	63
					2.3.1.0	Sp1	31	42
	WILD		T	4	2.3.1.0	Sp1	50	62
					2.3.2.1	Krox-20	51	60
					1.6.1.0	AP-2	54	63
rs938002222		2:45651591			2.3.1.0	Sp1	31	42
	Mutated		A	4	2.3.1.0	Sp1	50	62
					2.3.2.1	Krox-20	51	60
					1.6.1.0	AP-2	54	63
					2.3.1.0	Sp1	31	42
	WILD		C	4	2.3.1.0	Sp1	50	62
					2.3.2.1	Krox-20	51	60
					1.6.1.0	AP-2	54	63
					2.3.1.0	Sp1	9	18
rs992016379		2:45651595			2.3.1.0	Sp1	31	42
	Mutated		A	5	2.3.1.0	Sp1	50	62
					2.3.2.1	Krox-20	51	60
					1.6.1.0	AP-2	54	63
	Mutated		T	4	2.3.1.0	Sp1	31	42
					2.3.1.0	Sp1	50	62
					2.3.1.0	Sp1	31	42
	WILD		**G**	4	2.3.1.0	sp1	50	62
					2.3.2.1	krox-20	51	60
rs1465811840		2:45651596			1.6.1.0	ap-2	54	63
					2.3.1.0	Sp1	31	42
			T	4	2.3.1.0	sp1	50	62
					2.3.2.1	krox-20	51	60
					1.6.1.0	ap-2	54	63
					2.3.1.0	Sp1	31	42
	WILD		**G**	4	2.3.1.0	sp1	50	62
					2.3.2.1	krox-20	51	60
rs1213772661		2:45651608			1.6.1.0	ap-2	54	63
					2.3.1.0	Sp1	31	42
	Mutated		C	4	2.3.1.0	sp1	50	62
					2.3.2.1	krox-20	51	60
					1.6.1.0	ap-2	54	63

rs971806465	WILD	2:45651609	**G**	4	2.3.1.0	Sp1	31	42
					2.3.1.0	sp1	50	62
					2.3.2.1	krox-20	51	60
					1.6.1.0	ap-2	54	63
	Mutated		A	4	2.3.1.0	Sp1	31	42
					2.3.1.0	sp1	50	62
					2.3.2.1	krox-20	51	60
					1.6.1.0	ap-2	54	63
rs1259533182	WILD	2:45651615	**A**	4	2.3.1.0	Sp1	31	42
					2.3.1.0	sp1	50	62
					2.3.2.1	krox-20	51	60
					1.6.1.0	ap-2	54	63
	Mutated		G	4	2.3.1.0	Sp1	31	42
					2.3.1.0	sp1	50	62
					2.3.2.1	krox-20	51	60
					1.6.1.0	ap-2	54	63
rs981682780	WILD	2:45651618	**T**	4	2.3.1.0	Sp1	31	42
					2.3.1.0	sp1	50	62
					2.3.2.1	krox-20	51	60
					1.6.1.0	ap-2	54	63
	Mutated		C	6	4.1.1.0	NF-kappaB1	24	33
					2.3.1.0	Sp1	31	41
					2.3.2.3	WT1	32	41
					2.3.1.0	Sp1	50	62
					2.3.2.1	Krox-20	51	60
					1.6.1.0	AP-2	54	63
rs687914	WILD	2:45651621	**G**	4	2.3.1.0	Sp1	31	42
					2.3.1.0	sp1	50	62
					2.3.2.1	krox-20	51	60
					1.6.1.0	ap-2	54	63
	Mutated		A	5	2.2.1.1	GATA-1	30	39
					2.3.1.0	Sp1	32	41
					2.3.1.0	Sp1	50	63
					2.3.2.1	Krox-20	51	60
					1.6.1.0	AP-2	54	63
rs687914	WILD	2:45651621	**G**	4	2.3.1.0	Sp1	31	42
					2.3.1.0	sp1	50	62
					2.3.2.1	krox-20	51	60
					1.6.1.0	ap-2	54	63
	Mutated		T	7	2.2.1.1	GATA-1	30	39
					2.3.1.0	Sp1	32	41
					1.6.1.0	AP-2	33	42
					2.3.3.0	CPE_binding_pro	34	43
					2.3.1.0	Sp1	50	63
					2.3.2.1	Krox-20	51	60
					1.6.1.0	AP-2	54	63
rs1558526560	Mutated		-	5	2.2.1.1	GATA-1	30	39
					2.3.1.0	Sp1	31	41
					2.3.1.0	Sp1	49	62

					2.3.2.1	Krox-20	50	59
					1.6.1.0	AP-2	53	62
	WILD		**T**	4	2.3.1.0	Sp1	31	42
					2.3.1.0	sp1	50	62
					2.3.2.1	krox-20	51	60
rs902664279		2:45651630			1.6.1.0	ap-2	54	63
	Mutated		A	4	2.3.1.0	Sp1	31	42
					2.3.1.0	Sp1	50	62
					2.3.2.1	Krox-20	51	60
					1.6.1.0	AP-2	54	63
	WILD		**C**	4	2.3.1.0	Sp1	31	42
					2.3.1.0	sp1	50	62
					2.3.2.1	krox-20	51	60
					1.6.1.0	ap-2	54	63
rs1224086295	Mutated	2:45651633	G	3	2.3.1.0	Sp1	31	42
					2.3.1.0	Sp1	50	63
					1.6.1.0	AP-2	54	63
	Mutated		T	4	2.3.1.0	Sp1	31	42
					2.1.1.1	GR	43	52
					2.3.1.0	Sp1	50	62
					1.6.1.0	AP-2	54	63
	WILD		**G**	4	2.3.1.0	Sp1	31	42
					2.3.1.0	sp1	50	62
					2.3.2.1	krox-20	51	60
					1.6.1.0	ap-2	54	63
rs1261920018	Mutated	2:45651640	A	5	2.3.1.0	Sp1	31	42
					2.3.3.0	MIG1	47	56
					9.9.590	NF-kappaB	49	58
					2.3.1.0	Sp1	49	62
					9.9.77	CACCC-binding_f	51	60
	Mutated		C	3	2.3.1.0	Sp1	31	42
					2.3.1.0	Sp1	47	61
					2.3.2.3	GLI3	50	59
	WILD		**C**	4	2.3.1.0	Sp1	31	42
					2.3.1.0	sp1	50	62
					2.3.2.1	krox-20	51	60
rs1000988694		2:45651642			1.6.1.0	ap-2	54	63
	Mutated		T	3	2.3.1.0	Sp1	31	42
					2.3.2.3	WT1	47	56
					2.3.1.0	Sp1	47	60
	WILD		G	6	2.3.1.0	Sp1	8	22
					2.3.1.0	Sp1	15	24
					1.1.3.0	C/EBPalpha	30	39
					3.4.1.0	HSTF	30	39
					4.1.1.0	NF-kappaB2	30	39
					9.9.590	NF-kappaB	30	39
					2.3.1.0	Sp1	8	22
					2.3.1.0	Sp1	15	24

rs1217420340	Mutated	2:45651645	A	6	1.1.3.0	C/EBPalpha	30	39
					3.4.1.0	HSTF	30	39
					4.1.1.0	NF-kappaB2	30	39
					9.9.590	NF-kappaB	30	39
					2.3.1.0	Sp1	8	22
					2.3.1.0	Sp1	15	24
	Mutated		C	6	1.1.3.0	C/EBPalpha	30	39
					3.4.1.0	HSTF	30	39
					4.1.1.0	NF-kappaB2	30	39
					9.9.590	NF-kappaB	30	39
					2.3.1.0	Sp1	8	22
					2.3.1.0	Sp1	15	24
	WILD		C	6	1.1.3.0	C/EBPalpha	30	39
					3.4.1.0	HSTF	30	39
					4.1.1.0	NF-kappaB2	30	39
					9.9.590	NF-kappaB	30	39
					2.3.1.0	Sp1	7	19
					2.3.1.0	Sp1	13	22
					2.3.1.0	Sp1	21	30
rs1390200563	Mutated	2:45651668	G	7	1.1.3.0	C/EBPalpha	30	39
					3.4.1.0	HSTF	30	39
					4.1.1.0	NF-kappaB2	30	39
					9.9.590	NF-kappaB	30	39
					2.3.1.0	Sp1	8	17
					2.3.1.0	Sp1	15	24
	Mutated		T	6	1.1.3.0	C/EBPalpha	30	39
					3.4.1.0	HSTF	30	39
					4.1.1.0	NF-kappaB2	30	39
					9.9.590	NF-kappaB	30	39
					2.3.1.0	Sp1	8	22
					2.3.1.0	Sp1	15	24
	WILD		A	6	1.1.3.0	C/EBPalpha	30	39
					3.4.1.0	HSTF	30	39
					4.1.1.0	NF-kappaB2	30	39
rs1426258125		2:45651678			9.9.590	NF-kappaB	30	39
					2.3.1.0	Sp1	8	22
					2.3.1.0	Sp1	15	24
	Mutated		T	5	3.4.1.0	HSF	30	39
					4.1.1.0	Dl	31	40
					1.3.2.3	E2F	32	41
					2.3.1.0	Sp1	8	22
					2.3.1.0	Sp1	15	24
	WILD		C	6	1.1.3.0	C/EBPalpha	30	39
rs1261533756		2:45651682			3.4.1.0	HSTF	30	39
					4.1.1.0	NF-kappaB2	30	39
					9.9.590	NF-kappaB	30	39
	Mutated		G	2	2.3.1.0	Sp1	8	22
					2.3.1.0	Sp1	15	24
					2.3.1.0	Sp1	8	22

					2.3.1.0	Sp1	15	24
	WILD		C	6	1.1.3.0	C/EBPalpha	30	39
					3.4.1.0	HSTF	30	39
					4.1.1.0	NF-kappaB2	30	39
					9.9.590	NF-kappaB	30	39
rs774739390		2:45651697			2.3.1.0	Sp1	8	22
					2.3.1.0	Sp1	15	24
					1.1.3.0	C/EBPalpha	30	39
	Mutated		T	8	3.4.1.0	HSTF	30	39
					4.1.1.0	NF-kappaB2	30	39
					9.9.590	NF-kappaB	30	39
					1.1.3.0	C/EBPalpha	42	53
					9.9.539	NF-1	48	57
					2.3.1.0	Sp1	8	22
					2.3.1.0	Sp1	15	24
	WILD		G	6	1.1.3.0	C/EBPalpha	30	39
					3.4.1.0	HSTF	30	39
					4.1.1.0	NF-kappaB2	30	39
rs1208941692		2:45651698			9.9.590	NF-kappaB	30	39
					2.3.1.0	Sp1	8	22
					2.3.1.0	Sp1	15	24
	Mutated		A	6	1.1.3.0	C/EBPalpha	30	39
					3.4.1.0	HSTF	30	39
					4.1.1.0	NF-kappaB2	30	39
					9.9.590	NF-kappaB	30	39
					2.3.1.0	Sp1	8	22
					2.3.1.0	Sp1	15	24
	WILD		G	6	1.1.3.0	C/EBPalpha	30	39
					3.4.1.0	HSTF	30	39
					4.1.1.0	NF-kappaB2	30	39
					9.9.590	NF-kappaB	30	39
rs1344558376		2:45651703			2.3.1.0	Sp1	8	22
					2.3.1.0	Sp1	15	24
					1.1.3.0	C/EBPalpha	30	39
	Mutated		A	7	3.4.1.0	HSTF	30	39
					4.1.1.0	NF-kappaB2	30	39
					9.9.590	NF-kappaB	30	39
					4.1.1.0	NF-kappaB1	52	61
					1.3.1.2	USF	14	23
					1.1.3.0	C/EBPalpha	28	37
					2.3.1.0	Sp1	33	47
	WILD		C	8	9.9.270	ETF	37	46
					1.6.1.0	AP-2alphaA	39	48
					2.3.1.0	Sp1	39	48
					2.3.2.1	Egr-1	39	48
rs1475822634		2:45651956			2.3.1.0	Sp1	48	57
					1.3.1.2	USF	14	23
					1.1.3.0	C/EBPalpha	28	37
					2.3.1.0	Sp1	33	47

	Mutated		G	8	9.9.270	ETF	37	46
					1.6.1.0	AP-2alphaA	39	48
					2.3.1.0	Sp1	39	48
					2.3.2.1	Egr-1	39	48
					2.3.1.0	Sp1	48	57
					1.3.1.2	USF	14	23
					1.1.3.0	C/EBPalpha	28	37
					2.3.1.0	Sp1	33	47
	WILD		G	8	9.9.270	ETF	37	46
					1.6.1.0	AP-2alphaA	39	48
					2.3.1.0	Sp1	39	48
					2.3.2.1	Egr-1	39	48
					2.3.1.0	Sp1	48	57
					1.1.3.0	C/EBPalpha	28	37
					2.3.1.0	Sp1	33	47
					9.9.270	ETF	37	46
rs918001784	Mutated	2:45651960	A	7	1.6.1.0	AP-2alphaA	39	48
					2.3.1.0	Sp1	39	48
					2.3.2.1	Egr-1	39	48
					2.3.1.0	Sp1	48	57
					1.3.1.2	USF	14	23
					1.1.3.0	C/EBPalpha	28	37
					2.3.1.0	Sp1	33	47
	Mutated		T	8	9.9.270	ETF	37	46
					1.6.1.0	AP-2alphaA	39	48
					2.3.1.0	Sp1	39	48
					2.3.2.1	Egr-1	39	48
					2.3.1.0	Sp1	48	57
					1.3.1.2	USF	14	23
					1.1.3.0	C/EBPalpha	28	37
					2.3.1.0	Sp1	33	47
	WILD		G	8	9.9.270	ETF	37	46
rs1270066804		2:45651986			1.6.1.0	AP-2alphaA	39	48
					2.3.1.0	Sp1	39	48
					2.3.2.1	Egr-1	39	48
					2.3.1.0	Sp1	48	57
	Mutated		T	2	1.3.1.2	USF	14	23
					2.3.1.0	Sp1	38	47
					2.3.1.0	Sp1	16	25
					2.3.1.0	Sp1	22	33
					3.6.1.0	TEC1	37	46
	WILD		G	6	2.3.1.0	Sp1	43	54
					9.9.561	NF-muE1	48	57
rs1443936062		2:45652035			2.3.1.0	Sp1	52	61
					2.3.1.0	Sp1	18	32
					3.6.1.0	TEC1	37	46
	Mutated		A	5	2.3.1.0	Sp1	43	54
					9.9.561	NF-muE1	48	57
					2.3.1.0	Sp1	52	61

					2.3.1.0	Sp1	16	25
					2.3.1.0	Sp1	22	33
	WILD		C	6	3.6.1.0	TEC1	37	46
					2.3.1.0	Sp1	43	54
					9.9.561	NF-muE1	48	57
					2.3.1.0	Sp1	52	61
rs750009110		2:45652088			2.3.1.0	Sp1	8	19
					9.9.561	NF-muE1	11	20
					2.3.1.0	Sp1	18	32
	Mutated		G	7	2.3.1.0	Sp1	25	34
					2.3.2.1	Egr-1	26	35
					2.1.1.1	GR	41	50
					4.3.2.0	SRF	53	62
					2.3.1.0	Sp1	16	25
					2.3.1.0	Sp1	22	33
	WILD		C	6	3.6.1.0	TEC1	37	46
					2.3.1.0	Sp1	43	54
					9.9.561	NF-muE1	48	57
					2.3.1.0	Sp1	52	61
					2.3.1.0	Sp1	8	19
rs755680404		2:45652090			9.9.561	NF-muE1	11	20
					2.3.1.0	Sp1	15	28
					1.6.1.0	AP-2	23	32
	Mutated		G	9	2.3.1.0	Sp1	23	35
					9.9.270	ETF	24	33
					2.3.2.1	Egr-1	27	36
					2.1.1.1	GR	41	50
					4.3.2.0	SRF	53	62
					2.3.1.0	Sp1	16	25
					2.3.1.0	Sp1	22	33
	WILD		C	6	3.6.1.0	TEC1	37	46
					2.3.1.0	Sp1	43	54
					9.9.561	NF-muE1	48	57
					2.3.1.0	Sp1	52	61
rs201815286		2:45652091			2.3.1.0	Sp1	10	24
					9.9.561	NF-muE1	11	20
					2.3.1.0	Sp1	16	25
	Mutated		T	7	9.9.537	NF-1	21	30
					2.3.1.0	Sp1	23	32
					2.1.1.1	GR	41	50
					4.3.2.0	SRF	53	62
					2.3.1.0	Sp1	16	25
					2.3.1.0	Sp1	22	33
	WILD		G	6	3.6.1.0	TEC1	37	46
					2.3.1.0	Sp1	43	54
					9.9.561	NF-muE1	48	57
					2.3.1.0	Sp1	52	61
rs753516084		2:45652092			2.3.1.0	Sp1	7	20
					9.9.561	NF-muE1	11	20

					2.3.1.0	Sp1	15	26
	Mutated		A	7	2.3.1.0	Sp1	21	32
					9.9.77	CACCC-binding_f	23	32
					2.1.1.1	GR	41	50
					4.3.2.0	SRF	53	62
					2.3.1.0	Sp1	16	25
					2.3.1.0	Sp1	22	33
	WILD		C	6	3.6.1.0	TEC1	37	46
					2.3.1.0	Sp1	43	54
					9.9.561	NF-muE1	48	57
					2.3.1.0	Sp1	52	61
rs1407190036		2:45652093			2.3.1.0	Sp1	8	20
					9.9.561	NF-muE1	11	20
					2.3.1.0	Sp1	15	25
	Mutated		A	7	2.3.1.0	Sp1	22	31
					1.3.2.1	c-Myc	23	32
					2.1.1.1	GR	41	50
					4.3.2.0	SRF	53	62
					2.3.1.0	Sp1	16	25
					2.3.1.0	Sp1	22	33
	WILD		C	6	3.6.1.0	TEC1	37	46
					2.3.1.0	Sp1	43	54
					9.9.561	NF-muE1	48	57
					2.3.1.0	Sp1	52	61
rs754742928		2:45652094			2.3.1.0	Sp1	8	22
					9.9.561	NF-muE1	11	20
					2.3.1.0	Sp1	15	28
	Mutated		A	8	2.3.3.0	MIG1	21	30
					2.3.1.0	Sp1	23	32
					4.4.1.0	E2	29	38
					2.1.1.1	GR	41	50
					4.3.2.0	SRF	53	62
					2.3.1.0	Sp1	16	25
					2.3.1.0	Sp1	22	33
	WILD		C	6	3.6.1.0	TEC1	37	46
					2.3.1.0	Sp1	43	54
					9.9.561	NF-muE1	48	57
rs900931286		2:45652095			2.3.1.0	Sp1	52	61
					2.3.1.0	Sp1	8	20
					9.9.561	NF-muE1	11	20
	Mutated		T	6	2.3.1.0	Sp1	15	26
					2.3.1.0	Sp1	23	33
					2.1.1.1	GR	41	50
					4.3.2.0	SRF	53	62
					2.3.1.0	Sp1	9	20
					9.9.561	NF-muE1	11	20
					2.3.1.0	Sp1	16	30
	WILD		G	7	9.9.270	ETF	21	30

					2.3.1.0	Sp1	22	33
					2.1.1.1	GR	41	50
					4.3.2.0	SRF	53	62
					2.3.1.0	Sp1	9	20
					9.9.561	NF-muE1	11	20
					2.3.1.0	Sp1	16	30
	Mutated		A	7	9.9.270	ETF	21	30
rs893058632		2:45652097			2.3.1.0	Sp1	22	33
					2.1.1.1	GR	41	50
					4.3.2.0	SRF	53	62
					2.3.1.0	Sp1	8	21
					9.9.561	NF-muE1	11	20
					2.3.1.0	Sp1	17	31
					9.9.270	ETF	21	30
	Mutated		C	9	2.3.2.1	Krox-20	23	32
					2.3.2.3	WT1	23	32
					2.3.1.0	Sp1	23	36
					2.1.1.1	GR	41	50
					4.3.2.0	SRF	53	62

Table 4b: Impact of PRKCE 3'UTR variants on the transcription fa

Variant ID	Allele type	Chr: bp	Alleles	TF-BS	Class
rs745359100	Wild	2:46010779	C	4	3.1.1.2<br>1.1.3.0<br>3.1.2.2<br>1.1.3.0
	Mutated		T	4	3.1.1.2<br>1.1.3.0<br>3.1.2.2<br>1.1.3.0
rs1558956262	Wild	2:46010780	T	4	3.1.1.2<br>1.1.3.0<br>3.1.2.2<br>1.1.3.0
	Mutated		A	5	3.1.1.2<br>2.1.1.1<br>1.1.3.0<br>3.1.2.2<br>1.1.3.0
rs746238647	Wild	2:46010781	C	4	3.1.1.2<br>1.1.3.0<br>3.1.2.2<br>1.1.3.0
	Mutated		--	4	3.1.1.2<br>1.1.3.0<br>3.1.2.2<br>1.1.3.0
rs769370864	Wild	2:46010784	T	4	3.1.1.2<br>1.1.3.0<br>3.1.2.2<br>1.1.3.0
	Mutated		G	5	3.1.1.2<br>2.3.1.0<br>1.1.3.0<br>3.1.2.2<br>1.1.3.0
rs1321440708	Wild	2:46010785	G	4	3.1.1.2<br>1.1.3.0<br>3.1.2.2<br>1.1.3.0
	Mutated		A	4	3.1.1.2<br>1.1.3.0<br>3.1.2.2<br>1.1.3.0
	Wild		A	4	3.1.1.2<br>1.1.3.0

	Wild		A	4	
					3.1.2.2
					1.1.3.0
rs1333919675		2:46010802			3.1.1.2
					1.1.3.0
	Mutated		G	6	3.1.2.2
					1.1.3.0
					4.1.1.0
					9.9.590
					3.1.1.2
	Wild		C	4	1.1.3.0
					3.1.2.2
rs571801707		2:46184892			1.1.3.0
					3.1.1.2
	Mutated		T	4	1.1.3.0
					3.1.2.2
					1.1.3.0
					3.1.1.2
	Wild		C	4	1.1.3.0
					3.1.2.2
rs777043604		2:46184900			1.1.3.0
					3.1.1.2
	Mutated		G	4	1.1.3.0
					3.1.2.2
					1.1.3.0
					3.1.1.2
	Wild		C	4	1.1.3.0
					3.1.2.2
rs1246626878		2:46010804			1.1.3.0
					3.1.1.2
	Mutated		T	4	1.1.3.0
					3.1.2.2
					1.1.3.0
	Wild		T	1	3.1.2.2
rs1004083544	Mutated	2:46010811	C	1	3.1.2.2
	Mutated		G	1	3.1.2.2
					4.3.1.1
	Wild		T	4	3.1.2.2
					3.1.2.2
rs1028379694		2:46010874			3.1.2.1
					3.1.2.2
	Mutated		C	3	3.1.2.2
					3.1.2.1
					4.3.1.1
	Wild		T	4	3.1.2.2
					3.1.2.2
rs974526523		2:46010876			3.1.2.1
					3.5.3.0

	Mutated		C	3	3.1.2.2<br>3.1.2.1
	Wild		A	4	4.3.1.1<br>3.1.2.2<br>3.1.2.2<br>3.1.2.1
rs1044257146		2:46010888			
	Mutated		A/G	2	3.1.2.2<br>4.3.1.1
	Wild		A	4	4.3.1.1<br>3.1.2.2<br>3.1.2.2<br>3.1.2.1
	Mutated		G	3	3.1.2.2<br>4.3.1.1<br>3.1.2.2
rs938609813		2:46010892			
	Mutated		T	5	3.1.2.2<br>4.3.1.1<br>1.1.3.0<br>3.1.2.2<br>4.5.1.0
	Wild		AAA	4	4.3.1.1<br>3.1.2.2<br>3.1.2.2<br>3.1.2.1
rs1238436678		2:46010900-46010902			
	Mutated		AAA/AA	4	4.3.1.1<br>3.1.2.2<br>3.1.2.2<br>3.1.2.1
	Wild		G	4	4.3.1.1<br>3.1.2.2<br>3.1.2.2<br>3.1.2.1
rs1329233194		2:46010904			
	Mutated		T	4	4.3.1.1<br>3.1.2.2<br>3.1.2.2<br>3.1.2.1

ctor binding sites

Trancription factor	Start	Stop
Antp	15	24
C/EBPalpha	38	50
1-Oct	45	54
C/EBPalpha	47	57
Antp	15	24
C/EBPalpha	38	50
1-Oct	45	54
C/EBPalpha	47	57
Antp	15	24
C/EBPalpha	38	50
1-Oct	45	54
C/EBPalpha	47	57
GR	33	42
C/EBPalpha	38	47
1-Oct	45	54
C/EBPalpha	47	57
Antp	15	24
Antp	15	24
C/EBPalpha	38	50
1-Oct	45	54
C/EBPalpha	47	57
Antp	15	24
C/EBPalpha	37	46
1-Oct	44	53
C/EBPalpha	46	56
Antp	15	24
C/EBPalpha	38	50
1-Oct	45	54
C/EBPalpha	47	57
Antp	15	24
Sp1	34	43
C/EBPalpha	39	50
1-Oct	45	54
C/EBPalpha	47	57
Antp	15	24
C/EBPalpha	38	50
1-Oct	45	54
C/EBPalpha	47	57
Antp	15	24
C/EBPalpha	38	47
1-Oct	45	54
C/EBPalpha	47	57
Antp	15	24
C/EBPalpha	38	50

1-Oct	45	54
C/EBPalpha	47	57
Antp	15	24
C/EBPalpha	38	50
1-Oct	45	54
C/EBPalpha	48	57
NF-kappaB1	51	60
NF-kappaB	51	60
Antp	15	24
C/EBPalpha	38	50
1-Oct	45	54
C/EBPalpha	47	57
Antp	15	24
C/EBPalpha	38	50
1-Oct	45	54
C/EBPalpha	47	57
Antp	15	24
C/EBPalpha	38	50
1-Oct	45	54
C/EBPalpha	47	57
Antp	15	24
C/EBPalpha	38	50
1-Oct	45	54
C/EBPalpha	47	57
Antp	15	24
C/EBPalpha	38	50
1-Oct	45	54
C/EBPalpha	47	57
Antp	15	24
C/EBPalpha	38	50
1-Oct	45	54
C/EBPalpha	47	57
1-Oct	43	52
1-Oct	43	52
1-Oct	43	52
MEB-1	8	17
1-Oct	9	18
1-Oct	21	30
Pit-1a	22	31
1-Oct	9	18
1-Oct	21	30
Pit-1a	22	31
MEB-1	8	17
1-Oct	9	18
1-Oct	21	30
Pit-1a	22	31
ICSBP	10	19

1-Oct	21	30
Pit-1a	22	31
MEB-1	8	17
1-Oct	9	18
1-Oct	21	30
Pit-1a	22	31
1-Oct	8	17
MEB-1	8	17
MEB-1	8	17
1-Oct	9	18
1-Oct	21	30
Pit-1a	22	31
1-Oct	8	17
MEB-1	8	17
1-Oct	20	29
1-Oct	8	17
MEB-1	8	17
C/EBPalpha	19	28
1-Oct	21	31
TBP	24	33
MEB-1	8	17
1-Oct	9	18
1-Oct	21	30
Pit-1a	22	31
MEB-1	8	17
1-Oct	9	18
1-Oct	21	30
Pit-1a	22	31
MEB-1	8	17
1-Oct	9	18
1-Oct	21	30
Pit-1a	22	31
MEB-1	8	17
1-Oct	9	18
1-Oct	21	30
Pit-1a	22	31

Table 4c: Transcription factor sites and their co-regulation along with the LogP values estimated thr

Transcription factor binding site	Transcription factor	Co-regulation	Organ	LogP
5' UTR				
AP-1	AP-1	SRF	Heart	17.53
			Small intestine	6.29
		GR	Larynx	10.86
		CACCC-binding factor	Larynx	9.01
		C/EBPDELTA	Heart	7.53
		ETF	Ovary	6.72
		HSF-1	Bladder	6.51
		ETF	Mammary gland	6.19
		GATA-1	Heart	6.26
C/EBPDELTA	C/EBPDELTA	GR	Liver	10.81
		USF	Larynx	8.343
			Muscle	6.29
			Thymus	6.23
		MyoD	Peripheral nervous system	8.25
		AP-1	Heart	7.53
E2F	E2F	SRF	Small intestine	18.79
			Heart	9.15
		Sp1	Tongue	10.36
			Testis	8.83
			Small intestine	8.73
		c-Myc	Cervix	8.93
		AP-2	Brain	8.81
			Small intestine	6.98
		E2F	Cervix	8.44
		NF-KappaB	Thymus	8.33
			Soft tissue	7.61
			Lung	6.57
		GR	Soft tissue	7.93
			Bone	6.19
			Thymus	6.17
		SRF	Larynx	7.46
		CACCC-binding factor	Cervix	7.22
GATA-1	GATA-1	GR	Thymus	10.06
			Bone marrow	6.27
		AP-1	Heart	6.26
		C/EBPALPHA	Brain	9.35

GATA-1	GATA-1	USF	Muscle	8.76
		cMyc	Heart	8.01
		MyoD	Muscle	6.9
		SRF	Brain	6.87
GR	GR	AP-1	Larynx	10.86
		C/EBPDELTA	liver	10.81
		HSF1	Muscle	10.11
		GATA-1	Thymus	10.06
			Bone marrow	6.27
		NF-KappaB	Soft tissue	8.27
		CHOP-C/EBPALPHA	Brain	7.63
			Bone marrow	7.37
		sp1	Testis	7.17
		SRF	Pancreas	6.27
MyoD	MyoD	NF-1	Muscle	13.56
		SRF	Heart	11.93
		sp1	Pancreas	9.25
			Small intestine	6.43
		C/EBPDELTA	Peripheral nervous system	8.25
		USF	Muscle	8.15
		AP-2	Pancreas	7.57
		GATA-1	Muscle	6.9
**3' UTR**				
GR	GR	TBP	Small intestine	8.43
		NF-kappaB	Soft tissue	8.27
		CHOP-C/EBPalpha	Brain	7.63
			Bone marrow	7.37
		Sp1	Testis	7.17
SP1	SP1	GR	Testis	7.17
ICSBP	ICSBP	TBP	Small intestine	7.1
NF-KappaB	NF-KappaB	GR	Soft tissue	8.27
		NF-kappaB (P50)	Tongue	33.25
			Small intestine	10.56
			Spleen	8.26
			Lymph node	7.82
NF-KappaB1	NF-KappaB1	NF-kappaB	Tongue	33.25
		CHOP-C/EBPalpha	Brain	6.54
TBP	TBP	GR	Small intestine	8.43
		ICSBP	Small intestine	7.1

ough TiGER database
