# Supplementary material for: PRKCE non-coding variants influence on transcription as well as translation of its gene
Source: RNA Biol. 2022 Oct 26;19(1):1115–29. doi: 10.1080/15476286.2022.2139110 (PMC9621080; doi:10.1080/15476286.2022.2139110)
Supplement: Supplemental Material [file KRNB_A_2139110_SM6803.zip › ST5.pdf]

Table 5a: PRKCE 5'UTR variants impact on the structural stability of the PRKCE mRNA

				Thermodynamic ensemble prediction				RNAStructure	
**Variant ID**	**Chr: bp**	**Alleles**	**Stability**	**Minimum free energy(Kcal/mol)**	**Thermodynamic ensemble free energy**	**MFE structure frequency**	**Ensemble diversity**	**RNAstructure Fold Results**	**RNAstructure MaxExpert Results**
Wild	2:46000911	C		-1.6	-1.73	81.12%	1.4	-1.6	1.9
rs569884823	2:46000911	C/G	More	-2.5	-2.55	92.68 %	0.53	-2.5	2.0
Wild	2:46000912	T		0	-0.72	30.85 %	3.8	0	1.5
rs1227344174	2:46000912	T/G	more	-0.8	-1.38	38.94 %	2.31	-0.8	1.8
WILD	2:46000976	C		-2.6	-3.36	29.31 %	5.13	-2.6	1.8
rs1302848957	2:46000976	C/T	No	-2.6	-3.19	38.15 %	3.76	-2.6	1.7
Wild	2:46000977	C		-2.6	-3.37	28.70 %	5.33	-2.6	1.8
rs946217897	2:46000977	C/G	Less	-3.9	-3.97	88.58 %	0.91	-3.9	2.2
rs946217897	2:46000977	C/T	No	-2.6	-2.82	69.64 %	3.35	-2.6	2.0
WILD	2:45651351	G		-3.7	-4.29	38.27 %	3.76	-4.5	1.6
rs1444088897	2:45651351	G/A	Less	-2.2	-3.09	23.44 %	5.59	-2.2	1.3
WILD	2:45651352	C		-4.4	-5.23	26.07 %	6.97	-6.2	1.7
rs1279310031	2:45651352	C/T	Less	-4.4	-4.86	47.67 %	3.83	-4.4	1.6
WILD	2:45651354	C		-5.2	-5.35	77.90 %	2.72	-6.2	2
rs538954895	2:45651354	C/G	Less	-5	-5.56	40.45 %	5.48	-4.5	1.2
rs538954895	2:45651354	C/T	Less	-3.8	-4.58	28.39 %	5.55	-4.3	1.6
WILD	2:45651355	G		-3.7	-4.01	60.63 %	4.43	-3.7	1.5
rs1299335294	2:45651355	G/A	Less	-0.9	-1.34	49.36 %	3.09	-0.9	1.7
WILD	2:45651362	G		-2.1	-2.90	27.17 %	4.87	-2.9	*1.6*
rs1363740502	2:45651362	G/A	*Less*	-2.1	-2.52	50.44 %	2.06	-2.1	*1.8*
WILD	2:45651364			-3.5	-3.90	52.08 %	3.30	-3.5	1.6
rs912480755	2:45651364	G/-	Less	-3	-3.48	45.90 %	3.92	-3	1.4
WILD	2:45651364			-3.5	-3.90	52.08 %	3.30	-3.5	1.6
rs61762789	2:45651364	G/A	No	-3.5	-3.82	59.18 %	2.24	-3.5	1.8
WILD				-4.2	-4.32	81.98 %	0.67	-4.2	2
rs1226860990	2:45651365	C/A	No	-4.2	-4.29	86.05 %	0.37	-4.2	2
WILD				-4	-4.17	76.14 %	1.06	-4	1.9
rs558553528	2:45651369	C/G	No	-4	-4.37	55.26 %	3.55	-4	1.6
WILD				-1.5	-2.16	34.30 %	3.42 .	-1.5	1.6
rs1414250098	2:45651372	G/A	No	-1.5	-2.08	38.95 %	2.98	-1.5	1.7
WILD				-7.2	-7.80	37.48 %	5.62	-7.2	1.4
rs1272097179	2:45651437	A/C	No	-7.2	-7.9	31.89 %	6.95	-7.2	1.4
WILD				-8.5	-8.70	71.93 %	0.98	-6.6	1.2
rs1379339597	2:45651444	G/A	More	-7.5	-7.7	71.73 %	0.98	-8.5	1.9
WILD				-10.2	-10.69	45.10 %	7.20	-7.5	1.9
rs1055751280	2:45651452	C/T	More	-10.2	-10.7	44.68	7.22	-10.2	1.3
WILD				-10.5	-10.56	91.32 %	0.34	-10.2	1.3
rs1447949565	2:45651455	C/T	More	-10.5	-10.56	91.32 %	0.34	-10.5	2
WILD				-7.3	-7.95	34.95 %	6	-10.5	2
rs1253643130	2:45651503	C/T	Less	-7.3	-7.95	34.95 %	6	-7.3	1.5
WILD				-7.1	-8.18	17.25 %	6.88	-7.3	1.5
rs1182602720	2:45651504	A/G	More	-7.2	-8.25	18.21 %	7.29	-7.5	1.4
WILD				-1.4	-1.57	76.26 %	1.40	-7.5	1.3
rs1482628581	2:45651509	C/G	Less	-2	-2.47	46.79 %	3.02	-1.4	1.9
rs1482628581	2:45651509	C/T	Less	0	-0.56	40.43 %	3.44	-2	1.7
WILD				-3.6	-3.8	72.18 %	0.43	0.3	1.5
rs1000540686	2:45651547	T/C	More	-3.6	-3.8	72.19 %	0.42	-3.6	2

WILD				0	-0.12	82.80 %	0.74	-3.6	2
rs1350500051	2:45651549	C/G	Less	0	-0.85	25.00 %	2.52	0	1.8
WILD				0	-0.02	97.50 %	0.09	0	1.7
rs998706962	2:45651553	C/A	No	0	-0.02	97.50 %	0.09	0	1.8
WILD				-1.1	-1.27	76.49 %.	1.06	0	1.8
rs965329334	2:45651560	C/A	more	-1.5	-1.6	84.49 %	0.78	0	1.7
rs965329334	2:45651560	C/T	Less	-0.9	-1.12	70.54 %	1.26	0	1.8
rs965329334	2:45651560	C/G	More	-4.1	-4.35	66.81 %	3.39	0	1.7
WILD				-5.5	-5.65	77.77 %	3.08	-2.6	1.6
rs1229558462	2:45651565	C/A	More	-5.5	-5.66	77.68 %	3.09	-5.5	2
WILD				-2.8	-3.28	45.72 %	3.84	-5.5	1.8
rs1221104800	2:45651573	T/A	Less	-2.8	-3.29	45.30 %	3.86	-2.8	1.6
rs1221104800	2:45651573	T/C	Less	-2.8	-3.5	32.15 %	4.72	-2.8	1.6
WILD				-3.2	-3.57	54.48 %	3.78	-3	1.4
rs1293200978	2:45651574	C/G	More	-3.2	-3.56	55.86 %	3.52	-3.2	1.7
WILD				-3.6	-4.38	28.06 %	5.69	-3.2	1.8
rs543265725	2:45651575	A/T	More	-4.4	-4.85	48.19 %	6.39	-3.6	1.3
WILD				-7	-7.09	85.94 %	0.60	-4.5	1.4
rs931148603	2:45651576	G/C	More	-7	-7.09	85.94 %	0.6	-7	2
WILD				-1.8	-2.39	38.23 %	4.31	-7	2
rs1405481375	2:45651581	A/G	*Less*	-1.8	-2.38	39.06 %.	4.24	-1.8	*1.4*
WILD				-2.1	-2.75	35.06 %	5.43	-1.8	*1.4*
rs1446033604	2:45651584	C/A	More	-1.9	-2.31	51.27 %	2.63	-2.1	1.3
rs1446033604	2:45651584	C/T	Less	-1.9	-2.32	50.96 %	2.68	-1.9	1.3
WILD				-6.9	-6.95	92.05 %	1.20	-1.9	1.8
rs985257000	2:45651586	C/G	No	-6.9	-6.91	99.09 %	0.02	-1.9	1.8
rs985257000	2:45651586	C/T	More	-6.9	-6.91	99.11 %	0.02	-7	2.1
WILD				-6.9	-6.95	91.88 %	1.22	-7	2.1
rs1290873009	2:45651587	T/A	No	-6.9	-6.97	89.38 %	1.72	-7	2.1
WILD				-7	-7.05	92.90 %	1.07	-7	2
rs1433602120	2:45651589	A/G	More	-7.1	-7.14	93.79 %	0.93	-7	1.9
WILD				-5.3	-5.32	96.20 %	0.48	-7	1.9
rs938002222	2:45651591	T/A	Less	-5.1	-5.25	77.88 %	0.85	-7.1	1.9
WILD				-1.2	-1.34	79.95 %	0.89	-5.4	2
rs992016379	2:45651595	C/A	Less	0	-0.33	58.71 %	1.51	-5.2	2
rs992016379	2:45651595	C/T	Less	0	-0.3	61.33 %	1.38	-1.2	1.9
rs1465811840 (WILD)	2:45651596	G		0	-0.16	76.59 %	0.86	0	1.7
rs1465811840	2:45651596	G/T	No	0	-0.39	53.07 %	2.50	0	1.7
rs1213772661 (WILD)	2:45651608	G		0	-0.62	36.58 %	3.99	0	1.8
rs1213772661	2:45651608	G/C	More	0	-0.45	47.88 %	3.3	1	1.6
rs971806465 (WILD)	2:45651609	G		-1.3	-1.61	60.81 %	2.43	-0.3	1.6
rs971806465	2:45651609	G/A	Less	-1.9	-2.15	66.91 %	1.39	0.9	1.5
rs1259533182 (WILD)	2:45651615	A/G		-3.8	-4.23	50.08 %	2.62	-2.2	1.9
rs1259533182	2:45651615	A/G	Less	-3.8	-4.22	50.91 %	2.40	-1.9	1.8
rs981682780 (WILD)	2:45651618	T		-0.2	-1.08	24.10 %	3.79	-3.8	1.7
rs981682780	2:45651618	T/C	More	-1.1	-1.78	33.24 %	4.51	-3.8	1.7
rs687914 (WILD)	2:45651621	G		-0.5	-0.94	48.58 %	2.75	-0.2	1.5
rs687914	2:45651621	G/A	More	-0.2	-0.76	40.54 %	2.78	-1.1	1.5
rs687914	2:45651621	G/T	Less	0	-0.47	46.62 %	2.65	0	1.5
rs1558526560 (WILD)	2:45651621	G		-0.5	-0.94	48.58 %	2.75	0	1.5

rs1558526560	2:45651621	G/-	more	-1.3	-1.5	72.45 %	1.68	0	1.6
rs902664279 (WILD)	2:45651630	T		-0.6	-1.39	27.78 %	3.69	-4	3.6
rs902664279	2:45651630	T/A	Less	-0.7	-1.45	29.79 %	3.7	0.5	1.4
rs1224086295 (WILD)	2:45651633	C/G/T		-1	-1.52	43.08 %	3.41	-0.6	1.6
rs1224086295	2:45651633	C/G	more	-1.1	-2.14	18.59 %.	5.07	-0.6	1.6
rs1224086295	2:45651633	C/T	more	-1.1	-1.49	45.33 %	3.25	-1	1.7
rs1261920018 (WILD)	2:45651640	G/A/C		0	-0.12	82.61 %	0.62	-1.1	1.4
rs1261920018	2:45651640	G/A	Less	0	-0.04	93.61 %.	0.23	-1	1.7
rs1261920018	2:45651640	G/C	No	0	-0.02	97.41 %	0.10	0	1.8
rs1000988694	2:45651642	C/T		0	-0.14	80.28 %	0.75	0	1.8
rs1000988694	2:45651642	C/T	No	0	-0.15	78.22 %	0.82	0	1.9
rs1217420340	2:45651645	G/A/C		0	-0.29	62.08 %	1.52	0	1.8
rs1217420340	2:45651645	G/A	No	0	-0.12	82.59 %.	0.58	0	1.8
rs1217420340	2:45651645	G/C	No	0	-0.12	82.59 %	0.58	0	1.8
rs1390200563	2:45651668	C/G/T		-1.1	-1.75	34.79 %.	3.62	0	1.8
rs1390200563	2:45651668	C/G	More	-4.7	-4.91	71.03 %	2.28	0	1.8
rs1390200563	2:45651668	C/T	More	-1.3	-1.79	45.44 %.	3.22	-1.1	1.6
rs1426258125	2:45651678	A/T		-2.4	-2.91	43.42 %.	3.91	-4.7	1.9
rs1426258125	2:45651678	A/T	Less	-2.4	-2.89	44.94 %	3.66	-1.3	1.6
rs1261533756	2:45651682	C/G		-0.2	-0.88	33.30 %.	2.73	-2.7	1.7
rs1261533756	2:45651682	C/G	No	-0.2	-1.52	11.69 %	3.20	-2.7	1.7
rs774739390	2:45651697	C/T		0	-0.81	26.89 %	3.78	-0.2	1.7
rs774739390	2:45651697	C/T	Less	0	-0.75	29.85 %	3.37	-1	1.6
rs1208941692	2:45651698	G/A		0	-0.86	24.86 %	3.30 .	0.2	**1.6**
rs1208941692	2:45651698	G/A	No	0	-0.84	25.43 %	3.18	0.2	1.6
rs1344558376	2:45651703	G/A		0	-0.09	86.21 %	0.45	-0.2	1.7
rs1344558376	2:45651703	G/A	No	0	-0.03	94.69 %	0.19	-0.2	1.7
rs1475822634	2:45651956	C/G		6.1	-6.24	80.06 %.	2.46	0	1.5
rs1475822634	2:45651956	C/G	Less	-4	-4.51	43.78 %	2.36	0	1.5
rs918001784	2:45651960	G/A/T		-5.1	-5.25	78.68 %.	1.38	-6.3	2
rs918001784	2:45651960	G/A	Less	-4	-4.04	94.12 %	0.41	-4.2	2
rs918001784	2:45651960	G/T	Less	-1.1	-1.73	35.79 %.	3.22	-5.4	2
rs1270066804	2:45651986	G/T		-2	-2.39	53.53 %.	1.02 .	-4	2
rs1270066804	2:45651986	G/T	Less	-2	-2.39	53.53 %	1.01	-3.5	2
rs1443936062	2:45652035	G/A		-5.2	-5.44	67.81 %	1.74 .	-2	1.9
rs1443936062	2:45652035	G/A	Less	-5.1	-5.37	64.94 %	1.91	-2	1.9
rs986017109	2:45652046	A/C		-1.2	-1.39	73.57 %.	1.68	-5.2	1.9
rs986017109	2:45652046	A/C	Less	-1.2	-1.54	57.82 %.	2.55	-5.1	1.9
rs772170785	2:45652050	C/T		-1.8	-2.11	60.17 %.	2.73 .	-6.7	2.8
rs772170785	2:45652050	C/T	Less	-1	-1.24	67.94 %.	1.52	-6.7	2.8
rs1259533133	2:45652052	G/A		-4.6	-4.8	72.67 %.	2.98	-6.9	3
rs1259533133	2:45652052	G/A	Less	-2.4	-3.1	31.91 %.	4.13	-1.9	1.9
rs773211026	2:45652054	C/A/T		-9.1	-9.14	93.26 %	0.16	-4.6	1.7
rs773211026	2:45652054	C/A	Less	-3.4	-4.05	34.58 %	4.13 .	-2.4	1.5
rs773211026	2:45652054	C/T	More	-6.1	-6.2	85.44 %.	0.71	-9.1	2
rs753172025	2:45652082	C/T		-0.2	-0.78	38.73 %.	2.45	-3.4	1.6
rs753172025	2:45652082	C/T	More	-0.2	-0.79	38.36%	2.47	-6.1	2.0
rs371492921	2:45652083	C/T		-0.6	-0.98	54.27 %.	2.21 .	-0.2	1.7
rs371492921	2:45652083	C/T	No	-0.6	-0.93	58.72 %	1.92 .	-0.2	1.7
rs750009110	2:45652088	C/G		-2	-2.07	89.73 %.	0.75	-0.6	1.9

rs750009110	2:45652088	C/G	More	-2.3	-2.66	55.32 %	3.07	-0.6	-0.6
rs755680404	2:45652090	C/G		0	-0.09	85.83 %	0.49	-2	2.0
rs755680404	2:45652090	C/G	More	0	-0.17	75.70 %	0.84	-2.3	1.7
rs201815286	2:45652091	C/T		0	-0.09	85.83 %	0.49	0	1.8
rs201815286	2:45652091	C/T	No	0	-0.05	91.87 %	0.28 .	0	1.8
rs753516084	2:45652092	G/A		0	-0.09	85.83 %	0.49	0	1.8
rs753516084	2:45652092	G/A	No	0	-0.04	94.07 %	0.21	0	1.8
rs1407190036	2:45652093	C/A		0	-0.1	84.92 %	0.54	0	1.8
rs1407190036	2:45652093	C/A	No	0	-0.11	84.03 %	0.57	0	1.9
rs754742928	2:45652094	C/A		0	-0.59	38.25%	2.22	0	1.8
rs754742928	2:45652094	C/A	No	0	-0.62	36.35 %	2.03	0	1.8
rs900931286	2:45652095	C/T		-0.9	-1.42	43.09 %	3.01	0	1.7
rs900931286	2:45652095	C/T	More	-2.2	-2.31	83.09 %.	0.59	0	**1.7**
rs893058632	2:45652097	G/A/C		-1.4	-1.69	62.89 %	2.42	-0.9	1.6
rs893058632	2:45652097	G/A	More	-1.4	-1.86	47.43 %	3.11	-2.2	2.0
rs893058632	2:45652097	G/C	More	-1.4	-1.73	58.72 %	2.76	-1.4	1.8
								-1.4	1.6
								-1.4	1.8

Table 5b: PRKCE 3'UTR variants impact on the structural stability of the PRKCE mRNA

Variant ID	Chr: bp	Alleles	Stability	Thermodynamic ensemble prediction				RNAStructure	
				Minimum free energy(Kcal/mol)	Thermodynamic ensemble free energy	MFE structure frequency	Ensemble diversity	RNAstructure Fold Res	RNAstructure MaxExpert R
Wild rs745359100	2:46010779	C		-1	-1.45	47.96 %	1.93	-1	1.8
rs745359100	2:46010779	C/T	Less	0	-0.40	51.89 %	2.97 .	1	1.5
Wild rs1558956262	2:46010780	T		-2.1	-2.48	54.14 %	1.74	-2.1	1.8
rs1558956262	2:46010780	T/A	Less	-1.9	-2.54	35.29 %	2.29	-1.9	1.7
Wild rs746238647	2:46010781	C		-3.9	-4.16	65.98 %	0.86	-3.9	1.9
rs746238647	2:46010781	C/-	More	-4	-4.26	65.97 %	0.86	-4.0	1.8
Wild rs769370864	2:46010784	T		-1.6	-1.80	72.27 %	0.99	-1.6	2.0
rs769370864	2:46010784	T/G	Less	-1.4	-1.73	58.34 %	1.39	-1.4	1.9
Wild rs1321440708	2:46010785	G		-1.4	-1.66	65.43 %	1.17	-1.4	1.9
rs1321440708	2:46010785	G/A	Less	0	-0.02	97.12 %	0.14	0	1.8
Wild rs1333919675	2:46010802	A		-1.8	-2.13	59.01 %	1.31	-1.8	1.9
rs1333919675	2:46010802	A/G	More	-2	-2.31	60.19 %	1.17	-2.0	1.9
Wild rs571801707		c.*11C>T		-2.7	-3.04	57.81 %	1.21	-2.7	1.9
rs571801707		c.*11C>T	No	-2.7	-3.09	52.82 %	2.33	-2.7	1.8
rs777043604		c.*19C>G	No	-2.7	-3.03	58.31 %	1.11	-2.7	1.9
Wild rs1246626878	2:46010804	C		-1.7	-2.08	54.07 %	1.28	-1.7	1.8
rs1246626878	2:46010804	C/T	Less	0	-0.78	28.20 %	3.39	0	1.5
Wild rs1004083544	2:46010811	T		-3	-3.85	25.12 %	1.79	-3.4	1.8
rs1004083544	2:46010811	T/C/G	No	-3	-3.85	25.10 %	1.8	-3.4	1.8
rs1004083544	2:46010811	T/C/G	Less	-3	-3.85	25.82 %	2.62	-3.3	1.7
Wild rs1028379694	2:46010874	T		0	-0.1	84.47 %	0.72	0	1.8
rs1028379694	2:46010874	T/C	No	0	-0.15	78.92 %	1.08	0	1.7
Wild rs974526523	2:46010876	T		0	-0.02	96.11 %	0.16	0	1.8
rs974526523	2:46010876	T/C	No	0	-0.12	82.45 %	0.56	0	1.8
Wild rs1044257146	2:46010888	A		-1.9	-2.35	47.93 %	1.26	-1.9	1.9
rs1044257146	2:46010888	A/G	Less	-1.7	-2.1	52.60 %	1.32	-1.7	1.9
Wild rs938609813	2:46010892	A		0	-0.13	80.62 %	0.81	0	1.8
rs938609813	2:46010892	A/G	No	0	-0.21	71.22 %	1.11	0	1.6
rs938609813	2:46010892	A/T	More	-1	-1.23	69.22 %	1.33	-1.0	1.9
Wild rs1238436678	2:46010900-46010902	AAA		-0.5	-0.83	58.88 %	1.85	-0.5	2.1
rs1238436678	2:46010900-46010902	AAA/AA	No	-0.5	-0.86	55.85 %	2.17	-0.5	1.9
Wild rs1329233194	2:46010904	G		-1.4	-1.7	61.37 %	1.95	-1.4	1.9
rs1329233194	2:46010904	G/T	Less	0	-0.43	50.09 %	2.86	0.8	1.6

esults
