# Supplementary material for: PRKCE non-coding variants influence on transcription as well as translation of its gene
Source: RNA Biol. 2022 Oct 26;19(1):1115–29. doi: 10.1080/15476286.2022.2139110 (PMC9621080; doi:10.1080/15476286.2022.2139110)
Supplement: Supplemental Material [file KRNB_A_2139110_SM6803.zip › ST6.pdf]

Supplementary table 6a: List of miRNAs that binds with 5'UTR of PRKCE gene along with their binding energies

	Mirna	Accession no	RsID	MFE (kcal/mol)	Position	Agreement
			**Wild**	-20.6	3	1.154177301
			rs1302848957 C/T	-21	3	
	hsa-mir-3978	MIMAT0019363	rs946217897C/T	-20.6	3	
			rs946217897C/G	-20.6	3	
1			rs1444088897 G/A	-20.6	3	1.099324626
	hsa-mir-9-3p	MIMAT0000442	Wild	-12.8	3	1.099324626
2			rs1279310031 C/T	-12.8	3	
			wild	-14.2	1	
			rs1279310031 C/T	-14.2	1	
	hsa-mir-5585-3p	MIMAT0022286	rs538954895 C/G	-14.2	1	1.044471951
			rs538954895 C/T	-14.2	1	
3			rs1299335294 G/A	-14.2	1	
			Wild	-16.8	1	
			rs1279310031 C/T	-16.8	1	1.071898288
	hsa-mir-4728-3p	MIMAT0019850	rs538954895 C/G	-19.6	8	
			rs538954895 C/T	-17.4	1	
4			rs1299335294 G/A	-16.8	1	
			Wild	-18.4	2	
			rs1279310031 C/T	-18.4	2	
			rs538954895 C/G	-21.5	2	
			rs538954895 C/T	-18.3	2	1.099324626
	hsa-mir-4799-3p	MIMAT0019977	rs1299335294 G/A	-19.1	2	
			Rs1363740502 G/A	-18.4	2	
			Rs912480755 G/-	-18.4	2	
5			Rs61762789 G/A	-18.4	2	1.113037794
			wild	-19.6	2	
			Rs1226860990 C/A	-15.4	3	
	hsa-mir-6862-5p	MIMAT0027625	Rs558553528 C/G	-15.6	2	
			Rs1414250098 G/A	-19.7	3	
6			Rs1363740502 G/A	-19.6	2	
			wild	-16.9	2	1.05818512
			Rs1363740502 G/A	-12.9	3	
			Rs912480755 G/-	-15.2	2	
	hsa-mir-499b-5p	MIMAT0019897	Rs61762789 G/A	-16.9	2	
			Rs1226860990 C/A	-15.8	4	
			Rs558553528 C/G	-13.7	2	1.126750963
7			Rs1414250098 G/A	-17.6	2	
			wild	-10.6	11	
			Rs912480755 G/-	-10.6	9	
	hsa-mir-641	MIMAT0003311	Rs61762789 G/A	-10.6	11	
			Rs1226860990 C/A	-12.4	2	1.154177301
			Rs558553528 C/G	-11.8	6	
8			Rs1414250098 G/A	-9.3	11	1.154177301
			wild	-14.5	3	
			Rs912480755 G/-	-14.5	2	1.161033885
	hsa-mir-3617-5p	MIMAT0017997	Rs61762790 G/A	-14.5	3	
			Rs1226860990 C/A	-12.8	4	1.044471951
			Rs558553528 C/G	-16.7	3	
9			Rs1414250098 G/A	-15.3	3	
			wild	-21.5	5	1.044471951
	hsa-mir-4696	MIMAT0019790	Rs558553528 C/G	-21.5	5	
10			Rs1414250098 G/A	-21.5	5	1.140464132
			wild	-13.9	2	
	hsa-mir-4457	MIMAT0018979	Rs558553528 C/G	-13.7	1	1.277595819
11			Rs1414250098 G/A	-14.9	2	
			wild	-15.7	3	
	hsa-mir-3692-3p	MIMAT0018122	Rs558553528 C/G	-15.4	3	1.154177301
12			Rs1414250098 G/A	-16.4	3	
			wild	-11.1	4	
	hsa-mir-3145-3p	MIMAT0015016	Rs1272097179 A/C	-13	11	1.071898288
			Rs1379339597 G/A	-11.1	4	
13			Rs1055751280 C/T	-11.1	4	
			Wild	-8.7	13	1.277595819
	hsa-mir-607	MIMAT0003275	Rs1272097179s A/C	-8.8	13	
14			Rs1379339597 G/A	-8.7	13	1.154177301
			Wild	-14.5	13	1.113037794

	hsa-mir-153-5p	MIMAT0026480	Rs1272097179s A/C	-14.5	13	
15			Rs1379339597 G/A	-15.3	13	1.126750963
			Wild	-8.7	12	
	hsa-mir-1277-5p	MIMAT0022724	Rs1272097179 A/C	-8.7	12	
			Rs1379339597 G/A	-7.7	15	
16			Rs1055751280 C/T	-8.7	12	1.05818512
			wild	-17.7	1	
	hsa-mir-6849-5p	MIMAT0027598	Rs1379339597 G/A	-17.6	1	
			Rs1055751280 C/T	-17.1	1	
17			Rs1447949565 C/T	-19	1	1.195316807
			Wild	-16.2	1	
	hsa-mir-3174	MIMAT0015051	Rs1379339597 G/A	-13.9	1	
			Rs1055751280 C/T	-19.4	1	
18			Rs1447949565 C/T	-14	1	1.044471951
			Wild	-12.2	11	
	hsa-mir-4693-3p	MIMAT0019785	Rs1055751280 C/T	-12.2	11	
19			Rs1447949565 C/T	-12.2	11	
	hsa-mir-892c-5p	MIMAT0025857	Wild	-16.5	4	1.044471951
20			rs1253643130 C/T	-16.5	4	
			wild	-20.4	3	
	hsa-mir-4742-3p	MIMAT0019873	rs1253643130 C/T	-20.4	3	
21			Rs1182602720 A/G	-20.4	3	1.044471951
			wild	-16.9	6	
			rs1253643130 C/T	-15.3	3	1.05818512
	hsa-mir-5001-3p	MIMAT0021022	Rs1182602720 A/G	-16.9	6	1.071898288
			rs1482628581 C/T	-16.9	6	
22			Rs1482628581 C/G	-16.9	6	
			Wild	-8.4	1	1.113037794
			rs1253643130 C/T	-8.4	1	
	hsa-mir-889-3p	MIMAT0004921	Rs1182602720 A/G	-8.9	1	
			rs1482628581 C/T	-8.2	12	
23			Rs1482628581 C/G	-11.7	11	
			Wild	-10.8	1	
			rs1253643130 C/T	-8.5	1	1.044471951
	hsa-mir-9-5p	MIMAT0000441	Rs1182602720 A/G	-10.1	1	
			rs1482628581 C/T	-10.8	1	
24			Rs1482628581 C/G	-10.8	1	
			wild	-6.8	14	
			rs1253643130 C/T	-6.8	14	
	hsa-mir-129-1-3p	MIMAT0004548	Rs1182602720 A/G	-6.8	14	1.071898288
			rs1482628581 C/T	-6.8	14	
25			Rs1482628581 C/G	-6.8	14	
			Wild	-8.8	14	
			rs1253643130 C/T	-8.8	14	
	hsa-mir-129-2-3p	MIMAT0004605	Rs1182602720 A/G	-8.8	14	1.222743144
			rs1482628581 C/T	-8.8	14	
26			Rs1482628581 C/G	-8.8	14	
			Wild	-8.2	10	
	hsa-mir-126-5p	MIMAT0000444	rs1482628581 C/T	-8.2	10	
27			Rs1482628581 C/G	-8.2	10	1.140464132
	hsa-mir-1307-5p	MIMAT0022727	Wild	-13.8	1	
28			Rs1000540686 T/C	-13.8	1	
			Wild	-6.7	1	
	hsa-mir-6813-3p	MIMAT0027527	Rs1000540686 T/C	-6.7	1	
			Rs1350500051 C/G	-10.4	10	
29			Rs998706962 C/A	-6.7	1	1.181603638
			WIld	-7.7	9	
			Rs1000540686 T/C	-6.8	1	
			Rs1350500051 C/G	-11.8	9	
	hsa-mir-7157-5p	MIMAT0028224	Rs998706962 C/A	-8.9	9	
			Rs965329334 C/A	-8	9	
			Rs965329334 C/T	-7.8	22	
30			Rs965329334 C/G	-8.7	9	
			wild	-12.6	16	1.250169481
			Rs1000540686 T/C	-12.6	16	
			Rs1350500051 C/G	-13.4	4	
	hsa-mir-324-5p	MIMAT0000761	Rs998706962 C/A	-11.4	17	
			Rs965329334 C/A	-12.6	16	
			Rs965329334 C/T	-13.1	16	

31			Rs965329334 C/G	-12.6	16	
			Wild	-9	17	
			Rs998706962 C/A	-9	17	
	hsa-mir-2277-3p	MIMAT0011777	Rs965329334 C/A	-9.2	13	1.099324626
			Rs965329334 C/T	-9	17	
			Rs965329334 C/G	-11.9	3	
32			Rs1229558462 C/A	-8.3	3	
			wild	-9.7	14	
			Rs998706962 C/A	-9.8	2	
	hsa-mir-1256	MIMAT0005907	Rs965329334 C/A	-9.7	14	
			Rs965329334 C/T	-9.7	14	
			Rs965329334 C/G	-9.7	14	
33			Rs1229558462 C/A	-10.6	14	
			wild	-12.8	9	1.044471951
			Rs965329334 C/A	-12.8	9	
			Rs965329334 C/T	-12.8	9	
	hsa-mir-635	MIMAT0003305	Rs965329334 C/G	-12.8	9	
			Rs1229558462 C/A	-10.8	9	
			Rs1221104800 T/C	-12.8	9	
34			Rs1293200978 C/A	-13.1	9	
			Wild	-14.8	10	
			Rs965329334 C/A	-14.8	10	
			Rs965329334 C/T	-14.8	10	
			Rs965329334 C/G	-14.9	3	
	hsa-mir-668-3p	MIMAT0003881	Rs1229558462 C/A	-14.8	10	
			Rs1221104800 T/C	-17.5	10	1.071898288
			Rs1293200978 C/G	-19.3	5	
			Rs543265725 A/T	-16.6	10	
35			Rs931148603 G/C	-17	5	
			wild	-16.5	11	
			Rs965329334 C/A	-16.5	11	
			Rs965329334 C/T	-16.5	11	
			Rs965329334 C/G	-16.5	11	
	hsa-mir-597-5p	MIMAT0003265	Rs1229558462 C/A	-16.5	11	
			Rs1221104800 T/C	-12.3	11	
			Rs1293200978 C/G	-15.3	9	
			Rs543265725 A/T	-15.8	11	
			Rs931148603 G/C	-14.9	11	
36			Rs1405481375 A/G	-15.5	11	
			Wild	-23.7	6	1.044471951
			Rs1221104800 T/C	-23.4	6	
			Rs1293200978 C/G	-24.8	6	
			Rs543265725 A/T	-19.8	6	
			Rs931148603 G/C	-20	6	
	hsa-mir-6847-3p	MIMAT0027595	Rs1405481375 A/G	-23	6	
			Rs1446033604 C/A	-25.5	6	
			Rs1446033604 C/T	-22.2	6	
			Rs985257000 C/G	-23.7	6	
			Rs985257000 C/T	-23.7	6	
37			rs1290873009 T/A	-23.7	6	
			Wild	-22.7	1	
			Rs1221104800 T/C	-20.7	3	1.332448493
			Rs1293200978 C/G	-20.7	4	
			Rs543265725 A/T	-25.5	1	
			Rs931148603 G/C	-25.5	1	
			Rs1405481375 A/G	-23.3	1	
	hsa-mir-1301-3p	MIMAT0005797	Rs1446033604 C/A	-25.7	1	
			Rs1446033604 C/T	-26.7	1	
			Rs985257000 C/G	-23.1	1	
			Rs985257000 C/T	-22.8	1	
			rs1290873009 T/A	-22.7	1	
			rs1433602120 A/G	-22.8	1	1.085611457
38			rs938002222 T/A	-22.7	1	
			Wild	-19.8	3	
			Rs1221104800 T/C			
			Rs1293200978 C/G	-22.6	2	
			Rs543265725 A/T	-19.1	4	
			Rs931148603 G/C	-17.9	5	
			Rs1405481375 A/G	-19.1	3	

			Rs1446033604 C/A	-19.1	3	
	hsa-let-7f-2-3p	MIMAT0004487	Rs1446033604 C/T	-19.1	3	
			Rs985257000 C/G	-22	6	1.126750963
			Rs985257000 C/T	-19.1	3	
			rs1290873009 T/A	-20.8	3	
			rs1433602120 A/G	-19.7	3	
			rs938002222 T/A	-19.3	3	
			rs992016379 C/A	-20.1	3	
39			rs992016379 C/T	-19.7	3	
			wild	-11.5	1	
			Rs931148603 G/C	-10.5	1	
			Rs1405481375 A/T	-12.8	1	1.071898288
			Rs1446033604 C/A	-12.8	5	
			Rs1446033604 C/T	-14	1	
			Rs985257000 C/G	-9.2	1	
	hsa-mir-539-3p	MIMAT0022705	Rs985257000 C/T	-11.4	5	
			rs1290873009 T/A	-9.9	12	1.26388265
			rs1433602120 A/G	-12.9	1	
			rs938002222 T/A	-7.8	13	
			rs992016379 C/A	-14.5	1	
			rs992016379 C/T	-12.8	1	
40			rs1465811840 G/T	-11.5	1	
			Wild	-11.3	8	1.373587999
			rs1259533182 A/G	-12.8	1	
			Rs981682780 T/C	-11.3	8	
	hsa-mir-4531	MIMAT0019070	Rs687914 G/A/T	-9.5	3	
			Rs687914 G/A/T	-11.9	3	
41			Rs1558526560 G/-	-11.3	3	
			wild	-11.8	1	
			rs1259533182 A/G	-12.1	1	
			Rs981682780 T/C	-12.8	3	1.140464132
			Rs687914 G/A/T	-10.8	1	
	hsa-mir-4790-3p	MIMAT0019961	Rs687914 G/A/T	-12.4	1	
			Rs1558526560 G/-	-10.8	1	
			rs902664279 T/A	-11.1	1	
			Rs1224086295 C/G/T	-11.8	1	
42			Rs1224086295 C/G/T	-11.8	1	
			Wild	-15.4	2	1.250169481
			Rs981682780 T/C	-15.7	2	
			Rs687914 G/A/T	-14.3	2	
			Rs687914 G/A/T	-13	9	
	hsa-mir-3614-5p	MIMAT0017992	Rs1558526560 G/-	-14.1	2	
			rs902664279 T/A	-12.6	2	
			Rs1224086295 C/G/T	-18.5	2	
			Rs1224086295 C/G/T	-15.7	2	1.071898288
			Rs1261920018 G/A/C …	…		
43			Rs1261920018 G/A/C …	…		
			wild	-18.3	5	
			rs902664279 T/A	-16.4	3	
			Rs1224086295 C/G/T	-22.1	7	
	hsa-mir-4732-5p	MIMAT0019855	Rs1224086295 C/G/T	-22.1	5	1.113037794
			Rs1261920018 G/A/C	-16.7	5	
			Rs1261920018 G/A/C	-16.3	2	
44			Rs1000988694 C/T	-22	5	
			wild	-18.9	8	
			rs902664279 T/A	-18.4	9	
			Rs1224086295 C/G/T	-16.4	1	
	hsa-mir-6831-5p	MIMAT0027562	Rs1224086295 C/G/T	-17.2	8	
			Rs1261920018 G/A/C	-19.3	8	1.05818512
			Rs1261920018 G/A/C	-14.7	1	
45			Rs1000988694 C/T	-21.9	8	
			Wild	-16.1	3	
			rs902664279 T/A	-17.3	3	
			Rs1224086295 C/G/T	-13.2	7	
	hsa-mir-4650-3p	MIMAT0019714	Rs1224086295 C/G/T	-13.4	10	
			Rs1261920018 G/A/C	-15.8	3	1.085611457
			Rs1261920018 G/A/C	-14.4	3	
46			Rs1000988694 C/T	-14.8	3	
			Wild	-9.7	14	

			rs902664279 T/A	-9.7	14	
			Rs1224086295 C/G/T	-9.7	14	
	hsa-mir-3927-3p	MIMAT0018202	Rs1224086295 C/G/T	-10.4	2	
			Rs1261920018 G/A/C	-8.3	8	1.126750963
			Rs1261920018 G/A/C	-7.9	1	
47			Rs1000988694 C/T	-9.8	1	
			Wild	-8.8	3	
			rs902664279 T/A	-10.5	1	
			Rs1224086295 C/G/T	-8.4	13	
	hsa-mir-98-5p	MIMAT0000096	Rs1224086295 C/G/T	-8.4	13	
			Rs1261920018 G/A/C	-8.8	3	
			Rs1261920018 G/A/C	-8.8	3	
48			Rs1000988694 C/T	-9.4	13	
			Wild	-9.3	5	
			rs902664279 T/A	-12.2	1	
			Rs1224086295 C/G/T	-9.6	3	1.044471951
	hsa-let-7c-5p	MIMAT0000064	Rs1224086295 C/G/T	-11.1	3	
			Rs1261920018 G/A/C	-9	5	
			Rs1261920018 G/A/C	-8.8	3	
49			Rs1000988694 C/T	-12.2	5	
			Wild	-23.9	3	
			rs902664279 T/A	-24	4	
			Rs1224086295 C/G/T	-22.6	8	
	hsa-mir-4669	MIMAT0019749	Rs1224086295 C/G/T	-22.6	8	
			Rs1261920018 G/A/C	-23.1	6	
			Rs1261920018 G/A/C	-23	3	
50			Rs1000988694 C/T	-23.4	6	
			wild	-13.5	1	1.113037794
			Rs1224086295 C/G/T	-15.5	1	
	hsa-mir-124-5p	MIMAT0004591	Rs1224086295 C/G/T	-13	6	
			Rs1261920018 G/A/C	-10.8	11	
			Rs1261920018 G/A/C	-10.8	11	
51			Rs1000988694 C/T	-14.3	13	
			wild	-6	15	
	hsa-mir-5010-3p	MIMAT0021044	Rs1261920018 G/A/C	-6	5	
			Rs1261920018 G/A/C	-6	15	
52			Rs1000988694 C/T	-6	5	1.442153843
			wild	-6.6	15	
	hsa-mir-599	MIMAT0003267	Rs1261920018 G/A/C	-6.6	15	
			Rs1261920018 G/A/C	-6.6	15	
53			Rs1000988694 C/T	-6	5	
			Wild	-6.8	4	
	hsa-mir-6782-3p	MIMAT0027465	Rs1261920018 G/A/C	-6.2	17	
54			Rs1000988694 C/T	-7.5	4	
			Wild	-16.7	1	
	hsa-mir-3140-3p	MIMAT0015008	Rs1261920018 G/A/C	-16.4	1	
			Rs1261920018 G/A/C	-15.2	3	1.167890469
55			Rs1000988694 C/T	-17.5	1	
	hsa-mir-1915-5p	MIMAT0007891	Wild	-12.5	1	
56			Rs1000988694 C/T	-12.2	11	
	hsa-mir-1238-3p	MIMAT0005593	Wild	..	..	
57			NO SNP	..	..	
	hsa-mir-6764-5p	MIMAT0027428	Wild	-12.9	1	
58			Rs1390200563 C/G	-13	1	
	hsa-mir-1915-3p	MIMAT0007892	wild	-25.1	1	
59			Rs1390200563 C/G	-28.6	1	1.277595819
	hsa-mir-652-5p	MIMAT0022709	wild	-18.4	2	
60			Rs1390200563 C/G	-20.6	2	
			wild	-21.7	2	
	hsa-mir-769-3p	MIMAT0003887	Rs1390200563 C/G	-19.4	2	
61			Rs1426258125 A/T	-21.7	2	
			Wild	-14.7	1	
	hsa-mir-4663	MIMAT0019735	Rs1390200563 C/G	-16.9	1	
			Rs1426258125 A/T	-14.7	1	
62			Rs1261533756 C/G	-14.7	1	1.044471951
			Wild	-14.9	1	
	hsa-mir-185-3p	MIMAT0004611	Rs1426258125 A/T	-14.9	1	
63			Rs1261533756 C/G	-17.2	1	
			wild	-10	1	

	hsa-mir-3160-3p	MIMAT0015034	Rs1426258125 A/T	-10	1	
64			Rs1261533756 C/G	-10.7	2	
			wild	-16.5	1	
	hsa-mir-939-5p	MIMAT0004982	Rs1426258125 A/T	-16.5	1	1.071898288
65			Rs1261533756 C/G	-12.7	1	
			wild	-16	1	
	hsa-mir-6726-5p	MIMAT0027353	Rs1426258125 A/T	-16	1	
66			Rs1261533756 C/G	-12.2	1	
			wild	-12.5	8	
	hsa-mir-5591-5p	MIMAT0022301	Rs1426258125 A/T	-12.5	8	
67			Rs1261533756 C/G	-10.1	8	
			wild	-17.4	1	
	hsa-mir-6871-5p	MIMAT0027642	Rs1426258125 A/T	-18.5	3	
68			Rs1261533756 C/G	-18.2	4	1.085611457
			wild	-13.5	1	
	hsa-mir-920	MIMAT0004970	Rs1426258125 A/T	-13.5	1	
69			Rs1261533756 C/G	-9.7	1	
			Wild	-20.3	1	
	hsa-mir-637	MIMAT0003307	Rs1426258125 A/T	-19.3	1	
70			Rs1261533756 C/G	-16.5	1	
			Wild	-19.2	3	
	hsa-mir-3192-5p	MIMAT0015076	Rs1426258125 A/T	-16.7	5	
71			Rs1261533756 C/G	-14.5	3	1.071898288
			Wild	-12.5	5	
	hsa-mir-4300	MIMAT0016853	Rs1426258125 A/T	-12.5	5	
72			Rs1261533756 C/G	-10.6	8	
			Wild	-13.3	3	
	hsa-mir-1298-3p	MIMAT0026641	Rs1426258125 A/T	-13.2	3	
73			Rs1261533756 C/G	-17.4	4	
			Wild	-12.6	1	
	hsa-mir-4314	MIMAT0016868	Rs1426258125 A/T	-10.7	1	1.126750963
74			Rs1261533756 C/G	-13.3	1	
			Wild	-10.9	6	
	hsa-mir-3153	MIMAT0015026	Rs774739390 C/T	-9	6	
			RS1208941692 G/A	-10.9	6	
75			Rs1344558376 G/A	-10.9	6	1.085611457
			Wild	-8.6	10	
	hsa-mir-4311	MIMAT0016863	Rs774739390 C/T	-7.8	4	
			RS1208941692 G/A	-9.3	10	
76			Rs1344558376 G/A	-8.6	10	1.113037794
			Wild	-9.7	1	
	hsa-mir-6733-5p	MIMAT0027367	Rs774739390 C/T	-10.1	1	
			RS1208941692 G/A	-9.7	1	
77			Rs1344558376 G/A	-9.7	1	1.167890469
			Wild	-10.9	5	
	hsa-mir-4490	MIMAT0019025	Rs774739390 C/T	-8.7	5	
			RS1208941692 G/A	-11.6	5	
78			Rs1344558376 G/A	-10.9	5	1.26388265
			Wild	-11.5	6	1.071898288
	hsa-mir-204-3p	MIMAT0022693	Rs774739390 C/T	-9.8	7	1.250169481
			RS1208941692 G/A	-10.4	6	1.113037794
79			Rs1344558376 G/A	-10.8	6	1.05818512
			wild	-15.1	1	1.085611457
	hsa-mir-3978	MIMAT0019363	Rs774739390 C/T	-16.2	1	1.099324626
			RS1208941692 G/A	-15.8	1	
80			Rs1344558376 G/A	-15.1	1	
			wild	-8	6	
	hsa-mir-6739-5p	MIMAT0027379	Rs774739390 C/T	-9.2	6	
			RS1208941692 G/A	-8	6	1.167890469
81			Rs1344558376 G/A	-8	6	
			wild	-10.5	1	
	hsa-mir-4646-5p	MIMAT0019707	Rs774739390 C/T	-8.8	1	
			RS1208941692 G/A	-10.9	5	
82			Rs1344558376 G/A	-10.5	1	1.044471951
			wild	-12.2	4	
	hsa-mir-127-5p	MIMAT0004604	Rs774739390 C/T	-9.4	4	
			RS1208941692 G/A	-12.9	4	
83			Rs1344558376 G/A	-12.2	4	1.126750963
			Wild	-13	2	

84	hsa-mir-875-3p	MIMAT0004923	Rs774739390 C/T	-11.2	2	
			RS1208941692 G/A	-14.1	2	
			Rs1344558376 G/A	-11.7	2	1.209029975
			Wild	-12.7	1	
85	hsa-mir-4738-3p	MIMAT0019867	Rs774739390 C/T	-10.9	12	
			RS1208941692 G/A	-13.4	1	1.318735325
			Rs1344558376 G/A	-12.7	1	
			Wild	-11.6	1	
86	hsa-mir-320e	MIMAT0015072	Rs774739390 C/T	-9.8	1	1.373587999
			RS1208941692 G/A	-12.4	1	
			Rs1344558376 G/A	-11.6	1	
			Wild	-9.5	1	1.044471951
87	hsa-mir-6812-5p	MIMAT0027524	Rs774739390 C/T	-7.6	1	
			RS1208941692 G/A	-9.5	1	
			Rs1344558376 G/A	-9.5	1	
			wild	-11.8	3	1.140464132
88	hsa-mir-8082	MIMAT0031009	Rs774739390 C/T	-12.6	8	
			RS1208941692 G/A	-13.5	3	
			Rs1344558376 G/A	-11.8	3	
			Wild	-8.6	1	1.113037794
89	hsa-mir-1255b-5p	MIMAT0005945	Rs774739390 C/T	-8.4	18	
			RS1208941692 G/A	-8.4	18	
			Rs1344558376 G/A	-7.5	1	
			Wild	-10	16	1.209029975
90	hsa-mir-1255a	MIMAT0005906	Rs774739390 C/T	-10	16	
			RS1208941692 G/A	-10	16	
			Rs1344558376 G/A	-10	16	
			wild	-15	2	1.222743144
91	hsa-mir-651-5p	MIMAT0003321	Rs774739390 C/T	-15	2	
			RS1208941692 G/A	-15	3	
			Rs1344558376 G/A	-14.2	3	
92	hsa-mir-708-5p	MIMAT0004926	wild	-18.7	1	1.236456313
			Rs1344558376 G/A	-17.6	1	
93	hsa-mir-4471	MIMAT0018998	wild	-11.5	5	
			Rs1344558376 G/A	-11.5	5	
94	hsa-mir-548o-3p	MIMAT0005919	wild	-9.4	8	1.181603638
			rs1475822634 C/G	-9.1	8	
95	hsa-mir-548ah-3p	MIMAT0020957	wild	-9.4	8	
			rs1475822634 C/G	-9.1	8	
96	hsa-mir-548am-3p	MIMAT0019076	wild	-8.5	8	1.05818512
			rs1475822634 C/G	-8.2	8	
97	hsa-mir-548j-3p	MIMAT0026737	wild	-9.9	8	
			rs1475822634 C/G	-9.6	8	
98	hsa-mir-6768-5p	MIMAT0027436	wild	-20	8	1.195316807
			rs1475822634 C/G	-19.8	4	
			Rs918001784 G/A	-18.7	4	
99	hsa-mir-539-3p	MIMAT0022705	Wild	-7.1	4	
			rs1475822634 C/G	-8.3	9	
			Rs918001784 G/A	-6.8	1	1.154177301
100	hsa-mir-377-3p	MIMAT0000730	Wild	-10.6	1	
			rs1475822634 C/G	-12.6	7	
			Rs918001784 G/A	-10.6	1	
101	hsa-mir-342-3p	MIMAT0000753	Wild	-13.5	5	
			rs1475822634 C/G	-12.5	3	1.113037794
			Rs918001784 G/A	-13.5	5	
102	hsa-mir-3680-3p	MIMAT0018107	Wild	-14.8	6	
			rs1475822634 C/G	-14.8	6	
			Rs918001784 G/A	-14.8	6	
103	hsa-mir-4776-3p	MIMAT0019933	Wild	-18.7	4	1.05818512
			rs1475822634 C/G	-18.7	4	
			Rs918001784 G/A	-18.1	11	
104	hsa-mir-6501-5p	MIMAT0025458	Wild	-19	1	
			Rs918001784 G/A	-18	1	
105	hsa-mir-4717-3p	MIMAT0019830	Wild	-11.4	5	1.167890469
			Rs1270066804 G/T	-12.7	5	
106	hsa-mir-633	MIMAT0003303	Wild	-13.6	2	
			Rs1270066804 G/T	-13.4	9	
107	hsa-mir-3129-5p	MIMAT0014992	Wild	-8.5	2	
			Rs1270066804 G/T	-10.7	2	1.044471951

	hsa-mir-199a-3p	MIMAT0000232	Wild	-19.9	1	
108			Rs1270066804 G/T	-21.9		
	hsa-mir-199b-3p	MIMAT0004563	Wild	-19.9	1	
109			Rs1270066804 G/T	-21.9	1	1.318735325
	hsa-mir-6866-3p	MIMAT0027633	Wild	-20.9	7	
110			Rs1270066804 G/T	-16	1	
	hsa-mir-188-5p	MIMAT0000457	Wild	-15.2	6	
111			Rs1270066804 G/T	-15.2	6	
	hsa-mir-3143	MIMAT0015012	Wild	-16.1	4	1.071898288
112			Rs1443936062 G/A	-15.4	4	
	hsa-mir-429	MIMAT0001536	Wild	-21	1	
113			Rs1443936062 G/A	-19.8	1	
	hsa-mir-1208	MIMAT0005873	wild	-13.6	7	
114			Rs1443936062 G/A	-11.3	5	
			wild	-14.6	1	1.154177301
	hsa-mir-186-5p	MIMAT0000456	Rs1443936062 G/A	-14.5	1	
			rs986017109 A/C	-14.6	1	
115			rs986017109 A/T	-14.6	1	
			wild	-20.7	1	
	hsa-mir-6507-5p	MIMAT0025470	Rs1443936062 G/A	-18.6	1	1.222743144
			rs986017109 A/C	-19.8	1	
116			rs986017109 A/T	-19	1	
			wild	-20.4	2	
			Rs1443936062 G/A	-20.5	4	
			rs986017109 A/C	-22.9	2	1.099324626
	hsa-mir-1270	MIMAT0005924	rs986017109 A/T	-21.7	2	
			rs773211026 C/A	-20.4	2	
			rs773211026 C/T	-20.4	2	
117			rs772170785 C/T	-21.1	2	
			Wild	-148	2	1.195316807
			Rs1443936062 G/A	-15	1	
	hsa-mir-4712-3p	MIMAT0019819	rs986017109 A/C	-12.5	2	
			rs986017109 A/T	-13.6	2	
118			rs772170785 C/T	-14.8	2	
			Wild	-16.7	4	
			Rs1443936062 G/A	-16.7	2	1.05818512
			rs986017109 A/C	-14	2	
	hsa-mir-3662	MIMAT0018083	rs986017109 A/T	-14.9	2	
			rs772170785 C/T	-16.7	2	
			rs773211026 C/A	-16.7	2	
119			rs773211026 C/T	-16.7	2	1.044471951
			Wild	-18.7	1	
			Rs1443936062 G/A	-18.7	1	
	hsa-mir-3646	MIMAT0018065	rs986017109 A/C	-15.2	1	
			rs986017109 A/T	-16.4	1	
			Rs1259533133 G/A	-18.7	1	1.085611457
120			rs772170785 C/T	-18.7	1	
			Wild	-13.2	5	
			Rs1443936062 G/A	-13.2	5	
	hsa-mir-4694-3p	MIMAT0019787	rs986017109 A/C	-15.6	5	
			rs986017109 A/T	-13.2	6	1.305022156
			Rs1259533133 G/A	-11.7	5	
121			rs772170785 C/T	-11.6	2	
			wild	-20.8	1	
			rs986017109 A/C	-21.4	1	1.071898288
			rs986017109 A/T	-19.8	1	
	hsa-mir-182-5p	MIMAT0000259	Rs1443936062 G/A	-19.3	4	
			Rs1259533133 G/A	-18.4	1	
			rs772170785 C/T	-20.8	1	1.222743144
			rs773211026 C/A	-18.4	1	
122			rs773211026 C/T	-19.1	1	1.181603638
			wild	-20.5	1	
			rs986017109 A/C	-19.7	1	1.318735325
			rs986017109 A/T	-21.2	1	1.044471951
	hsa-mir-5093	MIMAT0021085	Rs1443936062 G/A	-18	2	1.044471951
			Rs1259533133 G/A	-20.5	1	1.05818512
			rs772170785 C/T	-17.8	1	1.113037794
			rs773211026 C/A	-20.5	1	
123			rs773211026 C/T	-20.5	1	1.085611457

			wild	-17.4	7	
			rs986017109 A/C	-14.2	1	1.305022156
			rs986017109 A/T	-14.4	7	
	hsa-mir-362-5p	MIMAT0000705	Rs1259533133 G/A	-17.7	7	1.195316807
			rs772170785 C/T	-15.2	13	
			rs773211026 C/A	-15.1	7	1.044471951
124			rs773211026 C/T	-17.1	7	
			Wild	-16.1	4	1.195316807
			rs986017109 A/C	-16.5	5	
			rs986017109 A/T	-14.9	12	1.140464132
	hsa-mir-3189-3p	MIMAT0015071	Rs1259533133 G/A	-15.8	4	1.250169481
			rs772170785 C/T	-16.1	4	1.071898288
			rs773211026 C/A	-13.1	4	1.154177301
125			rs773211026 C/T	-13.1	4	1.26388265
			Wild	-17.7	2	1.26388265
			rs986017109 A/C	-19.8	6	1.277595819
			rs986017109 A/T	-20.2	2	1.318735325
	hsa-mir-4751	MIMAT0019888	Rs1259533133 G/A	-15.7	2	1.140464132
			rs772170785 C/T	-18.4	8	1.05818512
			rs773211026 C/A	-19.7	2	1.071898288
126			rs773211026 C/T	-18.7	2	1.250169481
			Wild	-14.7	6	
			rs986017109 A/C	-14.5	6	
			rs986017109 A/T	-14.6	6	1.085611457
	hsa-mir-3925-5p	MIMAT0018200	Rs1259533133 G/A	-15.1	6	
			rs772170785 C/T	-13.5	1	
			rs773211026 C/A	-14.7	9	1.085611457
127			rs773211026 C/T	-17	6	
			Wild	-19.6	12	
			rs986017109 A/C	-19.6	13	
			rs986017109 A/T	-19.6	13	
	hsa-mir-589-5p	MIMAT0004799	Rs1259533133 G/A	-19.6	13	
			rs772170785 C/T	-19.6	13	1.140464132
			rs773211026 C/A	-19.8	13	
128			rs773211026 C/T	-19.4	13	
			wild	-15.5	2	
			rs986017109 A/C	-15.5	3	1.071898288
			rs986017109 A/T	-15.5	3	
	hsa-mir-3919	MIMAT0018193	Rs1259533133 G/A	-14.4	3	
			rs772170785 C/T	-14	2	
			rs773211026 C/A	-15.5	3	
129			rs773211026 C/T	-17.5	3	
			wild	-13.4	8	1.167890469
			Rs1259533133 G/A	-13.4	9	
	hsa-mir-4717-5p	MIMAT0019829	rs772170785 C/T	-13.4	9	
			rs773211026 C/A	-17.4	3	
130			rs773211026 C/T	-13.4	9	
			wild	-20.2	1	1.332448493
			Rs1259533133 G/A	-16.7	5	
	hsa-mir-3922-5p	MIMAT0019227	rs772170785 C/T	-16.7	5	
			rs773211026 C/A	-15.4	2	
131			rs773211026 C/T	-17.2	2	
			Wild	-8.6	4	1.099324626
	hsa-mir-1277-5p	MIMAT0022724	rs753172025 C/T	-8.6	4	
132			rs371492921 C/T	-8.6	4	
			Wild	-10.2	8	
	hsa-mir-5580-3p	MIMAT0022274	rs753172025 C/T	-10.2	8	
133			rs371492921 C/T	-10.2	8	
			Wild	-13.3	2	
	hsa-mir-494-3p	MIMAT0002816	rs371492921 C/T	-13.3	2	1.181603638
134			rs753172025 C/T	-13.3	2	
			Wild	-23.8	7	
	hsa-mir-4646-5p	MIMAT0019707	rs371492921 C/T	-24.7	7	
			rs753172025 C/T	-21.5	7	
135			rs750009110 C/G	-19.8	7	
			Wild	-14.3	1	
	hsa-mir-6733-5p	MIMAT0027367	rs371492921 C/T	-14.7	1	1.05818512
136			rs753172025 C/T	-13.5	1	
			Wild	-19.7	7	

			Rs755680404 C/G	-19.7	7	
	hsa-mir-4667-5p	MIMAT0019743	rs750009110 C/G	-19.7	7	
			rs371492921 C/T	-16.7	7	
137			rs753172025 C/T	-17.1	12	1.085611457
			Wild	-19	1	
			Rs755680404 C/G	-19	1	
			Rs201815286 C/T	-19	1	
	hsa-mir-330-5p	MIMAT0004693	Rs753516084 G/A	-19	1	
			rs750009110 C/G	-19	1	
			rs371492921 C/T	-19.4	1	1.113037794
138			rs753172025 C/T	-17.3	1	
			Wild	-21.1	4	
			Rs755680404 C/G	-21.1	4	
	hsa-mir-204-3p	MIMAT0022693	rs750009110 C/G	-21.9	4	
			rs753172025 C/T	-22.1	4	
139			rs371492921 C/T	-19.7	4	1.291308987
			Wild	-12.5	6	
	hsa-mir-4261	MIMAT0016890	rs371492921 C/T	-13.1	6	
140			rs753172025 C/T	-13.8	2	
			Wild	-15.2	5	
			Rs755680404 C/G	-15.2	5	
			Rs201815286 C/T	-15.2	5	1.044471951
	hsa-mir-326	MIMAT0000756	Rs753516084 G/A	-15.2	5	
			rs750009110 C/G	-15.2	5	
			rs753172025 C/T	-13.5	5	
141			rs371492921 C/T	-15.6	5	
			Wild	-17.1	1	
			Rs755680404 C/G	-17.1	1	1.113037794
	hsa-mir-4314	MIMAT0016868	Rs201815286 C/T	-17.1	1	
			rs750009110 C/G	-17.1	1	
			rs753172025 C/T	-14.6	1	
142			rs371492921 C/T	-16.4	1	
			Wild	-7	3	
			Rs755680404 C/G	-8.5	15	1.126750963
	hsa-mir-6821-3p	MIMAT0027543	Rs201815286 C/T	-7.2	16	
			Rs753516084 G/A	-7	3	
			Rs1407190036 C/A	-7	3	
143			Rs754742928 C/A	-7.4	17	1.099324626
			Wild	-13.9	6	
			Rs755680404 C/G	-14	4	
			Rs201815286 C/T	-13.6	6	
	hsa-mir-548ag	MIMAT0018969	Rs753516084 G/A	-14	4	1.167890469
			Rs1407190036 C/A	-13.6	6	
			Rs754742928 C/A	-16.5	6	1.126750963
			Rs900931286 C/T	-13.9	6	
144			Rs893058632 G/C	-12.5	4	1.428440674
			Wild	-15.4	1	
			Rs755680404 C/G	-14.6	2	1.071898288
			Rs201815286 C/T	-17.4	1	
	hsa-mir-6509-5p	MIMAT0025474	Rs753516084 G/A	-15.8	1	
			Rs1407190036 C/A	-13.4	1	1.044471951
			Rs754742928 C/A	-15.4	1	
			Rs900931286 C/T	-15.2	2	1.099324626
145			Rs893058632 G/C	-15.5	1	
			Wild	-8.4	12	
			Rs1407190036 C/A	-8.5	12	
	hsa-mir-545-5p	MIMAT0004785	Rs754742928 C/A	-8.5	12	1.044471951
			Rs900931286 C/T	-8.5	12	
146			Rs893058632 G/C	-8.5	12	

Table 6b: PRKCE 5'UTR variation modulating the 5'UTR interaction with regulatory microRNAs

Variation	miRNA interaction	
	**Decreased**	**Increased**
**rs1224086295 C>G**	hsa-mir-3614-5p<br>hsa-mir-4732-5p<br>hsa-mir-3927-3p<br>hsa-let-7c-5p<br>hsa-mir-124-5p	hsa-mir-6831-5p<br>hsa-mir-4650-3p<br>hsa-mir-98-5p<br>hsa-mir-4669
**rs687914 G>A**		hsa-mir-4790-3p<br>hsa-mir-4531
**rs981682780 T/C**	hsa-mir-4790-3p<br>hsa-mir-3614-5p	
**rs1000988694 C/T**	hsa-mir-4732-5p<br>hsa-mir-6831-5p<br>hsa-mir-3927-3p<br>hsa-mir-98-5p<br>hsa-let-7c-5p<br>hsa-mir-124-5p	hsa-mir-4650-3p<br>hsa-mir-4669
**rs1290873009 T/A**	hsa-let-7f-2-3p	hsa-mir-539-3p
**rs1221104800 T/C**	hsa-mir-668-3p	hsa-mir-597-5p<br>hsa-mir-6847-3p<br>hsa-mir-1301-3p
**rs1261920018 G/A**	hsa-mir-6831-5p	hsa-mir-4732-5p<br>hsa-mir-4650-3p<br>hsa-mir-3927-3p<br>hsa-let-7c-5p<br>hsa-mir-4669<br>hsa-mir-124-5p
**rs1446033604 C/A**	hsa-mir-6847-3p<br>hsa-mir-1301-3p<br>hsa-mir-539-3p	hsa-let-7f-2-3p
**rs1446033604 C/T**	hsa-mir-1301-3p<br>hsa-mir-539-3p	hsa-mir-6847-3p<br>hsa-let-7f-2-3p

Variation	miRNA interaction	
	**Decreased**	**Increased**
**rs1224086295 C>T**	hsa-mir-3614-5p<br>hsa-mir-4732-5p<br>hsa-let-7c-5p	hsa-mir-6831-5p<br>hsa-mir-4650-3p<br>hsa-mir-98-5p<br>hsa-mir-4669<br>hsa-mir-124-5p
**rs687914 G>T**	hsa-mir-4790-3p<br>hsa-mir-4531	hsa-mir-3614-5p
**rs1259533182 A/G**	hsa-mir-4531<br>hsa-mir-4790-3p	
**rs1261920018 G/C**		hsa-mir-4732-5p<br>hsa-mir-6831-5p<br>hsa-mir-4650-3p<br>hsa-mir-3927-3p<br>hsa-let-7c-5p<br>hsa-mir-4669<br>hsa-mir-124-5p
**rs1293200978 C/G**	hsa-mir-668-3p<br>hsa-mir-6847-3p<br>hsa-let-7f-2-3p	hsa-mir-597-5p<br>hsa-mir-1301-3p
**rs1405481375 A/G**	hsa-mir-1301-3p	hsa-mir-597-5p<br>hsa-mir-6847-3p<br>hsa-let-7f-2-3p
**rs1405481375 A/T**	hsa-mir-539-3p	
**rs1433602120 A/G**	hsa-mir-1301-3p<br>hsa-mir-539-3p	hsa-let-7f-2-3p
**rs1558526560 G/-**		hsa-mir-4531<br>hsa-mir-4790-3p<br>hsa-mir-3614-5p
**rs981682780 T/C**		hsa-mir-4531

**rs543265725 A/T**	hsa-mir-668-3p hsa-mir-6847-3p hsa-mir-1301-3p hsa-let-7f-2-3p hsa-mir-597-5p	**rs938002222 T/A**	hsa-let-7f-2-3p hsa-mir-539-3p
		**rs965329334 C/G**	hsa-mir-668-3p
**rs902664279 T/A**	hsa-mir-4650-3p hsa-mir-4790-3p hsa-mir-98-5p hsa-mir-3614-5p hsa-let-7c-5p hsa-mir-4732-5p hsa-mir-4669 hsa-mir-6831-5p	**rs992016379 C/A**	hsa-let-7f-2-3p hsa-mir-539-3p
		**rs992016379 C/T**	hsa-let-7f-2-3p hsa-mir-539-3p
**rs931148603 G/C**	hsa-mir-668-3p hsa-mir-597-5p hsa-mir-1301-3p hsa-mir-6847-3p hsa-let-7f-2-3p hsa-mir-539-3p	**rs985257000 C/T**	hsa-mir-1301-3p hsa-let-7f-2-3p hsa-mir-539-3p
		**rs985257000 C/G**	hsa-mir-1301-3p hsa-mir-539-3p hsa-let-7f-2-3p
