# Supplementary material for: PRKCE non-coding variants influence on transcription as well as translation of its gene
Source: RNA Biol. 2022 Oct 26;19(1):1115–29. doi: 10.1080/15476286.2022.2139110 (PMC9621080; doi:10.1080/15476286.2022.2139110)
Supplement: Supplemental Material [file KRNB_A_2139110_SM6803.zip › Supplementary table 3 .pdf]

Table 3: List of PRKCE filtered 5' and 3' UTRs along with the RegulomeDB score and Rank

5' UTR

Variant ID	RegulomeDB		Alleles	MAF		Allele frequency		GREP	CADD
	Rank	Score		Ancestral	Altered	Ancestral	Altered		
rs569884823	3a	0.8507	C/G	< 0.01	**0.01**	1	6.57E-06	-0.82	0.611
rs1227344174	3a	1	T/G		**< 0.01**	1	1.31E-05	-0.22	6.692
rs1302848957	3a	0.9826	C/T		**< 0.01**	1	0.00E+00	0.24	3.807
rs946217897	3a	0.9826	C/G		**< 0.01**	1	1.97E-05	-2.43	0.595
rs946217897	3a	0.9826	C/T		**< 0.01**	1	0.00E+00	-2.43	0.73
rs1444088897	4	0.705	G/A		**< 0.01**	1	6.64E-06	-1.86	12.94
rs1279310031	4	0.705	C/T		**< 0.01**	0.9999	7.96E-06	0.93	16.41
rs538954895	4	0.705	C/G		**0.01**	0.9999	0.000126	-0.01	14.92
rs538954895	4	0.705	C/T		**0.01**	0.9999	6.63E-06	-0.01	15.19
rs1299335294	4	0.705	G/A		**<0.01**	1	0.00E+00	-1.2	14.03
rs1363740502	4	0.705	G/A		**<0.01**	1	0.00E+00	0.09	15.48
rs912480755	4	0.705	G/-		**<0.01**	1	0.00E+00	1.18	
rs61762789	4	0.705	G/A		**0.01**	0.998	2.00E-03	1.18	17.49
rs1226860990	4	0.705	C/A		**<0.01**	1	6.62E-06	1.18	18.28
rs558553528	4	0.705	C/G		0.01	0.999	1.00E-03	1.16	21
rs1414250098	4	0.705	G/A		**<0.01**	0.999	9.56E-05	1.18	20.2
rs1272097179	2c	0.715	A/C		**<0.01**	1	1.32E-05	-2.18	7.618
rs1379339597	2b	0.84	G/A		**<0.01**	1	6.58E-06	0.49	19.01
rs1055751280	2c	0.98	C/T		<0.01	1	6.58E-06	-2.61	15.11
rs1447949565	2c	0.76	C/T		<0.01	1	6.58E-06	0.59	18.46
rs1253643130	2b	0.8368	C/T		<0.01	1	7.96E-06	-0.42	18.73
rs1182602720	2b	0.8368	A/G		<0.01	1	1.98E-05	-0.68	18.95
rs1482628581	2b	0.8682	C/G		<0.01	1	6.57E-06	1.54	19.39
rs1482628581	2b	0.8682	C/T	-	-	1	6.57E-06	1.54	19.71
rs1000540686	4	0.705	T/C		<0.01	1	1.32E-05	0.49	19.3
rs1350500051	4	0.705	C/G		<0.01	1	6.58E-06	-2.94	14.26
rs998706962	2b	1	C/A		<0.01	1	2.63E-05	1.65	16.34
rs965329334	2b	0.7739	C/T		<0.01	0.999	1.97E-05	0.7	18.22
rs1229558462	2b	0.7088	C/A		<0.01	1	0.00E+00	1.65	18.73
rs1221104800	4	0.705	T/C		<0.01	1	6.62E-06	-3.3	13.6
rs1293200978	4	0.705	C/G		<0.01	0.999	7.96E-06	-0.24	17.21
rs543265725	4	0.705	A/T	<0.01	<0.01	0.999	7.25E-05	-1.85	18.04
rs931148603	4	0.705	G/C		<0.01	0.999	7.96E-06	0.58	17.94
rs1405481375	4	0.705	A/G		<0.01	1	1.98E-05	1.65	20.1
rs1446033604	4	0.705	C/A		<0.01	1	2.63E-05	1.47	17.88
rs985257000	4	0.705	C/G		<0.01	1	2.63E-05	1.65	18.03
rs1290873009	4	0.705	T/A		<0.01	1	6.60E-06	1.65	17.76
rs1433602120	4	0.705	A/G		<0.01	0.999	7.96E-06	-0.33	19.24
rs938002222	4	0.705	T/A		<0.01	1	6.60E-06	0.48	19.58
rs992016379	4	0.705	C/T		<0.01	1	1.32E-05	1.65	19.39
rs1465811840	4	0.705	G/T		<0.01	1	3.29E-05	0.7	18.92

rs1213772661	4	0.705	G/C	<0.01	1	6.57E-06	1.65	19.96
rs971806465	4	0.705	G/A	<0.01	1	6.57E-06	1.65	20.2
rs1259533182	4	0.705	A/G	<0.01	0.999	1.59E-05	0.52	18.27
rs981682780	4	0.705	T/C	<0.01	0.999	3.00E-04	1.65	19.05
rs687914	4	0.705	G/T	0.38	0.892	1.08E-01	0.68	17.88
rs1558526560	4	0.705	G/-	<0.01	1	6.58E-06	0.68	-
rs902664279	4	0.705	T/A	<0.01	1	1.98E-05	-0.62	17.29
rs1224086295	2b	0.7512	C/G	<0.01	1	1.98E-05	-0.19	15
rs1261920018	2b	1	G/C	<0.01	0.999	0.0002413	1.74	20.6
rs1000988694	2b	0.8682	C/T	<0.01	1	1.32E-05	1.74	20.7
rs1217420340	2b	0.7956	G/C	<0.01	0.999	1.77E-04	0.8	19.61
rs1390200563	4	0.705	C/G	<0.01	1	6.60E-06	-0.61	12.6
rs1426258125	4	0.705	A/T	<0.01	0.999	7.96E-06	1.39	20.9
rs1261533756	2b	0.8117	C/G	<0.01	1	7.12E-06	1.39	20.1
rs774739390	2b	1	C/T	<0.01	0.999	4.86E-04	1.39	19.22
rs1208941692	2b	0.8569	G/A	<0.01	1	2.01E-05	0.4	18.26
rs1344558376	2b	0.8429	G/A	<0.01	1	1.34E-05	0.45	20.7
rs1475822634	4	0.705	C/G	<0.01	1	6.57E-06	1.99	19.57
rs918001784	4	0.705	G/T	<0.01	1	3.95E-05	0.97	17.24
rs1270066804	2b	0.8682	G/T	<0.01	0.999	7.96E-06	1.81	20.5
rs1443936062	2b	0.8362	G/A	<0.01	1	6.58E-06	1.08	17.13
rs986017109	2b	0.8569	A/C	-			-1.38	18.75
rs772170785	2b	0.7319	C/T	<0.01	1	6.02E-06	2.09	20.8
rs1259533133	2b	0.8285	G/A	<0.01	1	0.00E+00	1.16	21.1
rs773211026	2b	0.7614	C/A	<0.01	1	1.84E-06	2.39	20.3
rs753172025	2b	0.8153	C/T	<0.01	1	5.43E-06	-0.74	18.32
rs371492921	2b	0.8288	C/T	<0.01	1	1.32E-05	-2.08	14.7
rs750009110	2b	0.7603	C/G	<0.01	1	5.31E-06	2.31	19.1
rs755680404	2b	0.7088	C/G	<0.01	1	1.59E-05	-0.78	18.58
rs201815286	2b	0.7088	C/T	<0.01	0.999	1.00E-04	0.41	17.81
rs753516084	2b	1	G/A	<0.01	0.999	8.55E-05	0.29	19.1
rs1407190036	2b	0.8682	C/A	<0.01	1	6.58E-06	-0.7	19.58

3' UTR

Variant ID	RegulomeDB		Alleles		Allele frequency		GREP	CADD
	Rank	Score		Altered	Ancestral	Altered		
rs745359100	2c	0.74633	C/T	<0.01	1	4.48E-06	0.68	3.135
rs1558956262	2c	0.92	T/A	<0.01	1	4.48E-06	-3.57	4.081
rs746238647	2c	0.92	C/-	<0.01	1	6.57E-06	0.68	-
rs769370864	2a	1	T/G	-	-	-	-4.29	5.098
rs1321440708	2a	0.72927	G/A	<0.01	1	4.52E-06	1.52	10.89
rs1333919675	2b	0.71276	A/G	<0.01	1	6.57E-06	-0.78	15.47
rs571801707	5	0.9223	C/T	<0.01	0.999	2.00E-04	-2.86	2.61
rs777043604	5	0.71614	C/G	<0.01	1	8.46E-06	-4.65	7.281
rs1246626878	3a	0.80781	C/G	<0.01	1	8.46E-06	-4.65	7.281
rs1004083544	3a	0.99633	T/C	<0.01	1	6.57E-06	-5.31	1.087
rs1028379694	2b	0.8662	T/C	<0.01	1	1.31E-05	0.2	7.884
rs974526523	2b	0.8662	T/C	<0.01	1	#######	-5.12	0.265
rs1044257146	3a	0.72733	A/G	<0.01	1	1.97E-05	2.56	9.232
rs938609813	2a	0.80833	A/T	<0.01	1	6.57E-06	0.27	10.56
rs1238436678	2a	1	AAA/AA	<0.01	1	6.57E-06	1.47	-
rs1329233194	2a	1	G/T	<0.01	1	6.57E-06	1.48	11.92
