# Supplementary material for: PRKCE non-coding variants influence on transcription as well as translation of its gene
Source: RNA Biol. 2022 Oct 26;19(1):1115–29. doi: 10.1080/15476286.2022.2139110 (PMC9621080; doi:10.1080/15476286.2022.2139110)
Supplement: Supplemental Material [file ST1.pdf › ST1.pdf]

Supplementary table 1: List of PRKCE 5' and 3' UTR variants retrieved from different variant databases

5' UTR variants

genomAD

Variant ID	Transcript Consequence
rs1443936062	c.-66G>A
rs772170785	c.-51C>T
rs773211026	c.-47C>A
rs1167247806	c.-44delC
rs372046784	c.-34G>A
rs12615066	c.-27C>T
rs12615066	c.-27C>A
rs200186038	c.-24C>G
rs200186038	c.-24C>T
rs766342177	
rs753172025	c.-17dupC
rs934320928	c.-19C>T
rs750009110	c.-17C>G
rs755680404	c.-13C>G
rs201815286	c.-11C>G
rs753516084	c.-10C>T
rs754742928	c.-9G>A
rs893058632	c.-4G>A
rs1485871449	c.-1C>T

EVS

Variant ID	Transcript Consequence
rs372046784	c.-34G>A
rs12615066	c.-27C>T
rs200186038	c.-24C>G
rs766342177	c.-18C>T

ENSEMBL

Variant ID	Chr: bp	Alleles
rs953142436	2:46000904	G/A/C
rs953142436	2:46000904	G/A/C
rs1574178138	2:46000910	G/C
rs569884823	2:46000911	C/G
rs1227344174	2:46000912	T/G
rs1222208258	2:46000924	A/-
rs1287327704	2:46000929	G/C
rs1354941759	2:46000935	C/T
rs1288755171	2:46000936	G/A
rs1238660179	2:46000941	G/A
rs1335719484	2:46000947	T/C
rs1302013310	2:46000951	A/G
rs987625348	2:46000953	G/A
rs912088552	2:46000957	T/C
rs1198801585	2:46000959	C/T
rs1388608323	2:46000966	C/A
rs1259253443	2:46000967	T/A/G
rs1259253443	2:46000967	T/A/G
rs1302848957	2:46000976	C/T
rs946217897	2:46000977	C/G/T
rs946217897	2:46000977	C/G/T
rs1180299648	2:46000995	A/G
rs940538311	2:46001000	C/T
rs1451472245	2:46001001	A/G
rs1363166409	2:46001002	G/A
rs1444088897	2:45651351	G/A
rs1279310031	2:45651352	C/T
rs538954895	2:45651354	C/G/T
rs538954895	2:45651354	C/G/T
rs1299335294	2:45651355	G/A
rs1572856920	2:45651356	G/T
rs1228697459	2:45651358	C/T
rs1363740502	2:45651362	G/A
rs912480755	2:45651364	G/-
rs61762789	2:45651364	G/A
rs1226860990	2:45651365	C/A
rs558553528	2:45651369	C/G
rs1414250098	2:45651372	G/A
rs1297404353	2:45651393	T/C
rs1572856960	2:45651394	G/T
rs1036898545	2:45651396	C/A
rs919336515	2:45651401	C/G
rs929397937	2:45651403	C/G
rs1558526310	2:45651407	C/T
rs1203667990	2:45651408	G/T
rs1184836192	2:45651409	C/T
rs1049077537	2:45651410	C/A/T
rs1049077537	2:45651410	C/A/T
rs1209401734	2:45651412	G/C
rs887745328	2:45651413	C/T
rs113923860	2:45651417	G/A
rs1276515174	2:45651423	G/A
rs148164101	2:45651426	C/T
rs1341045869	2:45651432	C/T
rs1272097179	2:45651437	A/C
rs1229534513	2:45651439	G/A
rs1379339597	2:45651444	G/A
rs1055751280	2:45651452	C/T
rs1447949565	2:45651455	C/T
rs1353913478	2:45651461	G/A
rs1310267095	2:45651471	T/C
rs776148119	2:45651472	G/C
rs1370156637	2:45651483	A/C
rs896768686	2:45651486	T/C
rs1407140099	2:45651489	G/A
rs1414640473	2:45651490	C/T
rs537436368	2:45651494	G/T
rs1558526386	2:45651500	G/T
rs1203735091	2:45651501	A/G
rs1253643130	2:45651503	C/T
rs1182602720	2:45651504	A/G
rs1482628581	2:45651509	C/G/T
rs1482628581	2:45651509	C/G/T
rs61762790	2:45651514	G/A
rs1321725839	2:45651517	A/C
rs1288565577	2:45651518	T/-
rs888821099	2:45651519	G/A/C
rs888821099	2:45651519	G/A/C
rs1333311600	2:45651521	A/T
rs1008778423	2:45651523	C/T
rs1018883365	2:45651528	C/G/T
rs1018883365	2:45651528	C/G/T
rs574540048	2:45651530	G/C
rs1187931975	2:45651535	A/G
rs1422033733	2:45651537	T/C
rs1558526423	2:45651541	C/T
rs1000540686	2:45651547	T/C
rs1350500051	2:45651549	C/G
rs998706962	2:45651553	C/A
rs953800382	2:45651555-45651	CCCCCCC/CCCCCC/CCCCCCCC
rs953800382	2:45651555-45651	CCCCCCC/CCCCCC/CCCCCCCC
rs1572857178	2:45651557	C/A
rs1159895838	2:45651558	C/T
rs1032869877	2:45651559	C/A/T
rs1032869877	2:45651559	C/A/T
rs965329334	2:45651560	C/A/G/T
rs965329334	2:45651560	C/A/G/T
rs965329334	2:45651560	C/A/G/T
rs972544438	2:45651561	C/A/G/T
rs972544438	2:45651561	C/A/G/T
rs972544438	2:45651561	C/A/G/T
rs1229558462	2:45651565	C/A
rs1316628781	2:45651566	C/A
rs918452630	2:45651568	C/A
rs1457185129	2:45651571-45651	GG/G
rs1221104800	2:45651573	T/A/C
rs1221104800	2:45651573	T/A/C
rs1293200978	2:45651574	C/G
rs543265725	2:45651575	A/T
rs931148603	2:45651576	G/C
rs1405481375	2:45651581	A/G
rs1446033604	2:45651584	C/A/T
rs1446033604	2:45651584	C/A/T
rs985257000	2:45651586	C/G/T
rs985257000	2:45651586	C/G/T
rs1290873009	2:45651587	T/A
rs1433602120	2:45651589	A/G
rs938002222	2:45651591	T/A
rs992016379	2:45651595	C/A/T
rs992016379	2:45651595	C/A/T
rs1465811840	2:45651596	G/T
rs915909888	2:45651607	G/A/T
rs915909888	2:45651607	G/A/T
rs1213772661	2:45651608	G/C
rs971806465	2:45651609	G/A
rs1259533182	2:45651615	A/G
rs981682780	2:45651618	T/C
rs687914	2:45651621	G/A/T
rs687914	2:45651621	G/A/T
rs1558526560	2:45651621	G/-
rs1558526579	2:45651622	C/T
rs1558526584	2:45651625	C/G
rs1302882757	2:45651626	C/T
rs902664279	2:45651630	T/A
rs1224086295	2:45651633	C/G/T
rs1224086295	2:45651633	C/G/T
rs1261920018	2:45651640	G/A/C
rs1261920018	2:45651640	G/A/C
rs1000988694	2:45651642	C/T
rs61762791	2:45651644	C/A
rs1217420340	2:45651645	G/A/C
rs1217420340	2:45651645	G/A/C
rs1242340436	2:45651650	A/C
rs1485377731	2:45651653	G/C
rs1193370050	2:45651655	C/A
rs1171280885	2:45651658	A/C
rs1572857394	2:45651659	T/C
rs1450974059	2:45651665	T/C
rs1390200563	2:45651668	C/G/T
rs1390200563	2:45651668	C/G/T
rs1572857415	2:45651671	A/G
rs1572857417	2:45651673	T/G
rs1426258125	2:45651678	A/T
rs1572857429	2:45651679	T/A
rs1261533756	2:45651682	C/G
rs1260255256	2:45651685	G/T
rs1572857439	2:45651688	A/G
rs1184169589	2:45651691	A/G
rs1483745630	2:45651693	G/A
rs774739390	2:45651697	C/T
rs1208941692	2:45651698	G/A
rs1572857461	2:45651700	A/G
rs1344558376	2:45651703	G/A
rs369165780	2:45651904	G/A
rs1237434490	2:45651907	C/T
rs532206195	2:45651909	C/T
rs185312969	2:45651911	G/A
rs952646099	2:45651912	C/T
rs1299198258	2:45651913	G/A
rs1223675490	2:45651916	G/A
COSV100079323	2:45651922	COSMIC_MUTATION
rs144145681	2:45651923	A/G/T
rs144145681	2:45651923	A/G/T
rs1450358402	2:45651924	T/C
rs1399307545	2:45651926	C/T
rs1207275949	2:45651931	G/T
rs958797977	2:45651934	G/A
rs998767594	2:45651936	G/C
rs1054376112	2:45651940	C/T
rs537845343	2:45651941	G/T
rs1558526980	2:45651944	C/A/G
rs1558526980	2:45651944	C/A/G
rs892933923	2:45651947	G/A
rs1013176378	2:45651950	A/G
rs554579350	2:45651952	G/A/T
rs554579350	2:45651952	G/A/T
rs1023026345	2:45651955	G/A
rs1475822634	2:45651956	C/G
rs918001784	2:45651960	G/A/T
rs918001784	2:45651960	G/A/T
rs1443866009	2:45651963	A/T
rs11889760	2:45651967	G/A
rs1002940624	2:45651970	G/A
COSV100078760	2:45651972	COSMIC_MUTATION
rs1345261565	2:45651976	A/T
rs79452669	2:45651980	T/A
rs961363156	2:45651983	G/A/C
rs961363156	2:45651983	G/A/C
rs1270066804	2:45651986	G/T
rs1572857946	2:45651987	T/G
rs553881156	2:45651991	G/C
COSV60313325	2:45651991	COSMIC_MUTATION
rs1429241818	2:45651992	C/T
rs1342595564	2:45651994	A/C
rs1388350686	2:45651998	G/A
rs1414596511	2:45651999	G/A/T
rs1414596511	2:45651999	G/A/T
rs1572857967	2:45652000	G/A
rs1170782953	2:45652003	C/G
rs974262371	2:45652011	C/A
rs1458564640	2:45652013	C/T
rs1329471664	2:45652016	T/A
rs1027663004	2:45652021	C/T
rs983363042	2:45652022	G/C
rs954214615	2:45652028	C/T
rs924195512	2:45652030	G/A/C
rs924195512	2:45652030	G/A/C
rs1418905014	2:45652030-45652	GGG/GGGG
rs1443936062	2:45652035	G/A
COSV100079853	2:45652037	COSMIC_MUTATION
rs1572858030	2:45652040	T/G
rs1258191129	2:45652044	T/A
rs986017109	2:45652046	A/C
rs1186915888	2:45652047	T/A
rs772170785	2:45652050	C/T
rs1259533133	2:45652052	G/A
rs773211026	2:45652054	C/A/T
rs773211026	2:45652054	C/A/T
rs1205442794	2:45652057	C/T
rs1167247806	2:45652057	C/-
rs573424161	2:45652058	G/C
rs1558527129	2:45652064	G/A
rs372046784	2:45652067	G/A
rs771203665	2:45652071	T/G
rs911571016	2:45652073	A/C
rs12615066	2:45652074	C/A/T
rs12615066	2:45652074	C/A/T
rs1359042538	2:45652076	C/T
rs200186038	2:45652077	C/G/T
rs200186038	2:45652077	C/G/T
rs766342177	2:45652080-45652	CCCCC/CCCCCC
rs753172025	2:45652082	C/T
rs371492921	2:45652083	C/T
rs934320928	2:45652084	C/G
rs767145681	2:45652085	A/C/G
rs767145681	2:45652085	A/C/G
rs750009110	2:45652088	C/G
rs755680404	2:45652090	C/G
rs201815286	2:45652091	C/T
rs753516084	2:45652092	G/A
rs1407190036	2:45652093	C/A
rs754742928	2:45652094	C/A
rs900931286	2:45652095	C/T
rs893058632	2:45652097	G/A/C
rs893058632	2:45652097	G/A/C
rs1485871449	2:45652100	C/T

COSMIC

Variant ID	Transcript Consequence
0	

3' UTR variants

genomAD

Variant ID	Transcript Consequence
rs1055560049	c.*10C>T
rs372769511	c.*12G>A
rs755354116	c.*15A>T
rs779297740	c.*18G>A
rs748583576	c.*24C>A
rs748583576	c.*24C>G
rs756787103	c.*29C>T
rs756787103	c.*29C>G
rs1407342066	c.*30C>T
rs745359100	c.*42C>T
rs746238647	c.*43T>A
rs746238647	c.*44delC
rs1321440708	c.*48G>A
rs1328180994	c.*55C>T
rs1333919675	c.*65A>G
rs761990090	c.*3G>A
rs772155036	c.*6C>G
rs571801707	c.*11C>T
rs761011720	c.*13G>T
rs376002289	c.*17G>C
rs777043604	c.*19C>G
rs571539218	c.*25C>T
rs765743344	c.*26G>A
rs1322443197	c.*35A>G
rs940477851	c.*42G>A
rs537772053	c.*44G>T
rs1314269254	c.*46A>G
rs557556854	c.*48A>G
rs767003027	c.*51A>G
rs749879567	c.*52G>C

ENSEMBL

Variant ID	Chr: bp	Alleles
rs938015012	2:46010742	C/A
rs1055560049	2:46010747	C/T
rs372769511	2:46010749	G/A
rs1475690510	2:46010750	C/A
rs1254183218	2:46010751	C/T
rs755354116	2:46010752	A/T
rs779297740	2:46010755	G/A
rs748583576	2:46010761	C/A/G
rs748583576	2:46010761	C/A/G
rs756787103	2:46010766	C/G/T
rs756787103	2:46010766	C/G/T
rs1407342066	2:46010767	C/T
rs886936491	2:46010774	C/G
rs1381946616	2:46010775	T/C
rs745359100	2:46010779	C/T
rs1558956262	2:46010780	T/A
rs746238647	2:46010781	C/-
rs1328765904	2:46010781	C/T
rs772386702	2:46010784-46010789	TGTTTG/TG
rs769370864	2:46010784	T/G
rs1321440708	2:46010785	G/A
rs975614092	2:46010790	C/G
rs1328180994	2:46010792	C/T
rs1558956323	2:46010798	A/T
rs1333919675	2:46010802	A/G
rs1028962764	2:46010804-46010809	CAACAA/CAA
rs1323365703	2:46010804-46010805	CA/-
rs1246626878	2:46010804	C/T
rs1004083544	2:46010811	T/C/G
rs1004083544	2:46010811	T/C/G
rs1284594751	2:46010815	G/A
rs377361438	2:46010819	G/A
rs962973678	2:46010821	C/G
rs751131166	2:46010825	G/A
rs377498969	2:46010832	T/C
rs1276115928	2:46010834-46010839	CTAACT/CT
rs61758295	2:46010838	C/T
rs756914020	2:46010846	C/T
rs1483134616	2:46010858	C/A
rs908576118	2:46010860	C/T
rs1028379694	2:46010874	T/C
rs974526523	2:46010876	T/C
rs376230022	2:46010880	C/T
rs948532411	2:46010883	G/A
rs1180275126	2:46010885	C/G
rs1044257146	2:46010888	A/G
rs986474131	2:46010891	A/T
rs938609813	2:46010892	A/G/T
rs938609813	2:46010892	A/G/T
rs913693685	2:46010894	A/G/T
rs913693685	2:46010894	A/G/T
rs1244029391	2:46010896	G/A
rs1238436678	2:46010900-46010902	AAA/AA
rs1329233194	2:46010904	G/T
rs1289740339	2:46010908	C/G/T
rs1289740339	2:46010908	C/G/T
rs531634050	2:46010916	T/C
rs548459552	2:46010922	A/G
rs184464504	2:46010923	A/T
rs34665948	2: between 46010924 &	-/A
rs1392099843	2:46010934	G/C
rs1388838826	2:46010937	A/T
rs1389085829	2:46010950	G/A
rs527440597	2:46010958	C/T
rs1293423618	2:46010959	A/G
rs547615242	2:46010962	T/G

EVS

Variant ID	Transcript Consequence
rs772386702	c.*17G>C

COSMIC

Variant ID	Transcript Consequence
0	0
